# Supplementary material for: What change in body mass index is associated with improvement in percentage body fat in childhood obesity? A meta-regression
Source: BMJ Open. 2019 Aug 30;9(8):e028231. doi: 10.1136/bmjopen-2018-028231 (PMC6720247; doi:10.1136/bmjopen-2018-028231)
Supplement: Supplementary Appendix 1 [file bmjopen-2018-028231supp001.pdf]

APPENDIX 1: Childhood obesity BMI systematic review_MEDLINE

1. exp Child/
2. exp Adolescent/
3. juvenile.tw.
4. exp Infant/
5. exp Pediatrics/
6. child$.tw.
7. infant$.tw.
8. teen$.tw.
9. p?ediatric$.tw.
10. young person.tw.
11. schoolchild$.tw.
12. youth.tw.
13. (boy$ or girl$).tw.
14. 1 or 2 or 3 or 4 or 5 or 6 or 7 or 8 or 9 or 10 or 11 or 12 or 13
15. exp body weight/
16. exp energy metabolism/
17. exp obesity/
18. exp childhood obesity/
19. exp metabolic syndrome X/
20. exp metabolic disorder/
21. (metabol$ adj1 disorder$).ti,ab.
22. (metabol$ adj1 syndrome$).ti,ab.
23. (cardiometabolic or cardio-metabolic or cardio metabolic).ti,ab.
24. (weight adj3 (cyc$ or reduc$ or los$ or maint$ or decreas$ or watch$ or control$ or gain$ or chang$)).tw.
25. (body fat or body fat percent$ or percent$ body fat or fat mass or adipos$).ti,ab.
26. waist-hip ratio$.tw.
27. waist circumferenc$.ti,ab.
28. (lean adj1 body adj1 mass).ti,ab.
29. (percentage adj1 body adj1 fat).ti,ab.
30. fat.ti,ab.
31. obes$.ti,ab.
32. (overweight or over weight or over-weight).ti,ab.
33. exp abdominal fat/
34. adipose tissue/
35. ((food or energy or calor$) adj1 intake).ti,ab.
36. (BMI or body mass ind$ or body-mass-ind$ or weight for height or weight-for-height).ti,ab.
37. (overfeed$ or over feed$).tw.
38. (overeat$ or over eat$).tw.
39. exp weight gain/
40. exp weight reduction/
41. (weight adj1 los$).ti,ab.
42. (fat adj1 los$).ti,ab.
43. 15 or 16 or 17 or 18 or 19 or 20 or 21 or 22 or 23 or 24 or 25 or 26 or 27 or 28 or 29 or 30 or 31 or 32 or 33 or 34 or 35 or 36 or 37 or 38 or 39 or 40 or 41 or 42
44. (BMI adj5 z score).af.
45. (BMI adj5 SDS).af.
46. (BMI adj5 standard adj1 deviation).af.
47. (Body adj1 mass adj1 index adj5 sd adj1 score).af.
48. (Body adj1 mass adj1 index adj5 SD).af.
49. 44 or 45 or 46 or 47 or 48
50. 14 and 43 and 49
