# Supplementary material for: What change in body mass index is associated with improvement in percentage body fat in childhood obesity? A meta-regression
Source: BMJ Open. 2019 Aug 30;9(8):e028231. doi: 10.1136/bmjopen-2018-028231 (PMC6720247; doi:10.1136/bmjopen-2018-028231)
Supplement: Supplementary Appendix 2 [file bmjopen-2018-028231supp002.pdf]

Appendix 2: Characteristic of studies excluded from meta-regression

	Author, Year, Country (Intervention name)	Study design: Sample size (n) Analysed (An)	Obesity definition	Diet D)/ Exercise (E)/D+E: Setting	Format & content	Adiposity outcome measurement or reasons for exclusion for meta-regression
1.	Carraway 2014[1]<br>USA	Cohort<br>Total = 52 (subgroup n =33 & families offered FU for 10 months)	BMI > 95th %ile	Community	6 x 1 hr nutrition sessions + ad-lib access to a nutritious diet + 7hr/day PA + GT + CBT.	WC only
2.	Croker 2012[2]<br>UK	RCT:<br>Total = 72 (63)<br>FBBT= 37 (33)<br>OC =35 (30)	OW or Ob as per IOTF<br>Mean BMI SDS>3	D+E<br>Clinic (Hospital)	Reduce snacking ≤ 2 occasions/day + Balanced diet following 'Eatwell plate and 'Traffic Light system' + Reduce sedentary behaviour + 60 min/day exercise.	body mass, fat mass, WC &WC-SDS
3.	Doughty 2015[3]<br>USA	Cohort<br>Total = 12	BMI ≥ 89th %ile. Requested data from study authors regarding OB/OW– no response.	D+E<br>Academic Institution	Behavioural counselling + Daily caloric targets + 2 x 1hr/5 days and one 1hr/day physical training) + behavioural counselling.	WC. body mass
4.	Elloumi 2009[4]<br>Tunisia	RCT<br>Total =21<br>Energy restriction =7<br>Exercise training =7<br>Both =7	BMI > 97th %ile (French standards)	D+E<br>Academic Institution	2-month intervention. 3 groups, R = energy restricted group. E=individualised exercise group. RE= energy restriction + exercise. Individualized dietary advice by dietitian. 500kcal/day deficit (15% protein, 55% CHO, 30% fat). Exercise: 90 mins per day; 4 days per week. Intensity of exercise at heart rate corresponding to $lipox_{max}$	Body mass, fat mass
5.	Grulich-Henn, 2011[5]<br>Germany	Cohort Total = 58	BMI > 97th %ile (German paed. standards)	D + E<br>Academic Institution	6 x monthly nutritional consultation & CBT + 24 weekly PA programs.	Body mass only
6.	Gunnarsdottir, 2014[6]<br>Iceland	Cohort Total = 84	BMI z-score > 2.0 SDS (Swedish growth curve)	D+E<br>Clinic (Hospital)	Family-based Epstein behavioural intervention.	Body mass only
7.	Holm 2007[7]<br>Denmark	Cohort<br>Total enrolled =120; BL =110, post-intervention = 87	BMI-SDS LMS method (Danish ref pop.)	D+E<br>Academic Institution	Restricted low-fat diet (6500-7000 kJ/day) + Mandatory and optional PA.	number of pps not consistent for BMI-SDS and % body fat
8.	Kalavainen, 2012[8]<br>Finland	RCT<br>Total = 70<br>routine treatment = 35,<br>group treatment = 35	Wt-for-ht 115-182%	D+E<br>Community	2 interventions (Group and routine) - Routine (2 school heath care sessions) + Group (10 x 90 min/wk parents and children separate focusing on healthy lifestyle/physical activity session, then next 5 sessions/2 wks + 1 session together)	Fat mass only
9.	Kolsgaard, 2011[9]<br>Norway	Cohort<br>Total = 230 analysed (n= 307 started)	BMI > 97.5 %ile for ht according to Norwegian percentiles.	D+E<br>Clinic (Hospital)	~1hr biannual diet & PA (60 min/day exercise encouraged) counselling session with children & parent/s.	Body mass, WC
10.	Kolotourou 2013[10]<br>UK<br>**MEND**	Cohort: Recruited from MEND RCT.<br>Total = 230 analysed<br>Subsample 1 = 71 (6 mth RCT completers – both arms)	BMI ≥ 98th %ile	D+E:<br>Community	Family-based 9-week MEND program (2 x wk group sessions including nutrition education, behaviour modification + fun-based PA) + 12 wk free family swim pass.	WC only

		Subsample 2 = 42 (12 mth RCT completers – IG only)				
11.	Marcano, 2011[11] Venezuela	Cohort Total = 111	OW: BMI >90th %ile/BMI z-score > 1.5. OB: BMI>97th %ile/BMI z-score >2	D+E: Clinic (Hospital)	Nutrition+PA recommendations + A form to register wkly hours of PA, number of steps taken/day, and hrs/wk spent in sedentary activities + Restrict calorie intake and focus on a balanced diet encouraged.	Body fat only
12.	Mager 2015[12]	Cohort Total=12 (completed =9)	CDC criteria	D Clinic (but unclear)	1 session of education for parents and children and then follow up at 3 months and 6 months afterwards.	WC only
13.	Makkes 2016[13] Netherlands	RCT: Total = 80 Short-stay (SS)=40 Long-stay (LS)=40	BMI-SDS ≥3.0 or BMI-SDS ≥2.3 + OB-related comorbidity	D+E: Clinic (Hospital)	Intensive 12-month lifestyle treatment. In-patient period of either 2 months (short-stay group) or 6 months (long-stay group). Short-stay group: biweekly 2-day return visits for 4 months, then monthly 2-day return visits for 6 months following in-pt period. Long-stay group: monthly 2-day return visits for 6 months following in-pt period. Treatment: Nutrition, physical activity and behaviour change. Required active participation of parents/caregivers.	WC,WC-SDS
14.	Martos, 2009[14] Spain (Same intervention as Valle Jimenez 2013[23] but different sample)	Cohort Total = 47	BMI > 95th %ile on growth curves	D+E Community	Moderately OB subjects (Low-calorie diet); Severe/refractory OB subjects (Restriction diet of 25-30%) + Moderate/intense exercise 60 min/day x 5 days/wk encouraged.	Body mass only
15.	Obert 2013[15] France	Cohort Total = 28 (plus 20 healthy lean controls)	BMI > 97th French %ile	D+E: Clinic (Hospital)	Cycle ergometer (9 x 5 mins x 3 times/week: 4 min moderate + 1 min intense) + 2 times/wk moderate exercise for 1st 2 mths	Body mass, fat mass
16.	Panagiotopoulos2011[16] Canada	Cohort Total =119	OB: BMI ≥ 95th %ile; OW: BMI ≥ 85th %ile and <95th %ile with at least 1 comorbidity	D+E: Clinic (Hospital)	10 x consecutive wkly group sessions (6-10 families): 30 min PA + nutrition session + behavioural session.	Body mass only
17.	Pozzato[17] Verduci 2011[18] Italy	Cohort: Total = 26	>30kg/m2 age and sex adjusted Cole *et al* curve	D+E. Community and Clinic	Normocaloric balanced diet and active lifestyle based on italian guidelines for treatment of childhood obesity	WC only
18.	Reinehr, 2004b[19] Germany OBELDICKS	Cohort: Total = 57	BMI ≥ 97th %ile	D+E: Clinic (Hospital)	Obeldicks - Intensive phase 3 mnths (Parents' course 2x/mnth + Behaviour therapy 2x/mnth + Nutritional course 2x/mnth + Exercise therapy 1x/wk) + Establishing phase 3 mnths (Talk rounds for parents 1x/mnth + Psychological therapy + Exercise therapy 1x/wk) + Establishing phase 2 for 3 mnths (Psychological therapy + Exercise therapy 1x/wk) + Establishing phase 3 for 3 mnths (Exercise therapy 1x/wk).	Body fat only
19.	Reinehr, 2009[20] Germany OBELDICKS	Cohort Total = 109 (plus 43 obese controls)	IOTF criteria: OB	D+E: Clinic (Hospital)	Obeldicks (as above)	WC only
20.	Rijks 2015[21] Netherlands	Non-randomised prospective study Total = 172	IOTF criteria: OW, OB, MO	D+E: Clinic (Hospital)	Guidance with focus on nutrition, food habits, PA, sleep, psychological and social aspects.	WC-SDS only
21.	Rovira 2013[22] Spain	Cohort Total = 110	BMI ≥ 97th %ile	D+E Clinic (Hospital)	12 x monthly visits in 2 phases: motivational and intervention. Focus on promoting healthy eating, encouraging PA & decreasing sedentary behaviour.	Only reported as ‘good responders’ and ‘poor responders’ to

						intervention so removed from analysis
22.	Santomauro 2011[23] Venezuela	Cohort Total = 36	BMI > 97th %ile	D+E: Clinic (Hospital)	Dietary recommendations + 30 mins daily moderate exercise or 3 x wk moderate exercise + decrease time watching TV/video games.	Body mass, fat mass
23.	Schum 2012[24] Germany	Cohort: Total = 75 HZ=52 HO= 21	BMI-SDS > 2	E: Community	Increase to 2 hrs/day PA + nutritional recommendations based on 'Optimised Mixed Diet for German Children and Adolescents' + close surveillance by physician.	Fat mass, WC
24.	Shalitin, 2009[25]	Cohort: Total = 174 randomised E =58 (52) D =58 (55) D+E = 58 (55)	BMI > 95th %ile for age & gender	D+E: Clinic (Hospital)	3-month interventions: Exercise intervention (90 min moderate exercise 3 days/wk); Diet intervention 3 mths (12 x/wk nutritional group meetings with parents + Hypocaloric diet 1200 kcal/day); Diet and exercise intervention 3 mths (90 min training session days/wk + 12 x/wk nutritional group meetings with parents + Hypocaloric diet 1200 kcal/day).	SEs were calculated from a mixed model and not directly comparable
25.	Springer 2015[26] Germany	Cohort: Total=39	BMI > 90th %ile	D+E: Clinic (Hospital)	Encouraged to increase exercise by 1-2 hrs/day + Decrease sedentary behaviour to a total of 2 hrs/day or less + Nutrition recommendations + 6 telephone calls from/visits to the physician.	fat mass, WC & WC-SDS
26.	Tan-Ting 2011[27] Philippines	Cohort: Total = 44	BMI ≥ 95th %ile (CDC)	D+E: Clinic (Hospital)	Multidisciplinary, individualised, behavioural modification and exercise programme (St Luke's Medical Center Obesity & Weight Management Program) Dietary session (6 sessions over 3 mths) + Restricted diet (1200-1500 Kcal/day) + Physical activity (24 x 1hr sessions over 3 mths + encouraged to do ≥ 30 min of individual exercise) + Behavioural management (4 x sessions over 3 mths).	body mass, fat mass, WC &WC-SDS
27.	Valle Jiminez 2013[28] Spain	Cohort Total = 50 (plus n=50 non-obese control)	BMI >95th percentile growth curves for Spanish pop.	D+E: Academic Institution/Clinic (Hospital)	Behavioural components, physical exercise and nutritional education. Energy distribution of diet: 25% between breakfast & lunch; 30-35% at lunch; 15% afternoon snack; remainder dinner. Moderate-to-intense PA for 30 mins at least 3 days per wk. Aim that 1 month after the start of tx subjects should be engaging in 60 mins/day moderate-to-intense physical exercise.	Body mass only
28.	Vanhelst 2013[29] France	Cohort Total=37	ND	D+E: Community	2hr/wk exercise sessions + 2hr/3 months heath education session.	Fat mass, fat free mass
29.	Vasquez 2013[30] Chile	Cohort X-over trial (Group 1 only) Total = 60	BMI ≥ 95th %ile CDC	D+E: Academic Institution	Group nutrition education sessions x 6 (5 for children; 1 for parents) Psychologist support sessions x 6 (5 for children; 1 for parents) PE 45 mins x3/wk (30 sessions in total)	Body mass, WC
30.	Verduci 2015[31] Italy	Cohort: Total = 85	BMI Cole's curve cut-off 30 kg/m^2 at 18 yrs	D+E: Clinic (Hospital)	Normocaloric balanced diet + 60 min/day moderate/vigorous exercise + 1 hr educational session with dietician at recruitment.	WC only
31.	Vos, 2011[32] Netherlands	RCT Total = 81 (BL: 79 An. 69) I = 41 (BL 40: An. 36) OC=40 (BL 39: An. 33)	Cole *et al* criteria	D+E: Clinic (Hospital)	12 mths: During first 3 mths (7 x 2.5 hr/2 wks children group meetings + 5 x 2.5 hr/2 wks parent meetings + 1 x 2.5 hr/2 wks child/parent meeting + 2-3 refresher follow-up sessions for total of 2 yrs). Also included exercise however not described except in flow diagram	WC-SDS only
32.	Weiss 2009[33] USA Yale **TEAMS**	Cohort: Total = 186	BMI > 95th %ile (CDC)	D+E: Clinic (Hospital)	Subjects followed biannually as outpatients + Received nutritional/PA guidance. Levels of adherence to these recommendations was not evaluated or documented	Body mass only

33.	Weigel 2008[34] Germany	RCT: Total = 73 IG = 37 OC = 36	OW BMI > 90th %ile OB BMI > 97th %ile Extremely OB BMI > 99.5th %ile	D+E: Community	Twice wkly 45-60 min sessions on exercise/dietary education/coping strategies.	Fat mass only
34.	Wong 2009 [35] USA	Cohort: Total = 21	BMI ≥ 95th %ile	D+E: Community	6 x 1hr behavioural lessons + 4 x 1hr PA/ nutrition lessons + 1800 kcal/day diet.	body mass only

KEY: %ile = percentile; AmO = Outpatient Ambulatory; An. = analysed; apt. = appointment; BB =Bright Bodies; BFC = Better food choices; BL = baseline; BM = behaviour modification; BMI= body mass index; C = control; CG: control group; CBT = cognitive behavioural therapy; CDC = Centre for Disease Control; CG = control group; CHO = carbohydrate; D = diet; E = exercise; FBBT = family-based behavioural treatment; F = female; FU = follow up; GI = glycaemic index; GT = group therapy; HGI = high glycaemic index; hr = hour; HZ = heterozygous; HO = homozygous; ht = height; I = intervention; IG= intervention group; IOTF = International Obesity Task Force; Inpt. = inpatient; LGI = low glycaemic index; LMS= least-mean-squares; LS = long stay; min= minute; mth = month; MO = morbidly obese; norm. normal; n = number; NAFLD = Non-alcoholic fatty liver disease; ND = not described; NR = not reported; OB = obese; OC = obese control; OW = overweight; paed. = paediatric; PA = physical activity; PE = physical activity; PROT= protein; ppts= participants; RCT = randomised controlled trial; SD = standard deviation; SDS = standard deviation score; SE: standard error; SMP= Structured meal plan; SS= short stay; SMC= structured modified carbohydrate diet; trad. = traditional; Trad. act = traditional activity; tx = treatment; TEAMS = Tracking Endpoints in Adolescent MS; wk = week; WList OC– wait list obese control; WL = weight loss; wt = weight; X-over = crossover; yr = year

1. Carraway ME, Lutes LD, Crawford Y, et al. Camp-based immersion treatment for obese, low socioeconomic status, multi-ethnic adolescents. *Childhood obesity* (Print). 2014;10(2): 122-131

2. Croker H, Viner RM, Nicholls D. Family-based behavioural treatment of childhood obesity in a UK National Health Service setting: randomized controlled trial. *International Journal of Obesity*. 2012; 36(1):16-26

3. Doughty KN, Njike VY, Katz DL. Effects of a Cognitive-Behavioral Therapy-Based Immersion Obesity Treatment Program for Adolescents on Weight, Fitness, and Cardiovascular Risk Factors: A Pilot Study. *Childhood Obesity.* 2015;11(2):215-218

4. Elloumi M, Ben Ounis O, Makni E, Van Praagh E, Tabka Z, Lac G. Effect of individualized weight-loss programmes on adiponectin, leptin and resistin levels in obese adolescent boys. *Acta Paediatr.* 2009; 98(9):1487-93

5. Grulich-Henn J, Lichtenstein S, Hörster F, Hoffmann GF, Nawroth PP, Hamann A. Moderate weight reduction in an outpatient obesity intervention program significantly reduces insulin resistance and risk factors for cardiovascular disease in severely obese adolescents. *Int J Endocrinol.* 2011:541021

6. Gunnarsdottir T, Einarsson SM, Njardvik U, et al. Family-based behavioral treatment for obese children - results and two year follow up. *Laknabladid*. 2014;100(3):139-45

7. Holm JC, Buckert M, Hans-Christoph Friederich H-C, et al. Time course and determinants of leptin decline during weight loss in obese boys and girls. *International Journal of Pediatric Obesity*. 2007; 2(1): 2-10

8. Kalavainen M, Utriainen P, Vanninen E, Korppi M, Nuutinen O. Impact of childhood obesity treatment on body composition and metabolic profile. *World Journal of Pediatrics*. 2012; 8(1):31-7

9. Kolsgaard ML, Joner G, Brunborg C, et al. Reduction in BMI z-score and improvement in cardiometabolic risk factors in obese children and adolescents. The Oslo Adiposity Intervention Study - a hospital/public health nurse combined treatment.[Erratum appears in BMC Pediatr. 2012;12:77; PMID: 22709339]. *BMC Pediatrics.* 2011; 11: 47

10. Kolotourou M, Radley D, Gammon C, Smith L, Chadwick P, Sacher PM. Long-Term Outcomes following the MEND 7-13 Child Weight Management Program. *Childhood Obesity*. 2015;11(3): 325-330

11. Marcano, H, Fernández M, Paoli M, et al. Limited Weight Loss or Simply No Weight Gain following Lifestyle-Only Intervention Tends to Redistribute Body Fat, to Decrease Lipid Concentrations, and to Improve Parameters of Insulin Sensitivity in Obese Children. *International Journal of Pediatric Endocrinology*. 2011(1): 241703

12. Mager DR, Iñiguez IR, Gilmour S, Yap J. The effect of a low fructose and low glycemic index/load (FRAGILE) dietary intervention on indices of liver function, cardiometabolic risk factors, and body composition in children and adolescents with non-alcoholic fatty liver disease (NAFLD). *J Parenter Enteral Nutr.* 2015;39(1):73-84

13. Makkes S, Renders CM, Bosmans JE, van der Baan-Slootweg OH, Hoekstra T, Seidell JC. One-year effects of two intensive inpatient treatments for severely obese children and adolescents. *BMC Pediatrics*. 2016; 16:120 doi: 10.1186/s12887-016-0659-x

14. Martos R, Valle M, Morales RM, Cañete R, Gascón F, Urbano MM. Changes in body mass index are associated with changes in inflammatory and endothelial dysfunction biomarkers in obese prepubertal children after 9 months of body mass index SD score loss. *Metabolism-Clinical and Experimental.* 2009; 58(8):1153-1160

15. Obert P, Gueugnon C, Nottin S, et al. Impact of diet and exercise training-induced weight loss on myocardial mechanics in severely obese adolescents. *Obesity*, 2013; 21(10): 2091-8

16. Panagiotopoulos C, Ronsley R, Al-Dubayee M, et al. The Centre for Healthy Weights-Shapedown BC: A Family-Centered, Multidisciplinary Program that Reduces Weight Gain in Obese Children over the Short-Term. *International Journal of Environmental Research and Public Health.* 2011; 8(12):4662-4678

17. Pozzato C, Verduci E, Scaglioni S, Liver Fat Change in Obese Children After a 1-year Nutrition-behavior Intervention. Journal of Pediatric Gastroenterology and Nutrition. 2010;51(3), 331–335

18. Verduci E, Radaelli G, Salvioni M, Riva E, Giovannini M. Plasma long-chain fatty acids profile and metabolic outcomes in normolipidaemic obese children after one-year nutritional intervention. *Acta paediatrica*. 2011;100(4): 585-9

19. Reinehr T. and Andler W. Changes in the atherogenic risk factor profile according to degree of weight loss. *Archives of Disease in Childhood*. 2004;89(5):419-422

20. Reinehr T, C Schmidt C, Toschke AM, Andler W. Lifestyle intervention in obese children with non-alcoholic fatty liver disease: 2-year follow-up study. *Archives of Disease in Childhood.* 2009; 94(6):437-442

21. Rijks JM, Plat J, Mensink RP, Dorenbos E, Buurman WA, Vreugdenhil AC. Children with morbid obesity benefit equally as children with overweight and obesity from an ongoing care program. *Journal of Endocrinol Metab*. 2015; 100(9): 3572-3580

22. Rovira AF, París Miró N, Zaragoza-Jordana M, et al. Clinical and metabolic effectiveness of a new motivational therapy for the treatment of obesity in adolescents (OBEMAT). *Anales De Pediatria.* 2013; 78(3): 157-166

23. Santomauro M, Paoli-Valeri M, Fernández M, et al. Non-alcoholic fatty liver disease and its association with clinical and biochemical variables in obese children and adolescents: Effect of a one-year intervention on lifestyle. *Endocrinología y Nutrición* (English Edition), 2012;59(6): p. 346-353

24. Schum J, Blumenstock G, Weber K, et al. Variants of the FTO Gene in Obese Children and their Impact on Boy Composition and Metabolism before and after Lifestyle Intervention. *Experimental and Clinical Endocrinology & Diabetes*. 2012;120(3):128-131

25. Shalitin S, Ashkenazi-Hoffnung L, Yackobovitch-Gavan M, et al. Effects of a Twelve-week randomized intervention of exercise and/or diet on weight loss and weight maintenance, and other metabolic parameters in obese preadolescent children. *Hormone Research*. 2009;72(5):287-301

26. Springer F, Ballweg V, Schweizer R, et al. Changes in whole-body fat distribution, intrahepatic lipids, and insulin resistance of obese adolescents during a low-level lifestyle intervention. *European Journal of Pediatrics*, 2015;174(12):1603-12

27. Tan-Ting AM, Llido L. Outcome of a hospital based multidisciplinary weight loss program in obese Filipino children. *Nutrition.* 2011; 27:50-54

28. Valle Jimenez M, Martos R, Morales RM, et al. Relationship between changes in plasma leptin concentrations and plasminogen activator inhibitor-1 in obese prepubertal children after nine months of treatment. *Annals of nutrition & metabolism.* 2013; 63(3): 216-22

29. Vanhelst J, Fardy PS, Béghin L, Bui-Xuan G, Mikulovic J. Strategies in intervention programmes for obese youth: implication of the age and the type of physical activities. *Clinical Physiology and Functional Imaging.* 2015; 35(1): 17-20

30. Vasquez F, Diaz E, Lera L, Vasquez L, Anziani A, Burrows R. Impact of a strength training exercise program on body composition and cardiovascular risk factors in a group of obese schoolchildren by pubertal stage. *American Journal of Sports Science and Medicine.* 2014; 2(1): 40-47

31. Verduci E, Lassandro C, Giacchero R, Miniello VL, Banderali G, Radaelli G. Change in metabolic profile after 1-year nutritional-behavioral intervention in obese children. *Nutrients.* 2015; 7(12): 10089-10099

32. Vos RC, Wit JM, Pijl H, Houdijk EC. Long-term effect of lifestyle intervention on adiposity, metabolic parameters, inflammation and physical fitness in obese children: a randomized controlled trial. *Nutrition & Diabetes.* 2011; 1:e9. doi: 10.1038/nutd.2011.5

33. Weiss R, Shaw M, Savoye M, Caprio S. Obesity dynamics and cardiovascular risk factor stability in obese adolescents. *Pediatric Diabetes.* 2009; 10(6): 360-7

34. Wiegal C, Kokocinski K, Lederer P, Dötsch J, Rascher W, Knerr I. Childhood obesity: Concept, feasibility, and interim results of a local group-based, long-term treatment program. *J Nutr Educ Behav.* 2008; 40:369-373

35. Wong WW, Abrams SH, Mikhail C, et al. An innovative summer camp program improves weight and self-esteem in obese children. *Journal of Pediatric Gastroenterology and Nutrition.* 2009; 49(4):493-497
