# Supplementary material for: What change in body mass index is associated with improvement in percentage body fat in childhood obesity? A meta-regression
Source: BMJ Open. 2019 Aug 30;9(8):e028231. doi: 10.1136/bmjopen-2018-028231 (PMC6720247; doi:10.1136/bmjopen-2018-028231)
Supplement: Supplementary Appendix 3 [file bmjopen-2018-028231supp003.docx]

***Appendix 3: Meta-regression line and 95% prediction interval for the relationship between the mean change in percentage Body Fat and BMI-SDS across the 39 studies (66 subsets), as in Figure 3, but highlighting four subgroups of younger participants:***

***● Gajewska et al.^37^ two groups aged 5-10y;***

***● Dobe et al.^46^ aged 4-8y;***

***● Kirk et al.^47^ group 1, aged 5-10y***
