# Supplementary material for: What change in body mass index is associated with improvement in percentage body fat in childhood obesity? A meta-regression
Source: BMJ Open. 2019 Aug 30;9(8):e028231. doi: 10.1136/bmjopen-2018-028231 (PMC6720247; doi:10.1136/bmjopen-2018-028231)
Supplement: Author's manuscript [file bmjopen-2018-028231.draft_revisions.pdf]

BMJ Open is committed to open peer review. As part of this commitment we make the peer review history of every article we publish publicly available.

When an article is published we post the peer reviewers' comments and the authors' responses online. We also post the versions of the paper that were used during peer review. These are the versions that the peer review comments apply to.

The versions of the paper that follow are the versions that were submitted during the peer review process. They are not the versions of record or the final published versions. They should not be cited or distributed as the published version of this manuscript.

BMJ Open is an open access journal and the full, final, typeset and author-corrected version of record of the manuscript is available on our site with no access controls, subscription charges or pay-per-view fees (http://bmjopen.bmj.com).

If you have any questions on BMJ Open's open peer review process please email info.bmjopen@bmj.com

BMJ Open

What change in body mass index reduces body fat in childhood obesity: a meta-regression.

Journal:	*BMJ Open*
Manuscript ID	bmjopen-2018-028231
Article Type:	Research
Date Submitted by the Author:	27-Nov-2018
Complete List of Authors:	Birch, Laura; NIHR Bristol Biomedical Research Centre - Nutrition<br>Perry, Rachel; NIHR Bristol Biomedical Research Centre - Nutrition<br>Hunt, Linda<br>Matson, Rhys; NIHR Bristol Biomedical Research Centre - Nutrition<br>Chong, Amanda; NIHR Bristol Biomedical Research Centre - Nutrition<br>Beynon, Rhona; NIHR Bristol Biomedical Research Centre - Nutrition<br>Shield, Julian; NIHR Bristol Biomedical Research Centre - Nutrition
Keywords:	obesity, childhood, adolescence, body mass index, body fat

SCHOLARONE™
Manuscripts

What change in body mass index reduces body fat in childhood obesity: a meta-regression.

Laura Birch[a], RD MSc, Rachel Perry[a], PhD, Linda P Hunt[a], PhD, Rhys IB Matson[a], MSc, Amanda Chong[a], BSc, Rhona Beynon[a], MSc, Julian Hamilton-Shield[a,b] MD

Affiliations: [a] NIHR Bristol Biomedical Research Centre – Nutrition Theme, Level 3 University Hospitals Bristol Education Centre, Upper Maudlin Street, Bristol.

[b] University Hospitals Bristol NHS Foundation Trust, Level 6 University Hospitals Bristol Education Centre, Upper Maudlin Street, Bristol.

Address correspondence to: Laura Birch, NIHR Bristol Biomedical Research Centre – Nutrition Theme, Level 3 University Hospitals Bristol Education Centre, Upper Maudlin Street, Bristol, UK, BS2 8AE, [laura.birch@bristol.ac.uk], +44 (0)117 3421763

Rachel.Perry@bristol.ac.uk
L.P.Hunt@bristol.ac.uk
Rhys20@me.com
ac14629@bristol.ac.uk
rhona.beynon@bristol.ac.uk
J.P.H.Shield@bristol.ac.uk

Short title: BMI change to reduce body fat in childhood obesity

Financial Disclosure: The authors have no financial relationships relevant to this article to disclose.

Funding Source: This study was supported by the NIHR Biomedical Research Centre at University Hospitals Bristol NHS Foundation Trust and the University of Bristol. The views expressed in this publication are those of the authors and not necessarily those of the NHS, the National Institute for Health Research or the Department of Health.

Conflict of Interest: Professor Hamilton-Shield is a lead author on two studies included in the systematic review that this paper reports on. The other authors have no conflicts of interest to disclose.

Key words: obesity, childhood, adolescence, body mass index, body fat

Abbreviations:
BMI-SDS: body mass index- standard deviation score
RCT: randomised controlled trial
IOTF: International Obesity Task Force
HOMA: Homeostatic model assessment
LDL: Low-density lipoprotein
HDL: High-density lipoprotein
IL6: Interleukin 6
ALT: Alanine transaminase
HTA: Health Technology Assessment
SD: standard deviation
SE: standard error

For peer review only - http://bmjopen.bmj.com/site/about/guidelines.xhtml

CI: Confidence interval
PI: Prediction interval
IQR: Interquartile range

Word count: 3925

For peer review only

For peer review only - http://bmjopen.bmj.com/site/about/guidelines.xhtml

Abstract

Objective: Using meta-regression this paper sets out the minimum change in BMI-SDS/z-score required to improve adiposity as percentage body fat for obese children and adolescents.

Design: Meta-regression.

Setting: Studies were identified as part of a large-scale systematic review of the following electronic databases: AMED, Embase, MEDLINE via OVID, Web of Science and CENTRAL via Cochrane library.

Participants: Individuals aged 4–19 years with a diagnosis of obesity according to defined BMI thresholds.

Interventions: Studies of lifestyle treatment interventions that included dietary, physical activity and/or behavioural components with the objective of reducing obesity were included. Interventions of less than 2 weeks duration and those that involved surgical and/or pharmacological components (e.g. bariatric surgery, drug therapy) were excluded.

Primary and secondary outcome measures: To be included in this review, studies had to report baseline and post-intervention BMI-SDS/z-score or change measurements (primary outcome measures) plus one or more of the following markers of metabolic health (secondary outcome measures): adiposity measures other than BMI; blood pressure; glucose; inflammation; insulin sensitivity/resistance; lipid profile; liver function. This paper focuses on the adiposity measures only. Further papers in this series will report on other outcome measures.

Results: This paper explores the potential impact of BMI-SDS reduction in terms of change in percentage body fat. Thirty-nine studies reporting change in mean percentage body fat were analysed. Meta-regression demonstrated that reduction of at least 0.6 in mean BMI-SDS ensured a mean reduction of percentage body fat mass, in the sense that the associated 95% prediction interval for change in mean percentage body fat was wholly negative.

Conclusions: Interventions demonstrating reductions of 0.6 BMI-SDS might be termed successful in reducing adiposity; a key purpose of weight management interventions.

Trial registration: The main review is registered on PROSPERO international prospective register of systematic reviews (PROSPERO 2016 CRD42016025317).

For peer review only

Article Summary

Strengths and limitations of this study

- We believe that this is the first paper to attempt to bring together all studies that have reported both a change in BMI-SDS/z-score and changes in a marker of adiposity in the obese paediatric population.
- The systematic methods employed to identify the included studies were stringent, but it is possible that some relevant studies might have been missed.
- There was some variation in the reporting of results where there were multiple publications of the same study; in these cases, the results from the most comprehensive paper have been used.
- Studies that did not report change in mean percentage body fat could not be included in this meta-regression.

Introduction

Childhood obesity is one of the most serious global public health challenges of the twenty-first century[1]. In England, the latest figures from the National Child Measurement Programme, which measures the height and weight of around one million school children every year, showed that 9.6 percent of children aged 4–5 years and 20 percent of those aged 10–11 years were obese[2,3]. Childhood obesity has adverse health consequences in both the short-and long-term (an increased risk of developing metabolic disturbances, including hypertension, dyslipidaemia and insulin resistance, and becoming obese adults[4]). The presence of adverse changes in cardiac and vascular function and type 2 diabetes, which were previously considered adult morbidities, now being identified in obese children and adolescents[5-11] illustrates the urgent need for effective weight management treatment interventions to improve the metabolic health status of the paediatric population.

Moderate weight loss has been shown to have a positive impact on many metabolic and cardiovascular risk factors[12,13]. Weight management interventions for obese adults that result in a 5-10 percent decrease in body weight are associated with significant improvements in blood pressure, serum lipid levels and glucose tolerance[14] and reduction in the prevalence of

For peer review only

hypertension and diabetes[15]. Minimum weight management targets can therefore be set to improve metabolic health in this population[16].

During childhood, all measurements over time are complicated by the influence of growth, meaning that cut-offs routinely used in the adult population cannot be used in children and adolescents. However measured values of body mass index (BMI) can be standardized into z-scores in respect to reference populations[2,3] (BMI z-scores) or standard deviation scores (BMI-SDS). The degree of overweight is quantified using Cole's box cox-transformation, which normalizes the BMI skewed distribution in childhood and expresses BMI as a standard deviation score (BMI-SDS)[17]. These standardised scores provide a normalised measurement for the degree of obesity in children and young people, indicating to what degree an individual BMI lies above or below the median BMI value.

A meta-analysis by Ho *et al* [18] concluded that lifestyle interventions can lead to improvements in weight and cardio-metabolic outcomes in child obesity. However, whilst numerous lifestyle intervention programmes to tackle childhood obesity are conducted across the UK, and many describe statistically significant reductions in BMI-SDS[19], these results do not necessarily translate into clinical benefit for the individual. The clinical significance of the reduced BMI-SDS in terms of improvements in adiposity and metabolic profile is uncertain. The amount of weight reduction needed to effect beneficial change in metabolic health in the paediatric population is largely unknown.

Paediatric weight management guidelines exist in many countries to promote best practice, but at present many of these recommendations are based on low-grade scientific evidence[20]. Understanding how much BMI must be reduced to positively affect metabolic health is important

For peer review only - http://bmjopen.bmj.com/site/about/guidelines.xhtml

to ensure that treatment interventions are appropriately designed and evaluated[21]. Interventions must effect beneficial change in relation to cardiometabolic risk factors, rather than just statistical significance, if they are to be considered successful.

Given the scale of the obesity problem and the significant and sustained adverse effects on health, clinically effective paediatric weight management treatment options are vital. A meta-analysis of cardiovascular disease risk in healthy children and its association with BMI has been conducted[22] but there is yet to be a systematic quantification of the reduction in BMI or adiposity needed to achieve improvements in metabolic health in the obese paediatric population.

It is important to highlight that when assessing interventions designed to manage overweight and obesity in children and adolescents, it is essential to recognise that measures such as BMI and derived SDS scores are surrogates of the real purpose: reduction of adiposity, fat being the key organ involved in metabolic complications[23]. To rigorously assess the clinical and cost effectiveness of weight management interventions in young people, it is first necessary to understand what BMI-SDS change means in terms of key outcomes such as effects on adiposity and metabolic health. This paper is designed to put BMI-SDS changes in context when considering clinical efficacy. Through meta-regression analysis we explore the potential impact of BMI-SDS reduction in terms of change in percentage body fat. The outcome of which will both inform clinical guidelines for paediatric weight management interventions and guide outcome measures in future clinical trials.

Objective

This paper aims to establish the minimum change in BMI-SDS needed to effect improvements in adiposity markers of obese children and adolescents. This is the first of a series of three papers

For peer review only - http://bmjopen.bmj.com/site/about/guidelines.xhtml

reporting on the findings from studies identified in a large systematic review (N=90 studies; searched up to May 2017) and focuses on the evidence in relation to adiposity (percentage body fat).

Methods

The studies included in this paper were identified as part of large-scale systematic review (PROSPERO CRD42016025317). The protocol for this systematic review is available: https://doi.org/10.1186/s13643-016-0299-0. The review was completed in January 2018 and the results are still being evaluated.

Participants

Studies with participants aged 4–19 years with a diagnosis of obesity using defined BMI thresholds were considered for inclusion. BMI-SDS was calculated as a function of the degree of obesity of the subjects when compared with BMI standards. BMI standards included, but were not limited to, the 98th centile on the UK 1990 growth reference chart[24], 95th percentile on the US Centre for Disease Control and Prevention growth chart[25], the International Obesity Taskforce (IOTF) BMI for age cut-points[26] and the World Health Organisation growth references[27,28], in addition to country-specific obesity thresholds using BMI reference data from their paediatric populations. Studies that included overweight, as opposed to obese, individuals, pregnant females, or those with a critical illness, endocrine disorders or syndromic obesity were excluded from this review.

Interventions

Studies of lifestyle treatment interventions that included dietary, physical activity and/or behavioural components with the objective of reducing obesity were included. Interventions of

For peer review only - http://bmjopen.bmj.com/site/about/guidelines.xhtml

less than 2 weeks duration and those that involved surgical and/or pharmacological components (e.g. bariatric surgery, drug therapy) were excluded. Studies focused on obesity prevention were also excluded. No restrictions were imposed regarding the setting or delivery of the interventions.

Outcome measures

To meet the inclusion criteria interventions had to report baseline (pre-) and post-intervention BMI-SDS/z-score or change measurements of BMI-SDS/z-score plus one or more of the following markers of metabolic health:

- Adiposity measures other than BMI (including waist circumference and percentage body fat)
- Glucose
- Insulin sensitivity/resistance (homeostatic model assessment (HOMA))
- Lipid profile (triglycerides, total cholesterol, low-density lipoprotein (LDL)/high-density lipoprotein (HDL) cholesterol)
- Inflammation (C-reactive protein)
- Blood pressure (systolic, diastolic)
- Liver function

This paper focuses on the adiposity measures only. Further papers in this series will report on other outcome measures.

Study design

Completed, published, randomised controlled trials (RCTs) and non-randomised studies (cohort studies) of lifestyle treatment interventions for obese children and adolescents, with or without follow-up.

For peer review only - http://bmjopen.bmj.com/site/about/guidelines.xhtml

Ethics

Ethical approval was not required as this paper reviewed published studies only.

Information sources and search methods

Studies were identified by searching five electronic databases from inception to May 2017 (AMED, Embase, MEDLINE via OVID, Web of Science and CENTRAL via Cochrane library), alongside scanning reference lists of included articles and through consultation with experts in the field. The search strategy for MEDLINE database is presented in Supplementary File 1.

Study Selection and data extraction

Titles and abstracts were assessed for eligibility and the data outcome measures described previously were extracted by two independent reviewers from the review team (LB, AC, RP, RB) using a standardised data extraction template, which was piloted by both reviewers before starting the review to ensure consistency.

Quality assessment

The focus of our study is the relationship between change in BMI-SDS and change in metabolic health parameters, rather than the specific treatment interventions that effect these changes. Therefore, risk of bias tools, such as the Cochrane Risk of Bias tool,[29] were not considered appropriate. The included studies were assessed for methodological quality by two members of the review team during the data extraction process using the Quality Assessment tool utilised in the 2004 Health Technology Assessment (HTA) systematic review of the long-term effects and economic consequences of treatments for obesity and implications for health improvement[30]. This Quality Assessment tool comprises 20 questions which are added together to give a final

For peer review only

For peer review only - http://bmjopen.bmj.com/site/about/guidelines.xhtml

score and a percentage rating, from which a level of quality is assigned. Any discrepancies in Quality Assessment scoring were resolved through discussion.

Analysis

We carried out random-effects meta-regression as implemented in Stata[31] to try to quantify the relationship between mean change in BMI-SDS/z-score (independent, predictor variable) and mean change in percentage body fat (target variable), where these were either reported, or were able to be calculated from reported data. Further details are given below. We were not trying to assess the relative effects of the various interventions, but rather to examine the relationship between these two outcomes. Meta-regression allows for residual heterogeneity in the target variable not explained by the predictor. Subsets from the same study (e.g. intervention vs control, boys vs girls) were regarded as independent observations provided there was no data duplication.

Results

Search Results

In total, 98 published articles relating to 90 different studies met the inclusion criteria for the entire systematic review. See Figure 1 for a flow diagram illustrating the number of papers excluded at each stage of the review. For studies reported in multiple publications, the reference that provided the most comprehensive information has been used (see Table 1 for details).

Figure 1: Flow diagram from the systematic review that identified the included studies

The Venn diagram (Figure 2) illustrates how many studies were identified for the various markers of metabolic health. Seventy-three studies assessed and reported adiposity measures. The adiposity measures reported included percentage body fat, body fat-SDS, body mass, fat

For peer review only - http://bmjopen.bmj.com/site/about/guidelines.xhtml

mass, fat-free-mass, waist circumference and waist circumference-SDS. The 68 studies that examined diabetes/inflammation measures (HOMA, insulin, glucose, C-reactive protein, Interleukin-6 (IL6), Alanine transaminase (ALT) and the 71 studies examining cardiac measures (e.g. lipids, cholesterol, blood pressure) will be reported separately

Figure 2: Venn diagram illustrating the markers of metabolic health measured

Studies for inclusion in meta-regression analysis

Seventy-three studies assessed and reported adiposity measures. Of the different adiposity measures that were reported in the 73 adiposity studies (percentage body fat, body fat-SDS, body mass, fat mass, fat-free-mass, waist circumference and waist circumference-SDS), we elected to examine percentage body fat as it was far more frequently reported across studies. Therefore, of these 73 adiposity studies, we conducted our meta-regression on 39 studies which reported percentage body fat values. These studies are presented in Table 1 with the corresponding changes in BMI-SDS/z-score. The characteristics of the remaining studies that were excluded from the meta-regression are summarised in Appendix 2.

For peer review only

Table 1: Characteristics of studies reporting adiposity outcomes with results of mean change in BMI-SDS and percentage body fat

	**Author, Year, Country (Intervention name)**	**Study design: Sample size (n) Analysed (An)**	**Obesity definition**	**Age range (inclusion): Mean ± (SD) Sex (% F)**	**Pubertal status measured**	**Diet D)/ Exercise (E)/D+E: Setting**	**Format & content**	**Duration (mths): Follow up (mths)**	**Δ BMI SDS/z-score by subgroup when reported**	**Δ % body fat score by subgroup when reported**
1	Bell 2007[32]<br>Australia	Cohort<br>Total = 14	BMI ≥ 95th %ile	Age range:9-16<br>12.70(2.32); F=43%	Yes-Tanner	E:<br>community	8 weeks structured circuits exercise training: 3 x 1hr sessions/week. No standard dietary modifications.	2:<br>0	All:-0.03	All:-0.57
2	Bock 2014[33]<br>Canada<br>**HIP KIDS**	Cohort:<br>Total = 42 (41)	BMI ≥ 95th %ile (CDC)	Age range: 8-17<br>12.8 ± 3.14; F=50%	Yes - Tanner	D+E:<br>Clinic (Hospital)	Intensive phase (3 mths): bi-wkly 90 min counselling. Maintenance phase (9 months): alternating mthly GP or individual sessions (90 mins). Sessions focus on exercise/psychosocial/behavioural aspects.	12:<br>0	All: -0.04	All: -1.39
3	Bruyndonckz, 2015[34]<br>Belgium	Quasi-RCT:<br>Total = 61<br>IG = 33<br>CG = 28	BMI ≥ 97th %ile adolescents <16 yrs; BMI ≥ 35 adolescents ≥ 16 yrs	Age range:12-18<br>IG: 15.4 ± 1.5; F = 75%<br>CG: 15.1 ± 1.2; F=75%	NR	D+E:<br>Clinic (Hospital)	Intervention: Dietary restriction 1500-1800 kcal/day + 2 hrs/day supervised play/lifestyle activities + 2hrs/wk PE + 3 x 40min/wk supervised training session.<br>Control: Usual care.	10:<br>0	IG: -1.21<br>CG: 0.13	IG: -11.30<br>CG: 0.4
4	Bustos 2015[34]<br>Chile	Cohort:<br>Total = 50 (28 completed)	CDC	Age range: NR<br>9.5 ± 1.9; F=47.6%	NR	D+E:<br>Academic Institution	Nutrition/behavioural modification session 40 min/wk + PA 50 min x2/wk+ Family support every 15 days for first 2 mths, then mthly.	8:<br>0	All: -0.3	All: -3.00
5	Calcaterra 2013[36]<br>Italy	Cohort:<br>Total = 22	BMI > 95th %ile	Age range: 9-16<br>13.23 ± 1.76; F=41%	Yes - Tanner	E:<br>Academic Institution	2 x 90 mins exercise training sessions/wk	3:<br>0	All: -0.15	All: -3.30
6	Dobe 2011[37]<br>Germany<br>OBELDICKS – mini	Cohort:<br>Total = 103	>97th to 99.5 percentile	Age range: 4-8<br>6.1 ± 1<br>F=56%	NR	D+E:<br>Academic Institution	Obeldicks mini: focus on training parents (22.5 hrs for parents, 4.5 hrs for children). Group sessions. Parents+children classes every 4th session,<br>Children's classes: 9 x monthly sessions (30 mins): 1 x introduction; 3 x diet; 5 x eating habits<br>Parenting classes: 13 x monthly sessions (1.5 hrs): 1x introduction 1x medicine 3x nutrition 5x eating habits + education tips<br>3x discussion circle<br>Individual consultation: every 2 months (30 mins)<br>Exercise: 50 x weekly sessions (1.5 hrs)	12:<br>0	Obeldicks-mini: -0.46	Obeldicks mini: -3.00
7	Farpour-Lambert, 200938Switzerland	RCT:<br>Total = 44<br>IG= 22<br>OC =22	BMI > 97th %ile	Age range: 6-11<br>8.9 ± 1.5<br>IG: F=59%<br>OC: F= 68%	Yes	E<br>Clinic (Hospital)	180 min/wk PA + 135 min/wk PE	3:<br>0	IG: -0.1<br>CG: 0	IG: -1.50<br>CG: 0.80
8	Ford, 2010a,b UK [39,40]	RCT:<br>Total = 106	BMI ≥ 95th %ile (CDC)	Mandometer: 9.0 - 16.9<br>SC: 9.1 - 17.5<br>Mandometer: 12.7 ± 2.2<br>SC: 12.5 ± 2.3<br>F=56%	Yes	D<br>Clinic (Hospital)	Mandometer device to regulate rate of eating and total intake vs SC	12:<br>0	IG:-0.36<br>CG:-0.14	IG:-4.60<br>CG:-1.30

For peer review only - http://bmjopen.bmj.com/site/about/guidelines.xhtml

9	Gajewska, 2016[41] Poland	Cohort: Total = 100	BMI SDS > 2	Age range: 5-10 with WL: 8.1(6.8-9.2); F= 55% without WL: 8,8(7,3-9.6); F=53%	reported with Tanner stage, any with pubertal develop-ment excluded.	D+E: Community & Academic institution	3-mth intervention, low energy diet (1200-1400kcal), 3-5 meals every day, instructions concerning PA, 10-14 food day diary, 3-day food diary.	3: 0	WL: -0.98 No WL:-0.2	WL:-2.90 No WL:0.30
10	Garanty-Bogacka, 2011[42] Poland	Cohort: Total = 50	BMI > 97th %ile (Polish ref pop.)	Age range:8-18 14.2 ± 2.6; F=58%	Yes	D+E: Clinic (Hospital)	Exercise therapy (Instructions in PA + reducing sedentary behaviour) + Reduction in fat and sugar intake.	6: 0	All:-1	All: -4.70
11	Grønbæk 2012[43] & Kazankov 2014[44] Denmark **Julemaerkehjem met Hobro (same cohort)**	Cohort: Total = 117 (n=71 attended 12 mth FU)	NR. Obese. BL BMI-SDS: 2.93±0.52	Age range: NR 12.1 ± 1.3 F=56%	NR	D+E: Community	Individually designed healthy diet + moderately strenuous PA program (at least 1hr/day).	2.5 month s/10 weeks: 12mth FU	All: -0.63	All:-4.30
12	Hvidt 2014[45] Denmark	Cohort: Total = 61	Children's Obesity Clinic; BMI > 90th %ile (Danish ref pop.) = z-score 1.28. BL BMI-SDS: 2.73±0.60	Age range:10-18 Median: 12.5 F=54%	NR	D+E: Clinic (Hospital)	Family-centred approach involving behaviour changing techniques (90 advice and advice strategies on low-calorie diet + activity e.g. 10-20 items aimed to reduce obesity).	12: 0	All: -0.21	All: -3.40
13	Kirk, 2005[46] USA	Cohort: Total = 177	BMI > 95th %ile	Age range: 5-19 9.0 ± 1.5; F=61%	NR	D+E: Clinic (Hospital)	Behavioural intervention with individualised behavioural goals for nutrition, PA & family support.	5: 6	GP1: -0.18 GP2: -0.13 All: -0.15	GP1:-2.10 GP2:-2.40 All:-2.20
14	Klijn 2007[47] The Netherlands	Cohort: Total = 15	BMI>30	Age range:10-18 14.7(2.1); F=NR	NR	E: Community	Aerobic exercise training programme – 12 weeks; 3 x 30-60 min aerobic group sessions/week (2x gym/outdoors, 1 x swimming pool). P.E teacher led. Diverse indoor, outdoor and swimming activities.	3: 0	All: -0.4	All: -3.80
15	Lazzer 2008[48] Italy	Cohort: Total = 19 Boys = 7 Girls = 12	BMI > 97th %ile	Age range: 8-12 Boys: 9.9 ±1.6 Girls:11.2 ± 1.5 Overall F=63%	Yes – Tanner	D+E: Community	2 x 50min/wk endurance training + 2hr/wk PE lessons + 1 x wk child & parent dietetic class + 1 x wk psychological group class.	8: 12	Boys: -0.4 Girls: -0.2	Boys: -4.00 Girls:-2.20
16	Meyer 2006[49] Germany	RCT: Total = 67 IG=33 OC=34	BMI > 97th %ile (German paediatric population)	Age range: 11-16 IG: 13.7 ± 2.1; F=48% OC: 14.1 ± 2.4; F =50%	Yes - Tanner	E: Clinic (Hospital)	3 x exercise sessions (Monday: swimming and aqua aerobic training 60 min + Wednesday sports games 90 min + Friday walking 60 min)/ wk; Control: Maintain current level of PA	6: 0	IG: -0.43 CG: -0.14	IG:-1.00 CG: 0.00
17	Miraglia 2015[50]Brazil	Cohort: Total = 27	BMI z-score > 2	Age range: 6-13 Median 10.3; F=48%	NR	D+E: Clinic (Hospital)	AmO: Outpatient Ambulatory. Obesity outpatient clinic - lifestyle change based on goals agreed relative to feeding habits & physical exercise, followed mthly. 12 mths: Subjects assessed at inclusion & after 12 mths of FU to obtain anthropometric & adipokine measurements.	12: 0	All: -0.4	All: -0.10

For peer review only - http://bmjopen.bmj.com/site/about/guidelines.xhtml

18	Morell-Azanza 2016[51]<br>Rendo-Urteaga 2015[52]<br>Spain<br>**(same cohort)**	Cohort:<br>Total = 40<br>Cohort:<br>Total = 12<br>high responders =6<br>low responders = 6	OW/OB as per Cole *et al* 2000	Age range: 7-15<br>Mean =11<br>F=53%	Yes – Tanner	D:<br>Clinic (Hospital)	Moderate energy-restricted diet + nutritional education sessions with dietitian + family involvement.	2.5:<br>0	HR:-0.79<br>LR: -0.18<br><br>HR: -0.64<br>LR: -0.07	HR:-3.10<br>LR: -0.60<br><br>HR: -2.49<br>LR: -0.37
19	Murer 2011[53]<br>Aeberli, 2010[54]<br>Switzerland<br>**(same cohort)**	Cohort:<br>Total = 206 (197)	BMI > 98th %ile	Age range:10-18<br>14.1 ± 1.9; F=42%	NR	D+E:<br>Clinic/hospital	Moderate caloric restriction.2 x 60-90min/day endurance exercise + 4-5 hr/wk. exercise session + behaviour modification.	2:<br>0	All: -0.42	All: -5.50
20	Murdolo 2017[55]<br>Italy	Cohort:<br>Total = 53	NR	Age range: 5-13<br>Responders: 9.0 ± 1.1; F=50%<br>Non-responders: 2.09 ± 0.32; F=33%	Yes - Tanner	D+E:<br>Community	Educational Wt Excess Reduction Program	24:<br>>6 mths	Responders:<br>-0.44<br>Non-responders: 0.11	Responders:-2.90<br>Non-responders: -2.00
21	Ning 2014[56]<br>& BEAN 2011[57]<br>USA<br>**TEENS**<br>**(same cohort)**	Cohort:<br>Total = 145**	BMI ≥ 95th %ile (CDC)	Age range: 11-18<br>13.1<br>F=65%	NR	D+E:<br>Academic Institution	12 x 30 min nutritional session with adolescent and parent/s + Education/behavioural support sessions once every 2 wks, or alternating wks + PA 3 x 60 min/wk during initial 12 wks, then minimum of twice/wk.	6:<br>0	All: -0.1	All: -2.40
22	Pacifico 2013[58]<br>Italy	Cohort:<br>Total = 120	BMI > 95th %ile	Age range: (11.5-12.2)<br>11.9; F=35%	Yes (method ND)	D+E:<br>Clinic (Hospital)	Hypocaloric diet (25-30 Kcal/kg/day) + 60 min/day ~ 5 days/wk moderate exercise + Reduce sedentary behaviour.	12:<br>0	All: -0.32	All: -2.10
23	Racil 2013[59]<br>Tunisia	RCT:<br>Total = 34<br>HIT=11<br>MIIT=11<br>OC=12	BMI > 97th %ile (French standards)	Age range: NR<br>HIIT: 15.6 ± 0.7<br>MIIT: 16.3 ± 0.52<br>OC:15.9 ±1.2<br>Overall F=100%	Yes - Tanner	D+E:<br>Community	4-day diet records + HIIT or MIIT. Interval training program 3 x /wk on non-consecutive days.	3:<br>0	HIT: -0.4<br>MIT: -0.3<br>OC: 0	HIT: -2.90<br>MIT:-2.00<br>OC: -0.40
24	Racil 2016[60]<br>Tunisia	RCT:<br>Total = 47<br>HIIT =17<br>MIIT16<br>OC =14	BMI > 97th %ile (French standards)	Age range: NR<br>14.2 ± 1.2; F=100%	NR	E:<br>Academic Institution	HIIT (Warm up + Interval training at 100%/50% MAS + Cooling down);<br>MIIT (Warm up + Interval training 80%/50% MAS + Cooling down)	3:<br>0	HIT: -0.3<br>MIT: -0.3<br>OC: 0	HIT:- -3.90<br>MIT:-3.40<br>OC: -0.50
25	Reinehr 2004a[61]<br>Germany<br>**OBELDICKS**	Cohort:<br>Total = 42	BMI ≥ 97th %ile	Age range: 6.1-15.1<br>10.2; F=57%	Yes - Tanner	D+E:<br>Clinic (Hospital)	Obeldicks: Intensive phase 3 mths (Parents' course 2x/month + Behaviour therapy 2x/month + Nutritional course 2x/month + Exercise therapy 1x/wk) + Establishing phase 3 mths (Talk rounds for parents 1x/month + Psychological therapy + Exercise therapy 1x/wk) + Establishing phase 2 for 3 mths (Psychological therapy + Exercise therapy 1x/wk) + Establishing phase 3 for 3 mths (Exercise therapy 1x/wk).	12:<br>0	Sig. WL-0.9<br>NS WL: -0.2	Sig. WL:-7.50<br>NS WL:-3.00

For peer review only

For peer review only - http://bmjopen.bmj.com/site/about/guidelines.xhtml

26	Reinehr, 2008a, b[62;63]<br>Germany<br>**OBELDICKS**	Cohort:<br>RBP4<br>Total = 43 (+ n=19 lean)<br>Fetuin-A<br>Total = 34 (+ n=14 lean)	IOTF using pop. - specific data	Ob: 10.8 ± 2.6; F=61%<br>Lean C: 10.3±2.9; F=58%<br>Ob+NAFLD: 11.2 ± 2.8; F=61%<br>Ob + norm. wt; F=33%<br>NAFLD: 10.2 ± 2.0; F=67%<br>C: 10.3 ± 2.9, F=64%	Yes - Tanner	D+E:<br>Clinic (Hospital)	Obeldicks	12:<br>0	WL: -0.6<br>No WL: -0.1	WL: -8.00<br>No WL:0.00
27	Rohrer 2008[64]<br>Germany<br>**Fit Kids**	Cohort:<br>Total = 22	BMI > 99.5th %ile (German standard values) or BMI > 97th %ile with obesity-associated risk factors or BMI >90th %ile with obesity-associated disease	Age range: 7-15<br>Median: 11.9<br>F=27%	NR	D+E:<br>Community	Physical exercise (2 x wk, 100 hrs in total) + Nutritional/heath education and psychological care for the child (x wk, 43.5 hrs total) and parent/s (2 x wk, 12 hrs total).	12:<br>0	Increased BMI: 0.12<br>Reduced BMI:-0.35	Increased BMI: 1.05<br>Reduced BMI:-0.05
28	Rolland-Cachera 2004[65]<br>France	RCT:<br>Total = 99<br>PROT- = 61 (53)<br>PROT+ =60 (46)	BMI > 97th %ile (French reference values)	Age range: 11-16<br>PROT- = 14.1 ± 1.2; F = 74%<br>PROT + =14.4 ± 1.3; F = 72%	NR	D+E:<br>Academic Institution	Wt reducing diet; 7hr/wk vigorous sports + 7hr/wk outdoor activities; advice on nutrition & PA during wkends/holidays.	9:<br>12 + 24	PROT- :-2.6<br>PROT+:-2.5	PROT- :-12.40<br>PROT+:-12.10
29	Roth 2016[66]<br>Germany<br>**OBELDICKS**	Cohort:<br>Total = 69	OB as per IOTF criteria	NR – (see Obeldicks age range)<br>Ob with WL: 11.8 ± 2.0; F=50%<br>Ob without WL: 12.1 ± 2.1; F=51%<br>Normal wt: 12.3 ± 3.0; F=45%	Yes - Tanner	D+E:<br>Clinic (Hospital)	Obeldicks	12:<br>0	WL: -0.69<br>No WL: 0.03	WL: -9.60<br>No WL: -4.30
30	Savoye 2005[67]<br>USA<br>**Bright Bodies**	Cohort:<br>Total = 25 SMP=10<br>BFC = 23	BMI ≥ 95th %ile	Age range: 11-16<br>13.5 ± 0.3;<br>SMP:13.3 ±0.6; F=75%<br>BFC: 13.6 ±0.3; F= 65%	NR	D+E:<br>Academic Institution	Bright Bodies Weight Management Program: nutrition education, exercise, behavioural modification.<br>2 x 30 min exercise sessions + 1 x 45 min nutrition/behaviour medication group session per week. 4 levels: Beginner, Intermediate i, Intermediate ii, Advanced. All levels 12 weeks duration.<br>Mthly maintenance classes after 1 yr (support-group style)	12:<br>12	SMP: -0.36<br>BFC: -0.12	SMP:-6.50<br>BFC: -4.20
31	Savoye 2007, 2011[68;69]<br>USA<br>**Bright Bodies**<br>(data taken from 2011 paper)	RCT+ Long term FU results (cohort)<br>RCT Total = 174<br>BB=105<br>CC=69<br>FU Total = 76 at 24 mths	BMI ≥ 95th %ile (CDC)	Age range: 8-16<br>BB: 12.0 ± 2.5; F=55%<br>CC: 12.5 ± 2.3; F=68%<br><br>FU cohort:<br>13.9 ± 2.4; F=62%	NR	D+E:<br>Academic Institution (local school).	Bright Bodies Weight Management Program: nutrition education, exercise, behavioural modification.2 x sessions/wk for 6 mths, then biweekly for next 6 mths. BB: 2x50 min exercise + 1x40 min nutrition/behaviour modification per wk + 12 mths no active intervention.<br>Control group: standard care – paed. obesity clinic (biannual clinic appt; diet + exercise counselling) Structured tx & teaching program (28 x 45 min therapeutic sessions e.g. PA, nutrition, healthy cooking)	12:<br>12<br><br>FU<br>1.5:<br>24	IG: -0.21<br>CG: 0.01	IG: -3.90<br>CG: 2.10

For peer review only

For peer review only - http://bmjopen.bmj.com/site/about/guidelines.xhtml

32	Savoye 2014[70] USA **Bright Bodies**	RCT Total = 75 BB=38 (31) CC =37 (27)	BMI ≥ 95th %ile	Age range:10-16 BB: 12.7 (1.9); F=68% CC: 13.2 (1.8); F=62%	Yes- Tanner	D+E: Academic Institution	Bright Bodies Weight Management Program: nutrition education, exercise, behavioural modification. 2 x 30 min exercise sessions + 1 x 45 min nutrition/behaviour medication group session per week. 4 levels: Beginner, Intermediate I, Intermediate ii, Advanced. All levels 12 weeks duration. Mthly maintenance classes after 1 yr (support-group style)	6: 0	BB: -0.05 CC: 0.04	BB: -3.30 CC: 0.40
33	Schiel 2016[71] Germany	Cohort: Total =143	BMI-SDS ≥97th %ile	Age range: NR 13.9 ± 2.4; F=62%	NR	D+E: Clinic (Hospital)	Structured Tx & Teaching Program (STTP): 28 x 45 min therapeutic sessions e.g. PA, nutrition, healthy cooking	1.5: 24	All: -0.26	All: -3.40
34	Seabra 2016[72] Portugal	Cohort: Total = 88 soccer =29 Trad. Act. =29 OC =30	BMI-SDS > 2	Age range: 8-12 Soccer: 10.5 ± 1.5 Trad. act: 11.0 ± 1.6 OC=10.0 ±1.3 Overall F=0%	Yes - Tanner	E: Community	Soccer & trad. activity programmes (3 x 60-90min/wk) + 2 x 1hr at BL & 3 mths later energy balance session.	6: 0	Soccer: -0.2 Trad.: -0.2 CG: -0.1	Soccer:-2.20 Trad:-4.10 CG:3.10
35	Truby 2016[73] Australia	RCT: Total = 87 SMC =37 (33) SLF=36 (32) WList OC =14	BMI > 90th %ile (CDC)	Age range: 10-17 SMC: 13.2 ± 1.9; F=73% SLF: 13.2 ± 2.1; F=72% WList OC: 13.6 ±1.9; F=71%	Yes - Tanner	D: Clinic (Hospital)	Structured modified CHO diet (35% CHO; 30% protein; 35% fat), structured low-fat diet (55% CHO; 20% protein; 25% fat), Control (no dietary advice).	3: 0	SLF: -0.09 SMC:-0.15 CG: 0.02	SLF: -0.13 SMC: -0.40 CG: 2.62
36	Van der Baan-Slootweg 2014[74] Netherlands	RCT: Total = 90 Inpt. = 45 (37) AmO = 45 (36)	BMI z score ≥ 3.0 or > 2.3 with OB-related health problems	Age range: 8-18 Inpt: 13.8 ± 2.3; F=58% AmO: 13.9 ± 2.5; F=58%	NR	D+E: Clinic (Hospital)	Inpt. (Hospitalised 26 wks on working days - 4 days/wk 30-60min exercise + nutrition/BM once/wk + parents/caregivers 3 x 1hr lesson on nutrition/BM); Ambulatory (12 visits at increasing time intervals - 1 hr exercise session + encouraged 3 x exercise/wk + 1 hr educational programme + 30 min nutrition education).	6: 24	InpT: -0.6 AmO: -0.35	InP: -3.34 AmO:-7.87
37	Visuthranukul 2015[75] Thailand	RCT: Total = 70 (52) I =35(25) OC=35 (27)	ND. BL BMI z-score: I = 3.7 ±0.9 C = 3.6±1.6	Age range: 9-16 I = 11.9 ± 1.9; F=36% C = 12.0 ± 2.1; F=30%	Yes - Tanner	D: Clinic (Hospital)	I (Low GI diet + Energy restriction 1400-1500 kcal/day + Increased exercise); OC (Energy restriction 1200-1300 kcal/day + Low fat/high fibre diet + Increased exercise).	6: 0	IG:-0.3 CG: -0.3	IG:0.10 CG:0.10
38	Vitola 2009[76] USA	Cohort: Total = 8(7)	BMI ≥ 95th %ile	Age range: NR 15.3± 0.6; F=12.8%	Yes - Tanner	D+E: Clinic (Hospital)	Individual behavioural therapy sessions with psychologist. Parents involvement encouraged. Self-monitoring of PA & food intake. Gradual reduction of caloric intake to ≈1200-1500 kcal/day. Ongoing therapy - wt loss therapy repeated when 5% body wt lost & wt stable for at least 4 wks	NR	All: -0.3	All: -5.30
39	Wickham, 2009[77] & Evans, 2009[78] USA **TEENS (same cohort)**	Cohort: Total = 168 (64) *	BMI ≥ 95th %ile (CDC)	Age range: 11-18 13.4 ± 1.8; F=60% 13.9 ± 1.9; F=62%	NR	D+E: Academic Institution	Exercise 1 day/wk at facility + 2 additional exercise days at facility of ppts' choice + 30 min/wk nutrition education/behavioural support sessions.	6: 0	Completers : -0.07	Completers :-1.30

KEY: %ile = percentile; AmO = Outpatient Ambulatory; An. = analysed; apt. = appointment; BB =Bright Bodies; BFC = Better food choices; BL = baseline; BM = behaviour modification; BMI= body mass index; C = control; CG: control group; CBT = cognitive behavioural therapy; CDC = Centre for Disease Control; CG = control group; CHO = carbohydrate; D = diet; E = exercise; FBBT = family-based behavioural treatment; F = female; FU = follow up; GI = glycaemic index; GT = group therapy; HGI = high glycaemic index; hr = hour; HZ = heterozygous; HO = homozygous; ht = height; I = intervention; IG= intervention group; IOTF = International Obesity Task Force; Inpt. = inpatient; LGI = low glycaemic index; LMS= least-mean-squares; LS = long stay; min= minute; mth = month; MO = morbidly obese; norm. normal; n = number; NAFLD = Non-alcoholic fatty liver disease; ND = not described; NR = not reported; OB = obese; OC =

For peer review only - http://bmjopen.bmj.com/site/about/guidelines.xhtml

obese control; OW = overweight; paed. = paediatric; PA = physical activity; PE = physical activity; PROT= protein; RCT = randomised controlled trial; SD = standard deviation; SDS = standard deviation score; SMP= Structured meal plan; SS= short stay; SMC= structured modified carbohydrate diet; trad. = traditional; Trad. act = traditional activity; tx = treatment; wk = week; WList OC– wait list obese control; WL = weight loss; wt = weight; X-over = crossover; yr = year

*studies with change in % body fat included in the analysis
**minor discrepancies in reporting of data in papers

N.B. For studies reported in multiple publications, the reference that provided the most comprehensive information has been used *(thus Ning et al 2014[56] includes data from Bean et al 2011[57]; Evans et al 2009[78] is reported under Wickham et al 2009[77], Aeberli et al 2010[54] is reported under Murer et al 2011[53]; Rendo-Urtega et al 2015[52] is reported under Morell-Azanza et al[51] and Kazankov et al 2014[44] is reported under Grønbæk et al* 2012[43]).

For peer review only

Narrative description of studies that reported BMI-SDS and percentage body fat

Of the 39 studies that reported percentage body fat included in our analysis, seven were conducted in both Germany and USA, four in Italy, followed by Australia (n=2), Denmark (n=2), Netherlands (n=2), Poland (n=2), Switzerland (n=2), Tunisia (n=2) and one each in Belgium, Brazil, Canada, Chile, France, Portugal, Spain, Thailand and the UK. There were country-specific variations in the definition of obesity, with most studies defining obesity by participants having a BMI-SDS > 2, or a BMI percentile of at least > 90th percentile. Most of studies utilised a cohort design (n = 27), 11 were randomised controlled trials (RCTs), of which one included results from a cohort of the original RCT. There was also one study which adopted a quasi-randomised design.

Most studies (n=20) conducted their intervention in the hospital clinic setting. Eight studies conducted the intervention in the community setting and ten in academic institutions. One conducted the intervention in mixed setting, reporting use of both a community setting and academic institution.

Twenty-eight studies conducted interventions that comprised both diet and exercise components. The remaining studies (n=11) utilised interventions that focused either on exercise or diet only. The duration of the interventions ranged from 15 days to 24 months. The majority of studies (n=29; 74%) did not report any follow-up after the lifestyle treatment intervention. The duration of follow-up in the studies where it was conducted and reported, ranged from 6 months to 2 years.

The sample sizes of the included studies ranged from 8 to 203 participants. The age of the participants ranged from 4 to 19 years. Studies predominantly had a mix of males and females

For peer review only - http://bmjopen.bmj.com/site/about/guidelines.xhtml

(95%) with only three studies specifically focused on either only girls[59,60] or boys[72]. Seventeen studies (44%) measured pubertal development of participants according to Marshall and Tanner staging, with pubertal status categorised into three groups: prepubertal, pubertal, and late/post-pubertal[79]. Four studies (10%) reported that pubertal development was measured but the methodology was not defined. Eighteen studies (46%) did not report any measures of pubertal development.

Quality Assessment

The quality of the conduct of each study was assessed using the same criteria as the HTA systematic review of the long-term effects and economic consequences of treatments for obesity and implications for health improvement[30]. The results of the quality assessment can be found in Table 2. In summary, none of the 39 studies that reported percentage body fat were considered to be of poor quality, 21 studies (54%) were rated as being of moderate quality and 18 studies (46%) achieved a score over 81% indicating high quality.

For peer review only

For peer review only - http://bmjopen.bmj.com/site/about/guidelines.xhtml

Table 2: Quality Assessment of included studies

		SAMPLE						CONDUCT OF STUDY				FOLLOW-UP			ANALYSIS				INTERPRETATION				
	Author, Year,	1.Aims clearly stared	2. sample size justified	3. Age of participant defined	4. Measurements at start clearly stated?	5. Measurements likely to be valid and reliable?	6. Risk factors recorded clearly?	7. Was the intervention before follow -up defined?	8. Setting of the study clear?	9. Is mode of assessment described?	10. Did untoward events occur during the study?	11. Was there a follow up?	12. Was follow up necessary?	13. Are losses to follow up defined?	14. Was basic data adequately described?	15. Do numbers add up?	16. Did analysis allow for passage of time?	17. Was statistical significance assessed?	18. Were the main findings interpreted adequately?	19. Were null/negative findings interpreted?	20. Are important effects overlooked?	Total (x/40)	Overall rating
1	Bell 2007	Yes	Yes	Yes	Yes	Yes	No	Yes	?	Yes	No	No	Yes	Yes	Yes	Yes	Yes	Yes	Yes	Yes	No	**35**	**87.5**
2	Bock 2014	Yes	No	Yes	Yes	Yes	No	Yes	Yes	Yes	No	Yes	Yes	Yes	Yes	Yes	Yes	Yes	Yes	Yes	No	**36**	**90**
3	Bruyndonckz 2015	Yes	No	Yes	Yes	Yes	No	Yes	Yes	Yes	No	Yes	Yes	Yes	Yes	Yes	Yes	Yes	Yes	Yes	No	**36**	**90**
4	Bustos 2015	Yes	No	Yes	Yes	Yes	No	Yes	Yes	Yes	No	No	Yes	No	Yes	Yes	Yes	Yes	Yes	No	No	**30**	**75**
5	Calcaterra 2013	Yes	No	Yes	Yes	Yes	No	Yes	Yes	Yes	No	No	Yes	No	Yes	Yes	Yes	Yes	Yes	?	No	**31**	**77.5**
6	Dobe 2011	?	No	Yes	?	?	No	Yes	?	?	No	No	Yes	No	Yes	Yes	Yes	Yes	Yes	?	No	**26**	**65**
7	Farpour-Lambert, 2009	Yes	Yes	Yes	Yes	Yes	No	Yes	?	Yes	No	Yes	Yes	Yes	Yes	Yes	Yes	Yes	Yes	Yes	No	**37**	**92.5**
8	Ford, 2010a, b	Yes	Yes	Yes	Yes	Yes	No	Yes	Yes	Yes	?	Yes	Yes	Yes	Yes	Yes	Yes	Yes	Yes	Yes	?	**35**	**87.5**
9	Gajewska, 2016	Yes	No	Yes	Yes	Yes	No	Yes	Yes	Yes	No	No	Yes	No	Yes	Yes	Yes	Yes	Yes	?	No	**31**	**77.5**
10	Garanty-bogacka, 2011	Yes	No	Yes	Yes	Yes	?	?	?	Yes	?	No	?	No	Yes	?	Yes	Yes	Yes	?	No	**26**	**65**
13	Gronbaek 2012; Kazankov 2014	Yes	?	Yes	Yes	Yes	No	Yes	Yes	Yes	No	Yes	Yes	Yes	Yes	Yes	Yes	Yes	Yes	Yes	No	**37**	**92.5**
11	Hvidt 2014	Yes	No	Yes	Yes	Yes	No	Yes	Yes	Yes	No	Yes	Yes	Yes	Yes	Yes	Yes	Yes	Yes	Yes	?	**34**	**85**
12	Kirk, 2005	Yes	No	Yes	Yes	Yes	No	Yes	Yes	Yes	?	No	Yes	?	Yes	Yes	Yes	Yes	Yes	?	?	**29**	**72.5**
13	Klijn 2007	Yes	No	Yes	Yes	Yes	No	Yes	?	Yes	No	No	Yes	No	No	Yes	Yes	Yes	Yes	No	No	**27**	**67.5**
14	Lazzer 2008	Yes	No	Yes	Yes	Yes	No	Yes	Yes	Yes	No	Yes	Yes	No	Yes	Yes	Yes	Yes	Yes	No	No	**32**	**80**
15	Meyer 2006	Yes	No	Yes	Yes	Yes	No	No	Yes	Yes	No	No	Yes	No	Yes	Yes	Yes	Yes	Yes	Yes	No	**30**	**75**
16	Miraglia 2015	Yes	No	No	Yes	Yes	No	Yes	?	Yes	No	No	Yes	No	?	Yes	Yes	Yes	?	Yes	Yes	**25**	**62.5**
17	Morell-Azanza 2016; Rendo-Urteaga 2015	Yes	Yes	Yes	Yes	Yes	No	Yes	Yes	Yes	No	Yes	Yes	No	Yes	Yes	Yes	Yes	Yes	No	No	**32**	**80**

For peer review only - http://bmjopen.bmj.com/site/about/guidelines.xhtml

18	Murer 2011; Aeberli, 2010	Yes	Yes	Yes	Yes	Yes	No	Yes	Yes	Yes	No	Yes	Yes	Yes	Yes	Yes	Yes	Yes	Yes	Yes	No	**38**	**92**
19	Murdolo 2017	Yes	No	Yes	Yes	Yes	No	No	No	Yes	No	Yes	Yes	No	Yes	Yes	Yes	Yes	Yes	No	No	**28**	**70**
20	Ning 2014; Bean 2011	Yes	No	Yes	Yes	Yes	No	Yes	Yes	Yes	No	Yes	Yes	Yes	Yes	Yes	Yes	Yes	Yes	No	No	**34**	**85**
21	Pacifico 2013	Yes	No	Yes	Yes	Yes	No	Yes	Yes	Yes	No	Yes	Yes	No	Yes	Yes	Yes	Yes	Yes	No	?	**31**	**77.5**
22	Racil 2013	Yes	No	Yes	Yes	Yes	No	Yes	?	Yes	No	No	?	No	Yes	Yes	Yes	Yes	Yes	Yes	?	**29**	**72.5**
23	Racil 2016	Yes	No	?	Yes	Yes	No	Yes	?	Yes	No	No	Yes	No	Yes	Yes	Yes	Yes	Yes	No	No	**28**	**70**
24	Reinehr 2004a	Yes	No	Yes	Yes	Yes	No	Yes	Yes	Yes	No	No	Yes	No	Yes	Yes	Yes	Yes	Yes	No	?	**29**	**72.5**
25	Reinehr, 2008a, b	Yes	No	Yes	Yes	Yes	No	Yes	Yes	Yes	No	Yes	Yes	No	Yes	Yes	Yes	Yes	Yes	Yes	No	**32**	**80**
26	Rohrer 2008	Yes	No	Yes	Yes	Yes	No	Yes	Yes	Yes	No	Yes	Yes	No	Yes	Yes	Yes	Yes	Yes	?	No	**33**	**82.5**
27	Rolland-Cachera 2004	Yes	No	Yes	Yes	Yes	No	Yes	Yes	Yes	No	Yes	Yes	Yes	?	Yes	Yes	Yes	Yes	No	No	**33**	**82.5**
28	Roth 2016	Yes	No	Yes	Yes	Yes	No	Yes	No	Yes	No	No	Yes	No	Yes	Yes	Yes	Yes	Yes	No	No	**28**	**70**
29	Savoye 2005	Yes	No	Yes	Yes	Yes	No	Yes	Yes	Yes	No	Yes	Yes	Yes	Yes	Yes	Yes	Yes	Yes	No	No	**34**	**85**
30	Savoye 2007,2011	Yes	Yes	Yes	Yes	Yes	No	Yes	Yes	Yes	No	Yes	Yes	Yes	Yes	Yes	Yes	Yes	Yes	No	No	**36**	**90**
31	Savoye 2014	Yes	Yes	Yes	Yes	Yes	No	Yes	?	Yes	No	No	Yes	Yes	Yes	Yes	Yes	Yes	Yes	Yes	No	**35**	**87.5**
32	Schiel 2016	Yes	No	?	Yes	Yes	No	Yes	Yes	Yes	No	Yes	Yes	Yes	Yes	Yes	?	Yes	No	?	Yes	**29**	**72.5**
33	Seabra 2016	Yes	Yes	Yes	Yes	Yes	No	Yes	Yes	Yes	No	No	Yes	Yes	Yes	Yes	Yes	Yes	Yes	No	No	**34**	**85**
34	Truby 2016	Yes	Yes	Yes	Yes	Yes	No	Yes	Yes	Yes	No	Yes	Yes	Yes	Yes	Yes	Yes	Yes	Yes	Yes	No	**38**	**95**
35	Van der Baan-Slootweg 2014	Yes	Yes	Yes	Yes	Yes	No	Yes	Yes	Yes	No	Yes	Yes	Yes	Yes	Yes	Yes	Yes	Yes	No	No	**36**	**90**
36	Visuthranukul 2015	Yes	Yes	Yes	Yes	Yes	No	Yes	Yes	Yes	No	Yes	Yes	Yes	Yes	Yes	Yes	Yes	Yes	Yes	No	**38**	**95**
37	Vitola 2009	Yes	No	Yes	Yes	Yes	No	?	?	Yes	No	No	Yes	No	Yes	Yes	Yes	Yes	Yes	No	No	**28**	**70**
38	Wickham, 2009; Evans 2009	Yes	No	Yes	Yes	Yes	No	Yes	Yes	Yes	No	Yes	Yes	?	Yes	Yes	Yes	Yes	No	Yes	?	**30**	**75**

DECISION KEY:

? = Unclear

For Q6. Were risk factors clearly recorded? We said “No” rather than “unclear” to all the studies that didn’t record risk factors;

For Q10. Did untoward events occur during the study? We said “No” rather than unclear if not mentioned.

Rating: Not satisfactory 1-50%; Moderate quality = 51-80%; High quality = 81%

For peer review only - http://bmjopen.bmj.com/site/about/guidelines.xhtml

Quantitative Analysis

From the studies that reported percentage body fat, 67 data subsets were identified which documented both a mean change in BMI-SDS/z-score and an associated mean change in percentage body fat or gave pre- and post- 'intervention' values from which these could be calculated as well as the number of cases analysed. Note that 'intervention' here could have been the 'control' arm in some cases. The following studies were excluded from analysis for the following reasons: there were different numbers of participants for BMI-SDS and percentage body fat[90]; five were duplicated in other studies[52,54,57,69, 78]. One further study (3 data sets) was excluded because the standard errors (SEs) were estimated from a Mixed Model and not directly comparable[108]. These excluded studies are reported in Appendix 2.

SEs were required for the mean changes in percentage body fat and, if not given explicitly, were calculated, from either the standard deviations (SDs) or the 95% confidence intervals (CIs) of the mean changes. In total, 23 data sets had SEs. For the remainder, the SEs were estimated from the SDs associated with the baseline and the post-intervention percentage body fat values, making an assumption about the degree of correlation between them. The median and interquartile range (IQR) of the correlation coefficients estimated from the 9 data sets where both the SEs of mean change and the SDs for baseline and post intervention percentage body fat values were available was 0.81 (IQR 0.59-0.82) and 0.81 has been used in the following analysis. A small number of data sets (n=6)[34,41,61]only had medians and IQRs (or range) reported for the baseline and post intervention results; the mean and SDs were estimated from them[80].

The meta-regression line was fitted and plotted together with the 95% prediction intervals for the change in percentage body fat across the study data sets. The smallest reduction of mean BMI-SDS/z-score associated with a reduction in mean percentage body fat was determined as

For peer review only - http://bmjopen.bmj.com/site/about/guidelines.xhtml

the smallest reduction in mean BMI-SDS/z-score with an associated 95% prediction interval wholly below zero.

A series of sensitivity analyses were conducted and these are presented in Figure 5. Sensitivity Analysis 5i: using the 23 cases where the SEs of the mean change in percentage body fat were actually known, Sensitivity Analysis 5ii: omission of 2 extreme values and Sensitivity Analysis 5iii: assuming a correlation of 0.50 instead of 0.81. In further exploratory analyses, the percentage of girls and the length of the study (baseline to end of intervention) were added to see if these affected the prediction of mean change in percentage body fat.

Results from the quantitative analysis

Figure 3 shows the results of the analysis and the fitted regression line. The circles represent the study results (i.e. the mean changes in percentage body fat and mean changes in BMI-SDS/z-score) analysed for each study, with the size of the circles representing the precision of the mean change in percentage body fat, i.e. the reciprocal of the SE squared).

Figure 3: Regression line showing the relationship between mean change in percentage body fat and BMI-SDS/z-score across the 39 studies (67 subsets) analysed

The fitted regression line shown in Figure 3 is:

Mean change in percentage body fat = 5.18 x Mean change in BMI-SDS/z-score - 0.781.

The regression slope was statistically significant ($P<0.001$), confirming a relationship between the mean loss of percentage body fat and the mean change in BMI-SDS/z-score across the data subsets; the proportion of the between-subset variance explained by the mean change in BMI-SDS/z-score (i.e. 'a type of adjusted R-squared') was 68%. There was, however, significant

For peer review only - http://bmjopen.bmj.com/site/about/guidelines.xhtml

between-subset heterogeneity with 89% of the percentage of the total residual variance attributable to this, (i.e. I^2). It was further noted that when added to the model, neither the percentage girls in the study sets nor the durations of the interventions significantly improved the prediction of mean change in percentage body fat from the mean change in BMI-SDS/z-score (P=0.54, P=0.97 respectively).

Figure 3 also shows the 95% prediction intervals for the mean change in percentage body fat. The upper limit of the prediction interval was below 0 only when the mean reduction in BMI-SDS/z-score was greater than 0.6, suggesting that any new study should aim to reduce the BMI-SDS/s-score by at least this amount to be confident of achieving a mean reduction of percentage body fat.

A normal plot for the standardised predicted random effects is shown in Figure 4. Most were within +/-2 although the data sets themselves were not wholly independent (as some came from the same studies).

Figure 4: Normal plot to show the standardised predicted random effects from the meta-regression

None of the sensitivity analyses conducted (Figure 5) significantly altered the findings, namely that a mean change of 0.6 or more in BMI z-score was associated with a definitive mean loss in percentage body fat. In Figure 5(ii), with the exclusion of the two extreme data points, the linear trend can be seen more clearly across the range of mean BMI z-score losses.

Figure 5: Sensitivity analyses

For peer review only - http://bmjopen.bmj.com/site/about/guidelines.xhtml

DISCUSSION

Summary of main results

This is the first of a series of papers that report on studies identified in a large systematic review. The objective of this paper was to attempt to establish the minimum change in BMI-SDS/z-score needed to achieve improvements in body fat in obese children and adolescents; BMI-SDS/z-score being by far the most frequently reported outcome in terms of weight management trial interventions in childhood. Seventy-three of the 90 included studies reported adiposity measures but in our meta-regression only percentage body fat can be used as a reliable, comparable marker of change of adiposity. Thus, the analyses presented in this paper were conducted using data from 39 studies. All of the included studies were considered to be of moderate to high quality according to the HTA quality assessment tool[30]. Despite there being a positive relationship between mean change in percentage body fat and mean change in BMI-SDS, our modelling suggested that, in order to be confident of effecting a mean loss in percentage body fat, any future study should aim to reduce the BMI-SDS/z-score by at least 0.6.

Strengths and limitations

We believe that this is the first paper to attempt to bring together all studies that have reported both a change in BMI-SDS/z-score and changes in a marker of adiposity in the obese paediatric population. The systematic methods employed to identify the included studies were stringent, but it is possible that some relevant studies might have been missed. In addition, there was some variation in the reporting of results where there were multiple publications of the same study; in these cases, the results from the most comprehensive paper have been used. An important limitation to address in the broader context going forward, is whether BMI-SDS/Z-

For peer review only

For peer review only - http://bmjopen.bmj.com/site/about/guidelines.xhtml

scores are the best way to represent changes in BMI at extremes of body weight. This has been addressed in a recent article by Freedman et al demonstrating that there are better measures of adiposity in severe obesity, such as percentage of 95th percentile BMI (%BMI^{p95}) or distance in Kg/m^2 from the 95th percentile (Δ BMI^{p95}) [119]. However, we based this analysis on the data available to us which was almost entirely reported in terms of BMI-SDS/z-scores.

It has been suggested that the relationship between change in percentage body fat and change in BMI-SDS/z-score may differ between very young and older children[120]). Our inclusion criteria stipulated ages from 4 to 19 years. Most of the studies spanned a wide range of ages (see Table 1) and we did not have access to individual child data to facilitate stratification by age. Data from two studies based specifically on younger children[37,41] (4-8 years, and 5-10 years respectively), however, did not suggest a different relationship from the whole cohort (Appendix 3).

Agreements and disagreements with other research

Previous research has shown that an improvement in body composition and cardiometabolic risk can be achieved with a BMI-SDS reduction of greater than or equal to 0.25 in obese adolescents, with greater benefits achieved when losing at least 0.5 BMI-SDS[39].

In clinical practice, the degree of weight loss with lifestyle intervention is moderate and the success rate 2 years after onset of an intervention is low (<20% with a decrease in BMI-SDS <0.25)[81]. There have been numerous reports of lifestyle-based weight management interventions for obese children, many documenting changes in BMI-SDS/z-score but a recent meta-analysis has documented that whilst such changes may be statistically significant, they are unlikely to lead to clinical improvements in metabolic health[82, 83]. To our knowledge this is

For peer review only - http://bmjopen.bmj.com/site/about/guidelines.xhtml

the first paper to establish the minimum change in BMI-SDS/z-score required to be certain of improving adiposity as percentage body fat for obese children and adolescents in clinical trials.

Clinical implications

If reducing fat mass is the aim of weight management interventions, our analysis in this review demonstrates that BMI-SDS/z-score changes must be of an order seldom achieved in trials worldwide. From our model, to be confident about ensuring an improvement in mean body fat, one should aim to reduce mean BMI-SDS/z-score by at least 0.6. Figure 3 and Sensitivity Analysis 5ii (Figure 5) suggest that to reduce body fat by 5% requires a much larger BMI-SDS reduction, of the order of 1.3 to 1.5, although there was a paucity of data in this region.

Recommendations for future research

Whilst we are undertaking further analyses looking at key cardiovascular and metabolic outcomes in childhood obesity that may demonstrate improvements at lesser levels of BMI-SDS/z-score reduction, the evidence suggests that very few childhood weight management trials to date are likely to have improved percentage body fat and calls in to question their overall efficacy in terms of health improvement. That said, any trial demonstrating an improvement of the magnitude of 0.6 BMI-SDS might be termed successful with a likely reduction in fat mass.

Conclusions

Using our model, to predict any fat mass improvement when reporting a weight management trial outcome requires a BMI-SDS/z-score decrease of 0.6. When evaluating key outcomes for

For peer review only - http://bmjopen.bmj.com/site/about/guidelines.xhtml

future weight management trials and services, this figure needs to be borne in mind by researchers, health care professionals and commissioners when assessing apparent success. However, given the evidence that BMI-SDS/z-scores may not accurately reflect adiposity at extremes of obesity it seems prudent for future trials to report additional indices of derived BMI values which may better reflect changes in actual adiposity.

Author Contributions

Ms Birch and Dr Perry provided substantial contributions to the conception and design of the study, designed the data extraction instrument, performed electronic database searches, data screening, extraction and quality assessment, coordinated and supervised data collection and drafted and revised the manuscript.

Professor Hamilton-Shield provided a substantial contribution to the conception and design of the study, conducted data screening and interpretation, and assisted with drafting and revision of the manuscript.

Dr Hunt provided statistical expertise in relation to study design and conducted the data analyses and contributed to the drafting and revision of the manuscript.

Mr Matson, Ms Chong and Ms Beynon were involved in data acquisition and management.

All authors approved the final manuscript as submitted and agree to be accountable for all aspects of the work.

For peer review only

For peer review only - http://bmjopen.bmj.com/site/about/guidelines.xhtml

References

1. Organization, WHO, Report of the commission on ending childhood obesity. 2016: World Health Organization.

2. Poskitt E. Defining childhood obesity: the relative body mass index (BMI). *Acta Paediatrica.* 1995;84(8):961-963.

3. Cole TJ, Green PJ. Smoothing reference centile curves: the LMS method and penalized likelihood. *Statistics in Medicine.* 1992;11(10):1305-1319.

4. Simmonds M, Llewellyn A, Owen CG, Woolacott N. Predicting adult obesity from childhood obesity: a systematic review and meta-analysis. *Obesity Reviews.* 2016;17(2): 95-107.

5. Hannon TS, Rao G, Arslanian SA. Childhood Obesity and Type 2 Diabetes Mellitus. *Pediatrics.* 2005;116(2):473-480.

6. Haines L, Wan KC, Lynn R, Barrett TG, Shield JP. Rising Incidence of Type 2 Diabetes in Children in the U.K. *Diabetes Care*. 2007;30(5):1097-1101.

7. Urbina EM, Kimball TR, McCoy CE, et al. Youth with Obesity and Obesity-Related Type 2 Diabetes Mellitus Demonstrate Abnormalities in Carotid Structure and Function. *Circulation.* 2009;119(22):2913-2919.

8. Pires A, Martins P, Pereira AP, et al. Insulin Resistance, Dyslipidemia and Cardiovascular Changes in a Group of Obese Children. *Arquivos Brasileiros de Cardiologia.,* 2015;104:266-273.

9. Cook S, Kavey REW. Dyslipidemia and Pediatric Obesity. *Pediatric Clinics of North America* 2011;58(6):1363-1373.

10. Chiarelli F,Marcovecchio ML. Insulin resistance and obesity in childhood. *European Journal of Endocrinology.* 2008;159(suppl 1): S67-S74.

11. Tounian P, Aggoun Y, Dubern B, et al. Presence of increased stiffness of the common carotid artery and endothelial dysfunction in severely obese children: a prospective study. *The Lancet.* 2001;358(9291): 1400-1404.

12. Klein S, Burke LE, Bray GA, et al. Clinical Implications of Obesity with Specific Focus on Cardiovascular Disease. A Statement for Professionals from the American Heart Association Council on Nutrition, Physical Activity, and Metabolism: Endorsed by the American College of Cardiology Foundation. *Circulation.* 2004;110(18):2952-2967.

13. Krebs JD, Evans S, Cooney L, et al. Changes in risk factors for cardiovascular disease with body fat loss in obese women. *Diabetes, Obesity and Metabolism.* 2002;4(6):379-387.

14. Goldstein DJ. Beneficial health effects of modest weight loss. *International journal of obesity and related metabolic disorders.* 1992;16(6):397-415.

For peer review only - http://bmjopen.bmj.com/site/about/guidelines.xhtml

15. Colditz GA, Willett WC, Rotnitzky A, Manson JE. Weight gain as a risk factor for clinical diabetes mellitus in women. *Annals of Internal Medicine.* 1995;122(7):481-486.

16. National Heart, Lung, and Blood Institute. "Clinical guidelines on the identification, evaluation, and treatment of overweight and obesity in adults. Bethesda (MD): National Heart." *Lung, and Blood Institute* (1998).

17. Cole TJ. The LMS method for constructing normalized growth standards. *European Journal of Clinical Nutrition.* 1990; 44:45–60.

18. Ho M, Garnett SP, Baur L, et al. Effectiveness of Lifestyle Interventions in Child Obesity: Systematic Review with a Meta-analysis. *Pediatrics.* 2012;130(6): e1647-1671.

19. Colquitt JL, Loveman E, O'Malley C, et al. Diet, physical activity, and behavioural interventions for the treatment of overweight or obesity in preschool children up to the age of 6 years. *Cochrane Database Syst Rev*, 2016. **3**: p. Cd012105.

20. Oude Luttikhuis H, Baur L, Jansen H, et al., Cochrane review: Interventions for treating obesity in children. *Evidence-based child health: A Cochrane Review Journal.* 2009;4(4):1571-1729.

21. National Institute for Health and Care Excellence. Quality Standard [QS94]. Obesity in children and young people: prevention and lifestyle weight management programmes. *NICE*. 2015; https://www.nice.org.uk/guidance/qs94.

22. Friedemann C, Mahtani K, Thompson M, Perera R. Cardiovascular disease risk in healthy children and its association with body mass index: systematic review and meta-analysis. *British Medical Journal.* 2012; 345: e4759.

23. Prentice AM, Jebb SA. Beyond body mass index. *Obesity Review*. 2001; 2(3):141-7.

24. Cole TJ, Freeman JV, Preece MA. Body mass index reference curves for the UK, 1990. *Archives of Disease in Childhood*. 1995;73(1):25-29.

25. Kuczmarski RJ, Ogden CL, Grummer-Strawn LM, et al. CDC growth charts: United States. Advance Data, 2000;(314):1-27.

26. Cole TJ, Bellizzi MC, Flegal KM, Dietz WH. Establishing a standard definition for child overweight and obesity worldwide: international survey. *British Medical Journal*. 2000; 320(7244):1240.

27. De Onis M, Garza C, Onyango AW, Borghi E. Comparison of the WHO child growth standards and the CDC 2000 growth charts. *The Journal of Nutrition*. 2007;137(1):144-148.

For peer review only - http://bmjopen.bmj.com/site/about/guidelines.xhtml

28. De Onis MD, Onyango AW, Borghi E, Siyam A, Nishida C, Siekmann J. Development of a WHO growth reference for school-aged children and adolescents. Bulletin of the World Health Organization. 2007b; 85(9):660-667.

29. Higgins J, Green S. Cochrane handbook for systematic reviews of interventions. Chichester: Wiley; 2011.

30. Avenell A, Broom J, Brown TJ. Systematic review of the long-term effects and economic consequences of treatments for obesity and implications for health improvement. *Health Technol. Assess.* 2004; 8(21):iii-iv:1-182.

31. Harbord RM, Higgins, JPT. 'Meta-regression in Stata.' *The Stata Journal.* 2008;8(4):493-519.

32. Bell L, Watts K, Siafarikas A, et al. Exercise alone reduces insulin resistance in obese children independently of changes in body composition. *Journal of Clinical Endocrinol. Metab.* 2007; 92(11):4230-4235.

33. Bock DE, Watts K, Siafarikas A, et al. The Health Initiative Program for Kids (HIP Kids): effects of a 1-year multidisciplinary lifestyle intervention on adiposity and quality of life in obese children and adolescents - a longitudinal pilot intervention study. *BMC Pediatrics.* 2014; 14:296. doi: 10.1186/s12887-014-0296-1

34. Bruyndonckx L, Hoymans VY, De Guchtenaere A, et al. Diet, exercise, and endothelial function in obese adolescents. *Pediatrics.* 2015;135(3): e653-e661.

35. Bustos P, Orias J, Sáez K, Maldonado M, Cuadra L, Asenjo S. Effects of the Bright Bodies Program in Chilean obese children. *Revista Medica De Chile.* 2015;143(9):1136-1143.

36. Calcaterra V, Larizza D, Codrons E, et al. Improved metabolic and cardiorespiratory fitness during a recreational training program in obese children. *Journal of Pediatric Endocrinology.* 2013; 26(3-4):271-276.

37. Dobe M, Geisler A, Hoffmann D, et al. [The Obeldicks concept. An example for a successful outpatient lifestyle intervention for overweight or obese children and adolescents]. *Bundesgesundheitsblatt, Gesundheitsforschung, Gesundheitsschutz*. 2011; 54(5):628-635.

38. Farpour-Lambert NJ, Aggoun Y, Marchand LM, Martin XE, Herrmann FR, Beghetti M. Physical activity reduces systemic blood pressure and improves early markers of atherosclerosis in pre-pubertal obese children. *Journal of the American College of Cardiology*. 2009; 54: 2396-406 DOI: 10.1016/j.jacc.2009.08.030.

39. Ford AL, Hunt LP, Cooper A, Shield JP. What reduction in BMI SDS is required in obese adolescents to improve body composition and cardiometabolic health? Archives of Disease in Childhood. 2010a; 95;256-261. DOI: 10.1136/adc.2009.165340.

For peer review only

For peer review only - http://bmjopen.bmj.com/site/about/guidelines.xhtml

40. Ford AL, Bergh C, Sabin MA, Hollinghurst S, Hunt LP, Shield JP. Treatment of childhood obesity by retraining eating behaviour: randomised controlled trial. *British Medical Journal,* 2010b. 340: p. b5388.

41. Gajewska J, Kuryłowicz A, Mierzejewska E, et al. Complementary Effects of Genetic Variations in LEPR on Body Composition and Soluble Leptin Receptor Concentration after 3-Month Lifestyle Intervention in Prepubertal Obese Children. *Nutrients.* 2016;8(6). pii: E328. doi: 10.3390/nu8060328

42. Garanty-Bogacka B, Syrenicz M, Goral J, et al. Changes in inflammatory biomarkers after successful lifestyle intervention in obese children. *Endokrynologia Polska.* 2011;62(6):499-505.

43. Grønbæk H, Lange A, Birkebæk NH, et al. Effect of a 10-week weight loss camp on fatty liver disease and insulin sensitivity in obese Danish children. *Journal of Pediatric Gastroenterol.Nutr*. 2012 54(2) p.223-228.

44. Kazankov K, Møller HJ, Lange A, et al. The macrophage activation marker sCD163 is associated with changes in NAFLD and metabolic profile during lifestyle intervention in obese children. *Pediatric Obesity*. 2015;10(3):226-233.

45. Hvidt KN, Olsen MH, Ibsen H, Holm JC. Effect of changes in BMI and waist circumference on ambulatory blood pressure in obese children and adolescents. *Journal of Hypertension.* 2014; 32(7):1470-1477.

46. Kirk S, Zeller M, Claytor R, Santangelo M, Khoury PR, Daniels SR. The relationship of health outcomes to improvement in BMI in children and adolescents. *Obesity Research*. 2005; 13(5): 876-882.

47. Klijn P, van der Baan-Slootweg O, van Stel HF. Aerobic exercise in adolescents with obesity: preliminary evaluation of a modular training program and the modified shuttle. BMC Pediatrics. 2007; 7(19): doi:10.1186/1471-2431-7-19

48. Lazzer S, Molin M, Stramare D, Facchini S, Francescato MP. Effects of an eight-month weight-control program on body composition and lipid oxidation rate during exercise in obese children. *Journal of Endocrinological Investigation.* 2008; 31(6): 509-514.

49. Meyer AA, Kundt G, Lenschow U, Schuff-Werner P, Kienast W. Improvement of early vascular changes and cardiovascular risk factors in obese children after a six-month exercise program. *Journal of the American College of Cardiology*. 2006; 48(9):1865-1870.

50. Miraglia F, de Moraes Silveira CR, Gomes Beghetto M, dos Santos Oliveira F, de Mello ED. Behavior of adipokines after a year follow-up in the Obesity Outpatient Clinic for Children and Adolescents. *Nutricion Hospitalaria*. 2015; 32(4):1554-1559.

51. Morell-Azanza L, García-Calzón S, Rendo-Urteaga T, et al. Serum oxidized low-density lipoprotein levels are related to cardiometabolic risk and decreased after a weight loss treatment in obese children and adolescents. *Pediatric Diabetes.* 2017; 18: 392-398.

For peer review only

For peer review only - http://bmjopen.bmj.com/site/about/guidelines.xhtml

52. Rendo-Urteaga T, García-Calzón S, González-Muniesa P, et al Peripheral blood mononuclear cell gene expression profile in obese boys who followed a moderate energy-restricted diet: Differences between high and low responders at baseline and after the intervention. *British Journal of Nutrition*. 2015; 113(2): 331-342.

53. Murer SB, Knöpfli BH, Aeberli I, et al. Baseline leptin and leptin reduction predict improvements in metabolic variables and long-term fat loss in obese children and adolescents: A prospective study of an inpatient weight-loss program. *American Journal of Clinical Nutrition*. 2011; 93(4): 695-702.

54. Aeberli I, Jung A, Murer SB, et al. During rapid weight loss in obese children, reductions in TSH predict improvements in insulin sensitivity independent of changes in body weight or fat. *The Journal of clinical endocrinology and metabolism*. 2010; 95(12):5412-8.

55. Murdolo G, Tortoioli C, Celi F, et al. Fetuin-A, adiposity-linked insulin resistance and responsiveness to an educational-based weight excess reduction program: a population-based survey in prepubertal schoolchildren. *Endocrine*. 2017; 56(2): 357-365.

56. Ning Y, Yang S, Evans RK, et al. Changes in body anthropometry and composition in obese adolescents in a lifestyle intervention program. *European Journal of Nutrition*. 2014; 53(4):1093-1102.

57. Bean M, Mazzeo SE, Stern M, et al. Six-month dietary changes in ethnically diverse, obese adolescents participating in a multidisciplinary weight management program. *Clin Pediatr*. 2011;50(5):408-416.

58. Pacifico L, Arca M, Anania C, Cantisani V, Di Martino M, Chiesa C. Arterial function and structure after a 1-year lifestyle intervention in children with nonalcoholic fatty liver disease. *Nutrition, metabolism, and cardiovascular diseases*. 2013; 23(10):1010-6.

59. Racil G, Ben Ounis O, Hammouda O, et al. Effects of high vs. moderate exercise intensity during interval training on lipids and adiponectin levels in obese young females. *European Journal of Applied Physiology*. 2013; 113(10):2531-40.

60. Racil G, Coquart JB, Elmontassar W, et al. Greater effects of high- compared with moderate-intensity interval training on cardio-metabolic variables, blood leptin concentration and ratings of perceived exertion in obese adolescent females. *Biology of Sport*. 2016; 33(2): 145-152.

61. Reinehr T, Roth C, Menke T, Andler W. Adiponectin before and after weight loss in obese children. *The Journal of Clinical Endocrinology and Metabolism*. 2004; 89(8): 3790-4.

62. Reinehr T, Stoffel-Wagner B, Roth CL. Retinol-binding protein 4 and its relation to insulin resistance in obese children before and after weight loss. *The Journal of Clinical Endocrinology and Metabolism*. 2008; 93(6):2287-93.

63. Reinehr T, Roth CL. Fetuin-A and its relation to metabolic syndrome and fatty liver disease in obese children before and after weight loss. *The Journal of Clinical Endocrinology and Metabolism*. 2008; 93(11):4479-85.

For peer review only - http://bmjopen.bmj.com/site/about/guidelines.xhtml

64. Rohrer TR, Rizzo F, Cäsar JJ, Muelbredt O, et al. Changes in Hepatic Risk Factors, Metabolic Variables, Body Composition, and Physical Fitness in Obese Children After a One-Year Weight Loss Program. *Journal of Pediatric Endocrinology & Metabolism,* 2008; 21(9): 837-845.

65. Rolland-Cachera MF, Thibault H, Souberbielle JC, et al. Massive obesity in adolescents: dietary interventions and behaviours associated with weight regain at 2 y follow-up. *International journal of obesity and related metabolic disorders*. 2004; 28(4):514-519.

66. Roth, CL, Elfers C, Lass N, Reinehr T. Betatrophin: No relation to glucose metabolism or weight status in obese children before and after lifestyle intervention. *Pediatric Diabetes.* 2017; 18: 485-491.

67. Savoye M, Berry D, Dziura J, et al. Anthropometric and psychosocial changes in obese adolescents enrolled in a Weight Management Program. *Journal of the American Dietetic Association*. 2005; 105(3):364-370.

68. Savoye M, Nowicka P, Shaw M, et al. Long-term Results of an Obesity Program in an Ethnically Diverse Pediatric Population. *Pediatrics.* 2011; 127(3):402-410.

69. Savoye M, Shaw M, Dziura J, et al. Effects of a Weight Management Program on Body Composition and Metabolic Parameters in Overweight Children. *JAMA*. 2007; 297(24):2697-2704.

70. Savoye M, Caprio S, Dziura J, et al. Reversal of early abnormalities in glucose metabolism in obese youth: Results of an intensive lifestyle randomized controlled trial. *Diabetes Care*. 2014;37(2):317 – 324.

71. Schiel R, Kaps A, Günter Stein G, Steveling A. Identification of Predictors for Weight Reduction in Children and Adolescents with Overweight and Obesity (IDA-Insel Survey). *Healthcare*. 2016; 4(1): 07. doi:10.3390/healthcare4010005

72. Seabra A, Katzmarzyk P, Carvalho MJ, et al. Effects of 6-month soccer and traditional physical activity programmes on body composition, cardiometabolic risk factors, inflammatory, oxidative stress markers and cardiorespiratory fitness in obese boys. *Journal of Sports Sciences*. 2016; 34(19):1822-1829.

73. Truby H. Baxter K, Ware RS, et al. A Randomized Controlled Trial of Two Different Macronutrient Profiles on Weight,Body Composition and Metabolic Parameters in Obese Adolescents Seeking Weight Loss. *PLoS ONE [Electronic Resource]*. 2016; 11(3): e0151787.

74. van der Baan–Slootweg O, Benninga MA, Beelen A, van der Palen J, et al. Inpatient treatment of children and adolescents with severe obesity in the Netherlands. A Randomized Clinical Trial. *JAMA Pediatr,* 2014; 168(9):807-814.

75. Visuthranukul C, Sirimongkol P, Prachansuwan A, Pruksananonda C, Chomtho S. Low-glycemic index diet may improve insulin sensitivity in obese children. *Pediatric Research*. 2015;78(5):567-573.

For peer review only - http://bmjopen.bmj.com/site/about/guidelines.xhtml

76. Vitola BE, Deivanayagam S, Stein RI, et al. Weight loss reduces liver fat and improves hepatic and skeletal muscle insulin sensitivity in obese adolescents. *Obesity*. 2009; 17:1744-1748.

77. Wickham EP, Stern M, Evans RK, et al. Prevalence of the Metabolic Syndrome Among Obese Adolescents Enrolled in a Multidisciplinary Weight Management Program: Clinical Correlates and Response to Treatment. *Metabolic Syndrome and Related Disorders*. 2009; 7(3):179-186.

78. Evans RK, Franco RL, Stern M, et al. Evaluation of a 6-month multi-disciplinary healthy weight management program targeting urban, overweight adolescents: Effects on physical fitness, physical activity, and blood lipid profiles. *International Journal of Pediatric Obesity*. 2009; 4(3):130-133.

79. Tanner JM. Growth at adolescence: with a general consideration of the effects of hereditary and environmental factors upon growth and maturation from birth to maturity. 2nd ed. Oxford, England: Blackwell Scientific, 1962.

80. Wan X, Wang W, Liu J, Tong T. Estimating the sample size and standard deviation from the sample size, median, range and/or interquartile range. *BMC Medical Research Methodology*. 2014; 14:135 doi: 10.1186/1471-2288-14-135.

81. Reinehr T. Lifestyle intervention in childhood obesity: changes and challenges. *Nat Rev Endocrinol*. 2013; 9(10):607-614.

82. Al-Khudairy L, Loveman E, Colquitt JL, et al. Diet, physical activity and behavioural interventions for the treatment of overweight or obese adolescents aged 12 to 17 years. *Cochrane Database Syst Rev*. 2017 Jun 22;6:CD012691. doi: 10.1002/14651858.CD012691.

83. Mead E, Brown T, Rees K, et al. Diet, physical activity and behavioural interventions for the treatment of overweight or obese children from the age of 6 to 11 years. *Cochrane Database Syst Rev*. 2017 Jun 22;6:CD012651. doi: 10.1002/14651858.CD012651.

84. Carraway ME, Lutes LD, Crawford Y, et al.. Camp-based immersion treatment for obese, low socioeconomic status, multi-ethnic adolescents. *Childhood obesity* (Print). 2014;10(2): 122-131.

85. Croker H, Viner RM, Nicholls D. Family-based behavioural treatment of childhood obesity in a UK National Health Service setting: randomized controlled trial. *International Journal of Obesity*. 2012; 36(1):16-26.

86. Doughty KN, Njike VY, Katz DL. Effects of a Cognitive-Behavioral Therapy-Based Immersion Obesity Treatment Program for Adolescents on Weight, Fitness, and Cardiovascular Risk Factors: A Pilot Study. *Childhood Obesity*. 2015;11(2):215-218.

For peer review only

For peer review only - http://bmjopen.bmj.com/site/about/guidelines.xhtml

87. Elloumi M, Ben Ounis O, Makni E, Van Praagh E, Tabka Z, Lac G. Effect of individualized weight-loss programmes on adiponectin, leptin and resistin levels in obese adolescent boys. *Acta Paediatr.* 2009; 98(9):1487-93.

88. Grulich-Henn J, Lichtenstein S, Hörster F, Hoffmann GF, Nawroth PP, Hamann A. Moderate weight reduction in an outpatient obesity intervention program significantly reduces insulin resistance and risk factors for cardiovascular disease in severely obese adolescents. *Int J Endocrinol.* 2011;2011:541021

89. Gunnarsdottir T, Einarsson SM, Njardvik U, et al. Family-based behavioral treatment for obese children - results and two year follow up. *Laknabladid*. 2014;100(3):139-45.

90. Holm JC, Buckert M, Hans-Christoph Friederich H-C, et al. Time course and determinants of leptin decline during weight loss in obese boys and girls. *International Journal of Pediatric Obesity*. 2007; 2(1): 2-10.

91. Kalavainen M, Utriainen P, Vanninen E, Korppi M, Nuutinen O. Impact of childhood obesity treatment on body composition and metabolic profile. *World Journal of Pediatrics.* 2012; 8(1):31-7.

92. Kolsgaard ML, Joner G, Brunborg C, et al. Reduction in BMI z-score and improvement in cardiometabolic risk factors in obese children and adolescents. The Oslo Adiposity Intervention Study - a hospital/public health nurse combined treatment.[*Erratum appears in BMC Pediatr. 2012;12:77; PMID: 22709339]*. BMC *Pediatrics.* 2011; 11: 47.

93. Kolotourou M, Radley D, Gammon C, Smith L, Chadwick P, Sacher PM. Long-Term Outcomes following the MEND 7-13 Child Weight Management Program*. Childhood Obesity.* 2015;11(3): 325-330.

94. Marcano, H, Fernández M, Paoli M, et al. Limited Weight Loss or Simply No Weight Gain following Lifestyle-Only Intervention Tends to Redistribute Body Fat, to Decrease Lipid Concentrations, and to Improve Parameters of Insulin Sensitivity in Obese Children. *International Journal of Pediatric Endocrinology.* 2011. 2011(1): 241703.

95. Mager DR, Iñiguez IR, Gilmour S, Yap J. The effect of a low fructose and low glycemic index/load (FRAGILE) dietary intervention on indices of liver function, cardiometabolic risk factors, and body composition in children and adolescents with non-alcoholic fatty liver disease (NAFLD). *J Parenter Enteral Nutr.* 2015;39(1):73-84.

96. Makkes S, Renders CM, Bosmans JE, van der Baan-Slootweg OH, Hoekstra T, Seidell JC. One-year effects of two intensive inpatient treatments for severely obese children and adolescents. *BMC Pediatrics,* 2016;16:120 doi: 10.1186/s12887-016-0659-x.

97. Martos R, Valle M, Morales RM, Cañete R, Gascón F, Urbano MM. Changes in body mass index are associated with changes in inflammatory and endothelial dysfunction biomarkers in obese prepubertal children after 9 months of body mass index SD score loss. *Metabolism-Clinical and Experimental.* 2009; 58(8):1153-1160.

For peer review only - http://bmjopen.bmj.com/site/about/guidelines.xhtml

98. Obert P, Gueugnon C, Nottin S, et al., Impact of diet and exercise training-induced weight loss on myocardial mechanics in severely obese adolescents. *Obesity*, 2013; 21(10): 2091-8.

99. Panagiotopoulos C, Ronsley R, Al-Dubayee M, et al., The Centre for Healthy Weights-Shapedown BC: A Family-Centered, Multidisciplinary Program that Reduces Weight Gain in Obese Children over the Short-Term. *International Journal of Environmental Research and Public Health*. 2011; 8(12):4662-4678.

100. Pozzato C, Verduci E, Scaglioni S, Liver Fat Change in Obese Children After a 1-year Nutrition-behavior Intervention. *Journal of Pediatric Gastroenterology and Nutrition.* 2010;51(3), 331–335.

101. Verduci E, Radaelli G, Salvioni M, Riva E, Giovannini M. Plasma long-chain fatty acids profile and metabolic outcomes in normolipidaemic obese children after one-year nutritional intervention. *Acta paediatrica*. 2011;100(4): 585-9

102. Reinehr T. and Andler W. Changes in the atherogenic risk factor profile according to degree of weight loss. *Archives of Disease in Childhood.* 2004;89(5):419-422.

103. Reinehr T, C Schmidt C, Toschke AM, Andler W. Lifestyle intervention in obese children with non-alcoholic fatty liver disease: 2-year follow-up study. *Archives of Disease in Childhood.* 2009; 94(6):437-442.

104. Rijks JM, Plat J, Mensink RP, Dorenbos E, Buurman WA, Vreugdenhil AC. Children with morbid obesity benefit equally as children with overweight and obesity from an ongoing care program. *Journal of Endocrinol Metab.* 2015; 100(9): 3572-3580.

105. Rovira AF, París Miró N, Zaragoza-Jordana M, et al Clinical and metabolic effectiveness of a new motivational therapy for the treatment of obesity in adolescents (OBEMAT). *Anales De Pediatria.* 2013. 78(3): 157-166.

106. Santomauro M, Paoli-Valeri M, Fernández M, et al., Non-alcoholic fatty liver disease and its association with clinical and biochemical variables in obese children and adolescents: Effect of a one-year intervention on lifestyle. *Endocrinología y Nutrición* (English Edition), 2012;59(6): p. 346-353.

107. Schum J, Blumenstock G, Weber K, et al. Variants of the FTO Gene in Obese Children and their Impact on Boy Composition and Metabolism before and after Lifestyle Intervention. *Experimental and Clinical Endocrinology & Diabetes*. 2012;120(3):128-131.

108. Shalitin S, Ashkenazi-Hoffnung L, Yackobovitch-Gavan M, et al. Effects of a Twelve-week randomized intervention of exercise and/or diet on weight loss and weight maintenance, and other metabolic parameters in obese preadolescent children. *Hormone Research*. 2009;72(5):287-301

109. Springer F, Ballweg V, Schweizer R, et al. Changes in whole-body fat distribution, intrahepatic lipids, and insulin resistance of obese adolescents during a low-level lifestyle intervention. *European Journal of Pediatrics,* 2015;174(12):1603-12.

For peer review only - http://bmjopen.bmj.com/site/about/guidelines.xhtml

110. Tan-Ting AM, Llido L. Outcome of a hospital based multidisciplinary weight loss program in obese Filipino children. *Nutrition*. 2011; 27:50-54.

111. Valle Jimenez M, Martos R, Morales RM, et al. Relationship between changes in plasma leptin concentrations and plasminogen activator inhibitor-1 in obese prepubertal children after nine months of treatment. *Annals of nutrition & metabolism.* 2013; 63(3): 216-22.

112. Vanhelst J, Fardy PS, Béghin L, Bui-Xuan G, Mikulovic J. Strategies in intervention programmes for obese youth: implication of the age and the type of physical activities. *Clinical Physiology and Functional Imaging*. 2015; 35(1): 17-20.

113. Vasquez F, Diaz E, Lera L, Vasquez L, Anziani A, Burrows R. Impact of a strength training exercise program on body composition and cardiovascular risk factors in a group of obese schoolchildren by pubertal stage. *American Journal of Sports Science and Medicine.* 2014; 2(1): 40-47.

114. Verduci E, Lassandro C, Giacchero R, Miniello VL, Banderali G, Radaelli G. Change in metabolic profile after 1-year nutritional-behavioral intervention in obese children. *Nutrients*. 2015; 7(12): 10089-10099.

115. Vos RC, Wit JM, Pijl H, Houdijk EC. Long-term effect of lifestyle intervention on adiposity, metabolic parameters, inflammation and physical fitness in obese children: a randomized controlled trial. *Nutrition & Diabetes*. 2011; 1:e9. doi: 10.1038/nutd.2011.5

116. Weiss R, Shaw M, Savoye M, Caprio S. Obesity dynamics and cardiovascular risk factor stability in obese adolescents. *Pediatric Diabetes.* 2009; 10(6): 360-7.

117. Wiegal C, Kokocinski K, Lederer P, Dötsch J, Rascher W, Knerr I. Childhood obesity: Concept, feasibility, and interim results of a local group-based, long-term treatment program. *J Nutr Educ Behav.* 2008; 40:369-373.

118. Wong WW, Abrams SH, Mikhail C, et al. An innovative summer camp program improves weight and self-esteem in obese children. *Journal of Pediatric Gastroenterology and Nutrition*. 2009; 49(4):493-497.

119. Freedman DS, Butte NF, Tavares EM, Lundeen EA, Blanck, HM, Goodman AB, Ogden CL. BMI z-scores are a poor indicator of adiposity among 2- to 19-year -olds with very high BMIs, NHANES 1999-2000 to 2013-2014. *Obesity,* 2017; 25(4): 739–746. doi:10.1002/oby.21782.

120. Woo JG, Cole TJ. Assessing adiposity using BMI-Z scores in children with severe obesity. *Obesity*, 2017; 25(4); 662

For peer review only

Figure 1: Flow diagram from the systematic review that identified the included studies

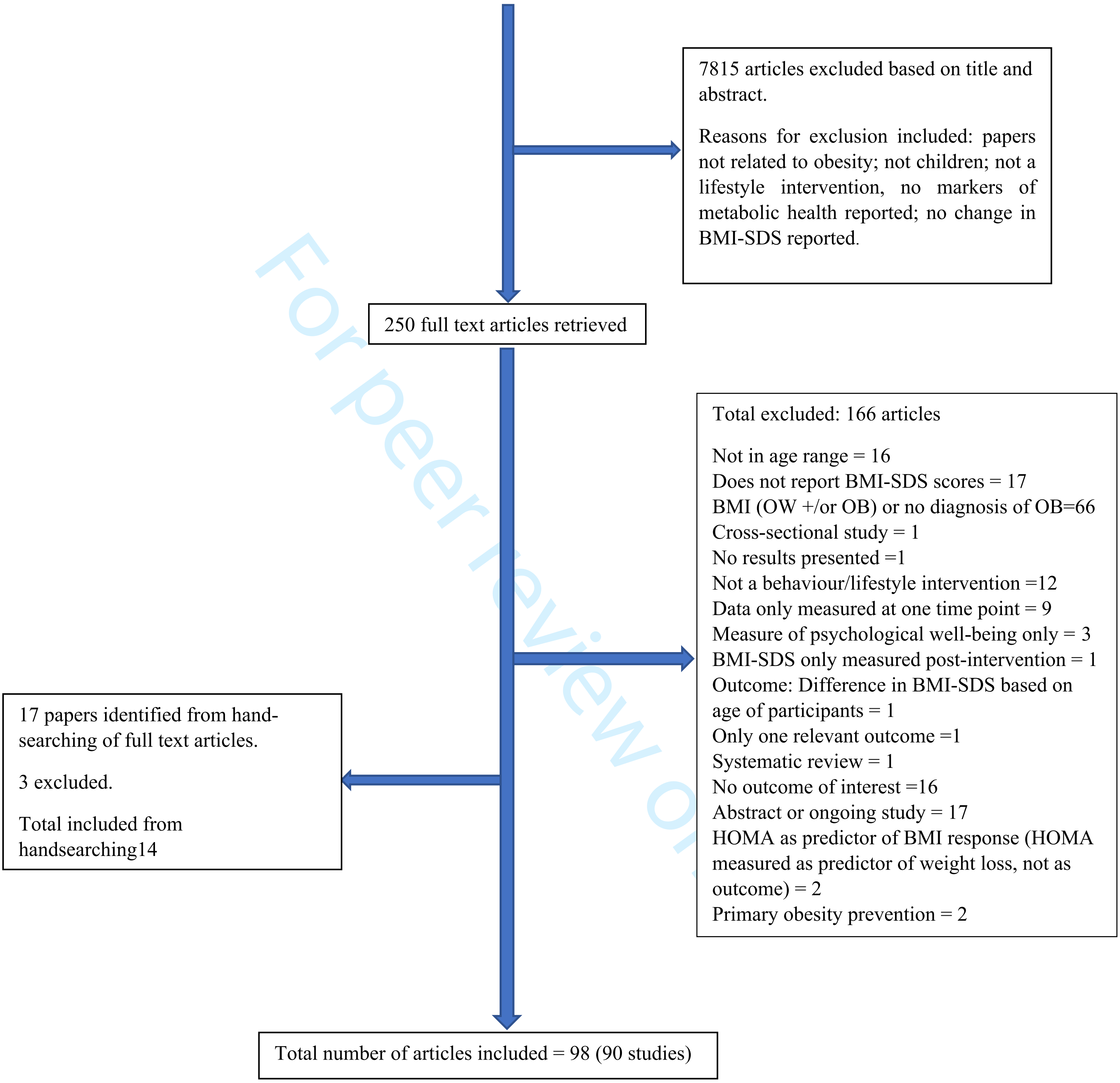

Figure 2: Venn diagram illustrating the markers of metabolic health measured

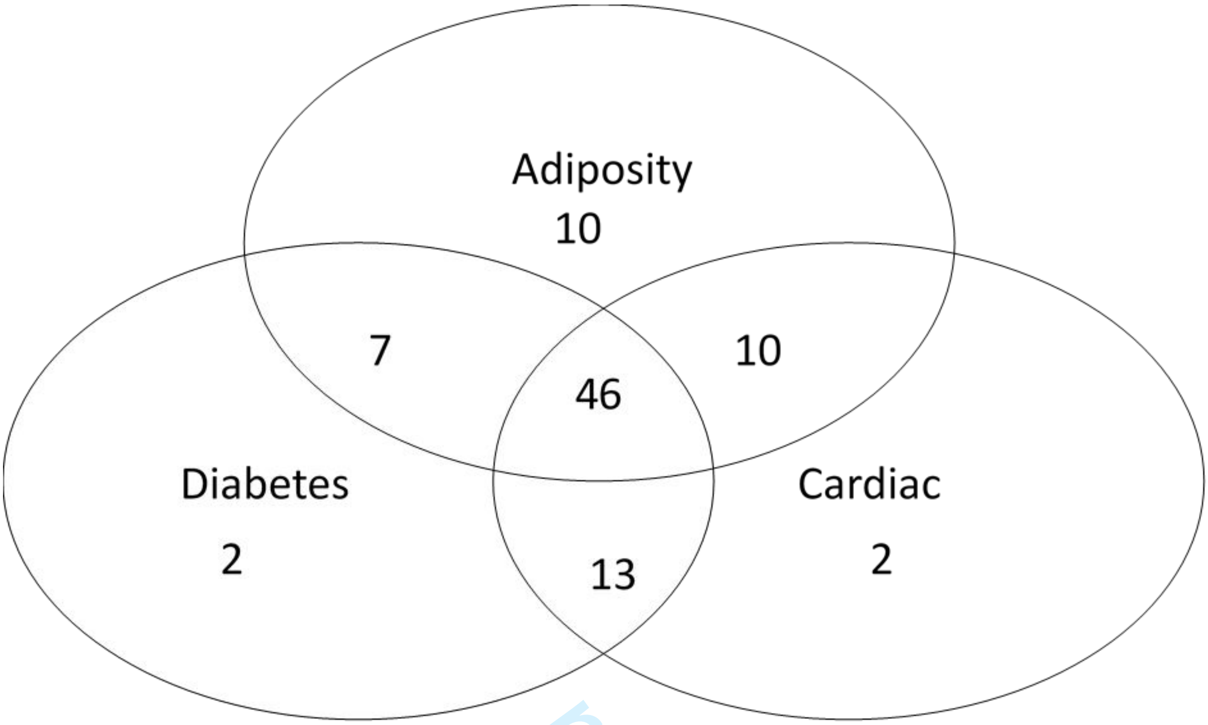

TOTAL NUMBER OF STUDIES = 90

review only

Figure. 3: Regression line showing the relationship between mean change in percentage body fat and BMI-SDS/z-score across the 39 studies (67 subsets) analysed

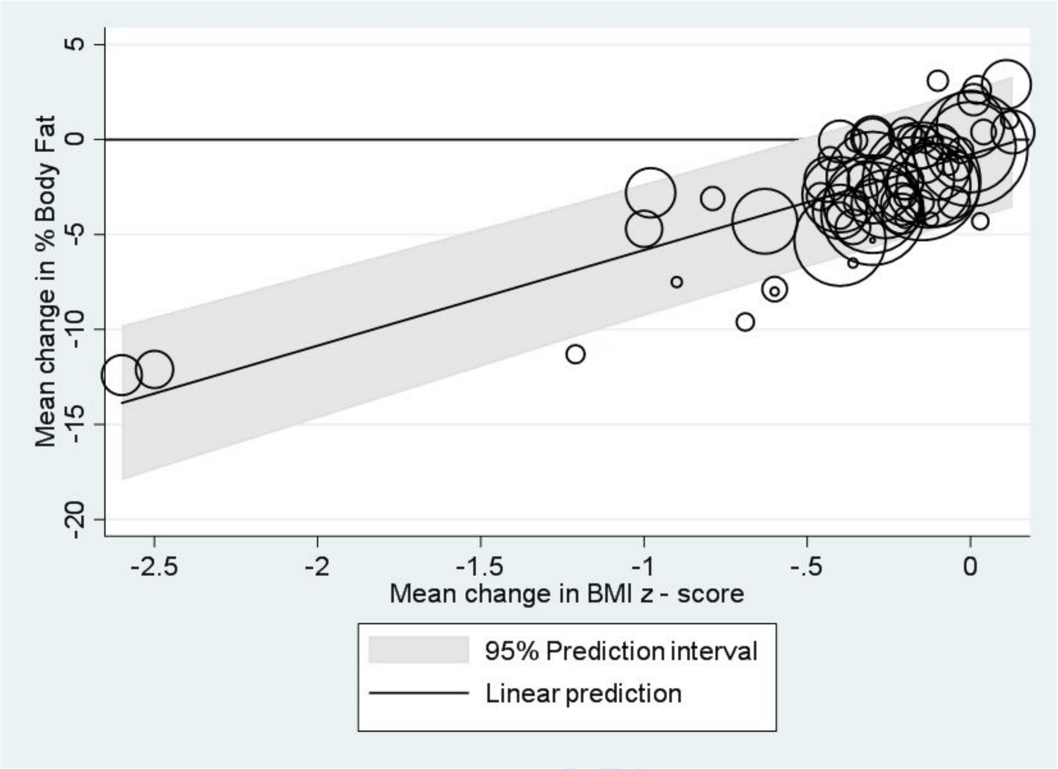

Figure 4: Normal plot to show the standardised predicted random effects from the meta-regression

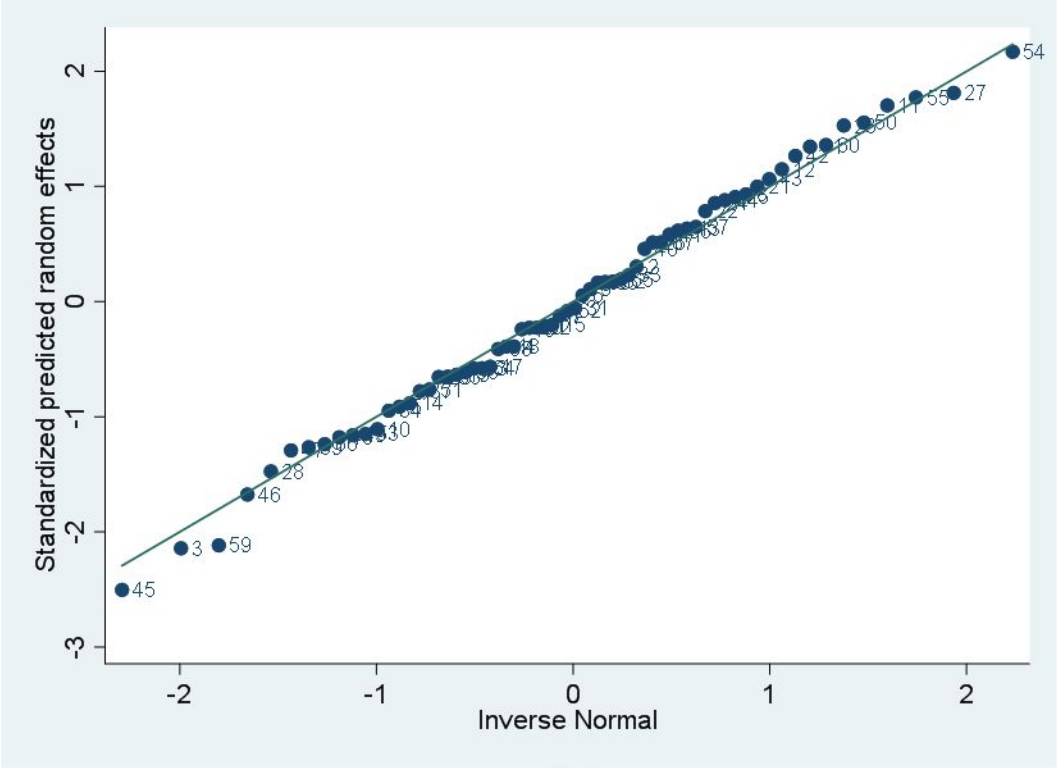

Figure 5: Sensitivity analyses

Figure 5(i): Analyses based on the 23 data sets where the SEs of the mean changes in %Body Fat were known (Fitted regression line: Mean change in %Body Fat = 4.482 x Mean change in BMI z-score - 0.856.)

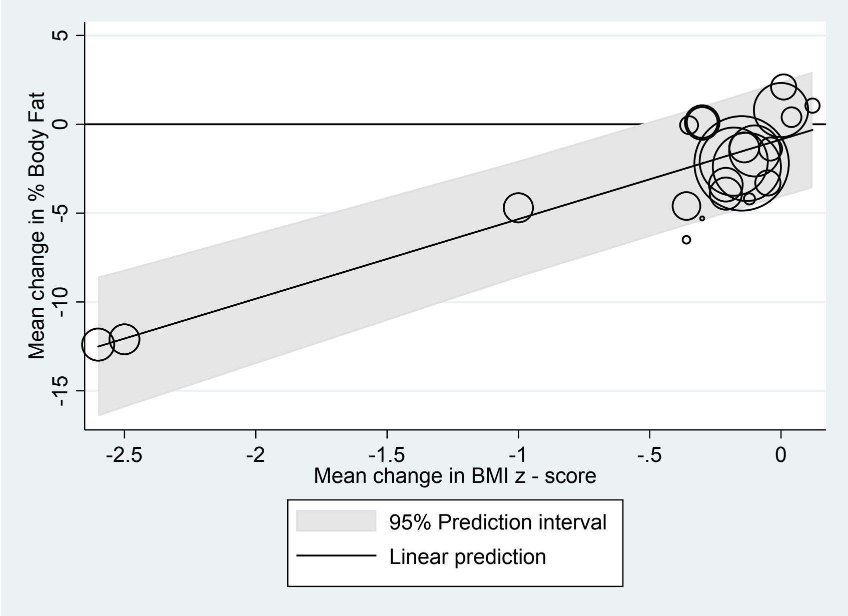

Figure 5(ii) Analysis using all the data subsets but excluding two extreme values (reduction of mean BMI z-score of more than 1.5) leaving 65 subsets. (Fitted regression line: Mean change in %Body Fat 7.082 x Mean change in BMI z-score - 0.334)

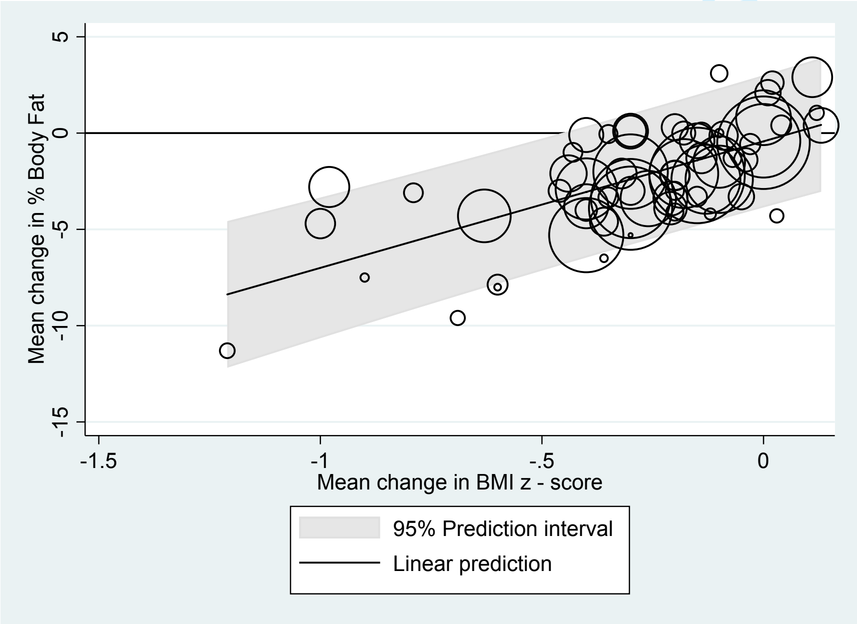

review only

Figure 5(iii) Analysis using all the data subsets but using a correlation coefficient of 0.50, rather than 0.81 to estimate the SE of the mean change in %Body Fat. (Fitted regression line was Mean change in %Body Fat = 5.033x Mean change in BMI z-score - 0.800)

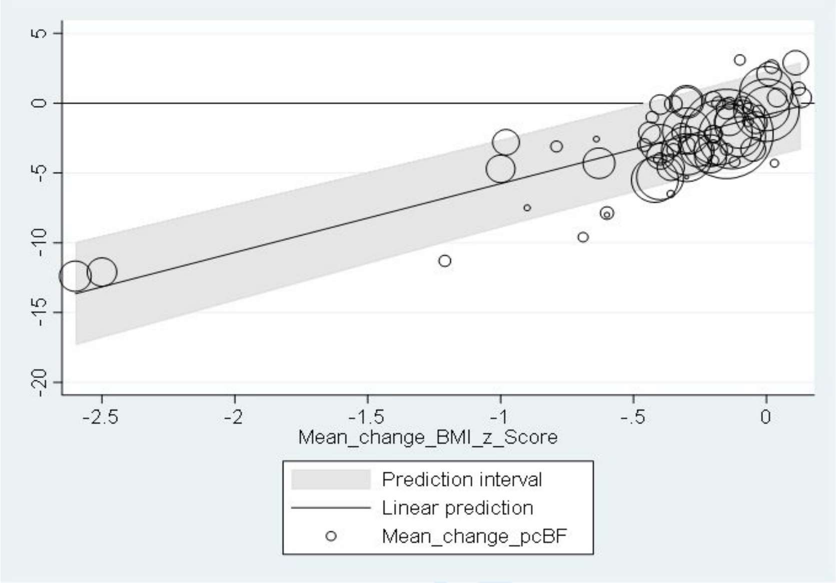

APPENDIX 1: Childhood obesity BMI systematic review_MEDLINE

1. exp Child/
2. exp Adolescent/
3. juvenile.tw.
4. exp Infant/
5. exp Pediatrics/
6. child$.tw.
7. infant$.tw.
8. teen$.tw.
9. p?ediatric$.tw.
10. young person.tw.
11. schoolchild$.tw.
12. youth.tw.
13. (boy$ or girl$).tw.
14. 1 or 2 or 3 or 4 or 5 or 6 or 7 or 8 or 9 or 10 or 11 or 12 or 13
15. exp body weight/
16. exp energy metabolism/
17. exp obesity/
18. exp childhood obesity/
19. exp metabolic syndrome X/
20. exp metabolic disorder/
21. (metabol$ adj1 disorder$).ti,ab.
22. (metabol$ adj1 syndrome$).ti,ab.
23. (cardiometabolic or cardio-metabolic or cardio metabolic).ti,ab.
24. (weight adj3 (cyc$ or reduc$ or los$ or maint$ or decreas$ or watch$ or control$ or gain$ or chang$)).tw.
25. (body fat or body fat percent$ or percent$ body fat or fat mass or adipos$).ti,ab.
26. waist-hip ratio$.tw.
27. waist circumferenc$.ti,ab.
28. (lean adj1 body adj1 mass).ti,ab.
29. (percentage adj1 body adj1 fat).ti,ab.
30. fat.ti,ab.

For peer review only

31. obes$.ti,ab.

32. (overweight or over weight or over-weight).ti,ab.

33. exp abdominal fat/

34. adipose tissue/

35. ((food or energy or calor$) adj1 intake).ti,ab.

36. (BMI or body mass ind$ or body-mass-ind$ or weight for height or weight-for-height).ti,ab.

37. (overfeed$ or over feed$).tw.

38. (overeat$ or over eat$).tw.

39. exp weight gain/

40. exp weight reduction/

41. (weight adj1 los$).ti,ab.

42. (fat adj1 los$).ti,ab.

43. 15 or 16 or 17 or 18 or 19 or 20 or 21 or 22 or 23 or 24 or 25 or 26 or 27 or 28 or 29 or 30 or 31 or 32 or 33 or 34 or 35 or 36 or 37 or 38 or 39 or 40 or 41 or 42

44. (BMI adj5 z score).af.

45. (BMI adj5 SDS).af.

46. (BMI adj5 standard adj1 deviation).af.

47. (Body adj1 mass adj1 index adj5 sd adj1 score).af.

48. (Body adj1 mass adj1 index adj5 SD).af.

49. 44 or 45 or 46 or 47 or 48

50. 14 and 43 and 49

For peer review only

Appendix 2: Characteristic of studies excluded from meta-regression

	Author, Year, Country (Intervention name)	Study design: Sample size (n) Analysed (An)	Obesity definition	Diet D)/ Exercise (E)/D+E: Setting	Format & content	Adiposity outcome measurement or reasons for exclusion for meta-regression
1.	Carraway 2014[84]<br>USA	Cohort<br>Total = 52 (subgroup n =33 & families offered FU for 10 months)	BMI > 95th %ile	Community	6 x 1 hr nutrition sessions + ad-lib access to a nutritious diet + 7hr/day PA + GT + CBT.	WC only
2.	Croker 2012[85]<br>UK	RCT:<br>Total = 72 (63)<br>FBBT= 37 (33)<br>OC =35 (30)	OW or Ob as per IOTF<br>Mean BMI SDS>3	D+E<br>Clinic (Hospital)	Reduce snacking ≤ 2 occasions/day + Balanced diet following 'Eatwell plate and 'Traffic Light system' + Reduce sedentary behaviour + 60 min/day exercise.	body mass, fat mass, WC &WC-SDS
3.	Doughty 2015[86]<br>USA	Cohort<br>Total = 12	BMI ≥ 89th %ile. Requested data from study authors regarding OB/OW– no response.	D+E<br>Academic Institution	Behavioural counselling + Daily caloric targets + 2 x 1hr/5 days and one 1hr/day physical training) + behavioural counselling.	WC. body mass
4.	Elloumi 2009[87]<br>Tunisia	RCT<br>Total =21<br>Energy restriction =7<br>Exercise training =7<br>Both =7	BMI > 97th %ile (French standards)	D+E<br>Academic Institution	2-month intervention. 3 groups, R = energy restricted group. E=individualised exercise group. RE= energy restriction + exercise. Individualized dietary advice by dietitian. 500kcal/day deficit (15% protein, 55% CHO, 30% fat). Exercise: 90 mins per day; 4 days per week. Intensity of exercise at heart rate corresponding to $lipox_{max}$	Body mass, fat mass
5.	Grulich-Henn, 2011[88]<br>Germany	Cohort Total = 58	BMI > 97th %ile (German paed. standards)	D + E<br>Academic Institution	6 x monthly nutritional consultation & CBT + 24 weekly PA programs.	Body mass only
6.	Gunnarsdottir, 2014[89]<br>Iceland	Cohort Total = 84	BMI z-score > 2.0 SDS (Swedish growth curve)	D+E<br>Clinic (Hospital)	Family-based Epstein behavioural intervention.	Body mass only
7.	Holm 2007[90]<br>Denmark	Cohort<br>Total enrolled =120; BL =110, post-intervention = 87	BMI-SDS LMS method (Danish ref pop.)	D+E<br>Academic Institution	Restricted low-fat diet (6500-7000 kJ/day) + Mandatory and optional PA.	number of pps not consistent for BMI-SDS and % body fat
8.	Kalavainen, 2012[91]<br>Finland	RCT<br>Total = 70<br>routine treatment = 35,<br>group treatment = 35	Wt-for-ht 115-182%	D+E<br>Community	2 interventions (Group and routine) - Routine (2 school heath care sessions) + Group (10 x 90 min/wk parents and children separate focusing on healthy lifestyle/physical activity session, then next 5 sessions/2 wks + 1 session together)	Fat mass only
9.	Kolsgaard, 2011[92]<br>Norway	Cohort<br>Total = 230 analysed (n= 307 started)	BMI > 97.5 %ile for ht according to Norwegian percentiles.	D+E<br>Clinic (Hospital)	~1hr biannual diet & PA (60 min/day exercise encouraged) counselling session with children & parent/s.	Body mass, WC
10.	Kolotourou 2013[93]<br>UK<br>**MEND**	Cohort: Recruited from MEND RCT.<br>Total = 230 analysed<br>Subsample 1 = 71 (6 mth RCT completers – both arms)	BMI ≥ 98th %ile	D+E:<br>Community	Family-based 9-week MEND program (2 x wk group sessions including nutrition education, behaviour modification + fun-based PA) + 12 wk free family swim pass.	WC only

For peer review only

		Subsample 2 = 42 (12 mth RCT completers – IG only)				
11.	Marcano, 2011[94]<br>Venezuela	Cohort<br>Total = 111	OW: BMI >90th %ile/BMI z-score > 1.5.<br>OB: BMI>97th %ile/BMI z-score >2	D+E:<br>Clinic (Hospital)	Nutrition+PA recommendations + A form to register wkly hours of PA, number of steps taken/day, and hrs/wk spent in sedentary activities + Restrict calorie intake and focus on a balanced diet encouraged.	Body fat only
12.	Mager 2015[95]	Cohort<br>Total=12 (completed =9)	CDC criteria	D Clinic (but unclear)	1 session of education for parents and children and then follow up at 3 months and 6 months afterwards.	WC only
13.	Makkes 2016[96]<br>Netherlands	RCT:<br>Total = 80<br>Short-stay (SS)=40<br>Long-stay (LS)=40	BMI-SDS ≥3.0 or BMI-SDS ≥2.3 + OB-related comorbidity	D+E:<br>Clinic (Hospital)	Intensive 12-month lifestyle treatment. In-patient period of either 2 months (short-stay group) or 6 months (long-stay group). Short-stay group: biweekly 2-day return visits for 4 months, then monthly 2-day return visits for 6 months following in-pt period. Long-stay group: monthly 2-day return visits for 6 months following in-pt period.<br>Treatment: Nutrition, physical activity and behaviour change. Required active participation of parents/caregivers.	WC,WC-SDS
14.	Martos, 2009[97]<br>Spain<br>(Same intervention as Valle Jimenez 2013[112] but different sample)	Cohort<br>Total = 47	BMI > 95th %ile on growth curves	D+E<br>Community	Moderately OB subjects (Low-calorie diet); Severe/refractory OB subjects (Restriction diet of 25-30%) + Moderate/intense exercise 60 min/day x 5 days/wk encouraged.	Body mass only
15.	Obert 2013[98]<br>France	Cohort<br>Total = 28 (plus 20 healthy lean controls)	BMI > 97th French %ile	D+E:<br>Clinic (Hospital)	Cycle ergometer (9 x 5 mins x 3 times/week: 4 min moderate + 1 min intense) + 2 times/wk moderate exercise for 1st 2 mths	Body mass, fat mass
16.	Panagiotopoulos2011[99]<br>Canada	Cohort<br>Total =119	OB: BMI ≥ 95th %ile; OW: BMI ≥ 85th %ile and <95th %ile with at least 1 comorbidity	D+E:<br>Clinic (Hospital)	10 x consecutive wkly group sessions (6-10 families): 30 min PA + nutrition session + behavioural session.	Body mass only
17.	Pedrosa, 2011[100]<br>Portugal	RCT<br>Total = 51 (OB grouped with OW in intervention individually conventional treatment and group-based treatment)	BMI z-score > 2	D:<br>Clinic (Hospital)	6 mths: Participants randomised to a hypocaloric LGI or HGI diet (matched for macronutrient composition).	Body mass only
18.	Pozzato[101]<br>Verduci 2011[102]<br>Italy	Cohort:<br>Total = 26	>30kg/m2 age and sex adjusted Cole *et al* curve	D+E.<br>Community and Clinic	Normocaloric balanced diet and active lifestyle based on italian guidelines for treatment of childhood obesity	WC only
19.	Reinehr, 2004b[103]<br>Germany<br>OBELDICKS	Cohort:<br>Total = 57	BMI ≥ 97th %ile	D+E:<br>Clinic (Hospital)	Obeldicks - Intensive phase 3 mnths (Parents' course 2x/mnth + Behaviour therapy 2x/mnth + Nutritional course 2x/mnth + Exercise therapy 1x/wk) + Establishing phase 3 mnths (Talk rounds for parents 1x/mnth + Psychological therapy + Exercise therapy 1x/wk) + Establishing phase 2 for 3 mnths (Psychological therapy + Exercise therapy 1x/wk) + Establishing phase 3 for 3 mnths (Exercise therapy 1x/wk).	Body fat only
20.	Reinehr, 2009[104]<br>Germany<br>OBELDICKS	Cohort<br>Total = 109 (plus 43 obese controls)	IOTF criteria: OB	D+E:<br>Clinic (Hospital)	Obeldicks (as above)	WC only

For peer review only

21.	Rijks 2015[105] Netherlands	Non-randomised prospective study Total = 172	IOTF criteria: OW, OB, MO	D+E: Clinic (Hospital)	Guidance with focus on nutrition, food habits, PA, sleep, psychological and social aspects.	WC-SDS only
22.	Rovira 2013[106] Spain	Cohort Total = 110	BMI ≥ 97th %ile	D+E Clinic (Hospital)	12 x monthly visits in 2 phases: motivational and intervention. Focus on promoting healthy eating, encouraging PA & decreasing sedentary behaviour.	Only reported as 'good responders' and 'poor responders' to intervention so removed from analysis
23.	Santomauro 2011[107] Venezuela	Cohort Total = 36	BMI > 97th %ile	D+E: Clinic (Hospital)	Dietary recommendations + 30 mins daily moderate exercise or 3 x wk moderate exercise + decrease time watching TV/video games.	Body mass, fat mass
24.	Schum 2012[108] Germany	Cohort: Total = 75 HZ=52 HO= 21	BMI-SDS > 2	E: Community	Increase to 2 hrs/day PA + nutritional recommendations based on 'Optimised Mixed Diet for German Children and Adolescents' + close surveillance by physician.	Fat mass, WC
25.	Shalitin, 2009[109]	Cohort: Total = 174 randomised E =58 (52) D =58 (55) D+E = 58 (55)	BMI > 95th %ile for age & gender	D+E: Clinic (Hospital)	3-month interventions: Exercise intervention (90 min moderate exercise 3 days/wk); Diet intervention 3 mths (12 x/wk nutritional group meetings with parents + Hypocaloric diet 1200 kcal/day); Diet and exercise intervention 3 mths (90 min training session days/wk + 12 x/wk nutritional group meetings with parents + Hypocaloric diet 1200 kcal/day).	SEs were calculated from a mixed model and not directly comparable
26.	Springer 2015[110] Germany	Cohort: Total=39	BMI > 90th %ile	D+E: Clinic (Hospital)	Encouraged to increase exercise by 1-2 hrs/day + Decrease sedentary behaviour to a total of 2 hrs/day or less + Nutrition recommendations + 6 telephone calls from/visits to the physician.	fat mass, WC & WC-SDS
27.	Tan-Ting 2011[111] Philippines	Cohort: Total = 44	BMI ≥ 95th %ile (CDC)	D+E: Clinic (Hospital)	Multidisciplinary, individualised, behavioural modification and exercise programme (St Luke's Medical Center Obesity & Weight Management Program) Dietary session (6 sessions over 3 mths) + Restricted diet (1200-1500 Kcal/day) + Physical activity (24 x 1hr sessions over 3 mths + encouraged to do ≥ 30 min of individual exercise) + Behavioural management (4 x sessions over 3 mths).	body mass, fat mass, WC &WC-SDS
28.	Valle Jiminez 2013[112] Spain	Cohort Total = 50 (plus n=50 non-obese control)	BMI >95th percentile growth curves for Spanish pop.	D+E: Academic Institution/Clinic (Hospital)	Behavioural components, physical exercise and nutritional education. Energy distribution of diet: 25% between breakfast & lunch; 30-35% at lunch; 15% afternoon snack; remainder dinner. Moderate-to-intense PA for 30 mins at least 3 days per wk. Aim that 1 month after the start of tx subjects should be engaging in 60 mins/day moderate-to-intense physical exercise.	Body mass only
29.	Vanhelst 2013[113] France	Cohort Total=37	ND	D+E: Community	2hr/wk exercise sessions + 2hr/3 months heath education session.	Fat mass, fat free mass
30.	Vasquez 2013[114] Chile	Cohort X-over trial (Group 1 only) Total = 60	BMI ≥ 95th %ile CDC	D+E: Academic Institution	Group nutrition education sessions x 6 (5 for children; 1 for parents) Psychologist support sessions x 6 (5 for children; 1 for parents) PE 45 mins x3/wk (30 sessions in total)	Body mass, WC
31.	Verduci 2015[115] Italy	Cohort: Total = 85	BMI Cole's curve cut-off 30 kg/m^2 at 18 yrs	D+E: Clinic (Hospital)	Normocaloric balanced diet + 60 min/day moderate/vigorous exercise + 1 hr educational session with dietician at recruitment.	WC only
32.	Vos, 2011[116] Netherlands	RCT Total = 81 (BL: 79 An. 69) I = 41 (BL 40: An. 36) OC=40 (BL 39: An. 33)	Cole *et al* criteria	D+E: Clinic (Hospital)	12 mths: During first 3 mths (7 x 2.5 hr/2 wks children group meetings + 5 x 2.5 hr/2 wks parent meetings + 1 x 2.5 hr/2 wks child/parent meeting + 2-3 refresher follow-up sessions for total of 2 yrs). Also included exercise however not described except in flow diagram	WC-SDS only

For peer review only

33.	Weiss 2009[117]<br>USA<br>Yale **TEAMS**	Cohort:<br>Total = 186	BMI > 95th %ile (CDC)	D+E:<br>Clinic (Hospital)	Subjects followed biannually as outpatients + Received nutritional/PA guidance. Levels of adherence to these recommendations was not evaluated or documented	Body mass only
34.	Weigel 2008[118]<br>Germany	RCT:<br>Total = 73<br>IG = 37<br>OC = 36	OW BMI > 90th %ile<br>OB BMI > 97th %ile<br>Extremely OB BMI > 99.5th %ile	D+E:<br>Community	Twice wkly 45-60 min sessions on exercise/dietary education/coping strategies.	Fat mass only
35.	Wong 2009 [119]<br>USA	Cohort:<br>Total = 21	BMI ≥ 95th %ile	D+E:<br>Community	6 x 1hr behavioural lessons + 4 x 1hr PA/ nutrition lessons + 1800 kcal/day diet.	body mass only

KEY: %ile = percentile; AmO = Outpatient Ambulatory; An. = analysed; apt. = appointment; BB =Bright Bodies; BFC = Better food choices; BL = baseline; BM = behaviour modification; BMI= body mass index; C = control; CG: control group; CBT = cognitive behavioural therapy; CDC = Centre for Disease Control; CG = control group; CHO = carbohydrate; D = diet; E = exercise; FBBT = family-based behavioural treatment; F = female; FU = follow up; GI = glycaemic index; GT = group therapy; HGI = high glycaemic index; hr = hour; HZ = heterozygous; HO = homozygous; ht = height; I = intervention; IG= intervention group; IOTF = International Obesity Task Force; Inpt. = inpatient; LGI = low glycaemic index; LMS= least-mean-squares; LS = long stay; min= minute; mth = month; MO = morbidly obese; norm. normal; n = number; NAFLD = Non-alcoholic fatty liver disease; ND = not described; NR = not reported; OB = obese; OC = obese control; OW = overweight; paed. = paediatric; PA = physical activity; PE = physical activity; PROT= protein; ppts= participants; RCT = randomised controlled trial; SD = standard deviation; SDS = standard deviation score; SE: standard error; SMP= Structured meal plan; SS= short stay; SMC= structured modified carbohydrate diet; trad. = traditional; Trad. act = traditional activity; tx = treatment; TEAMS = Tracking Endpoints in Adolescent MS; wk = week; WList OC– wait list obese control; WL = weight loss; wt = weight; X-over = crossover; yr = year

For peer review only

Appendix 3: Regression line showing the relationship between mean change in percentage body fat and BMI-SDS/z-score across the 39 studies (67 subsets) analysed, with two studies of younger children highlighted

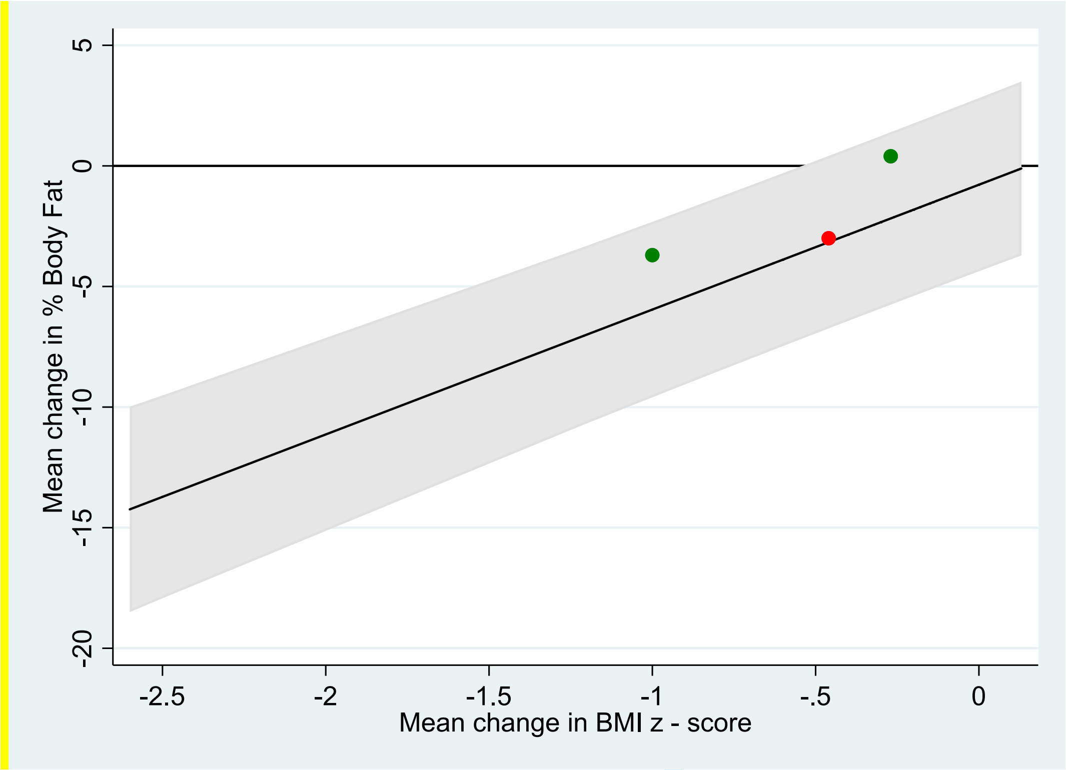

- participants 4-8 years. Dobe et al., 2011 [37]
- participants 5-10 years. Gajewska et al., 2016 [41]

For peer review only - http://bmjopen.bmj.com/site/about/guidelines.xhtml

BMJ Open

What change in body mass index is associated with improvement in percentage body fat in childhood obesity? A meta-regression

Journal:	*BMJ Open*
Manuscript ID	bmjopen-2018-028231.R1
Article Type:	Research
Date Submitted by the Author:	12-Apr-2019
Complete List of Authors:	Birch, Laura; NIHR Bristol Biomedical Research Centre - Nutrition<br>Perry, Rachel; NIHR Bristol Biomedical Research Centre - Nutrition<br>Hunt, Linda<br>Matson, Rhys; NIHR Bristol Biomedical Research Centre - Nutrition<br>Chong, Amanda; NIHR Bristol Biomedical Research Centre - Nutrition<br>Beynon, Rhona; NIHR Bristol Biomedical Research Centre - Nutrition<br>Shield, Julian; NIHR Bristol Biomedical Research Centre - Nutrition
**Primary Subject Heading**:	Paediatrics
Secondary Subject Heading:	Nutrition and metabolism, Public health
Keywords:	obesity, childhood, adolescence, body mass index, body fat

SCHOLARONE™ Manuscripts

What change in body mass index is associated with improvement in percentage body fat in childhood obesity? A meta-regression.

Laura Birch[a], RD MSc, Rachel Perry[a], PhD, Linda P Hunt[a], PhD, Rhys IB Matson[a], MSc, Amanda Chong[a], BSc, Rhona Beynon[a], MSc, Julian PH Shield[a,b] MD

Affiliations: [a] NIHR Bristol Biomedical Research Centre – Nutrition Theme, Level 3 University Hospitals Bristol Education Centre, Upper Maudlin Street, Bristol.

[b] University Hospitals Bristol NHS Foundation Trust, Level 6 University Hospitals Bristol Education Centre, Upper Maudlin Street, Bristol.

Address correspondence to: Laura Birch, NIHR Bristol Biomedical Research Centre – Nutrition Theme, Level 3 University Hospitals Bristol Education Centre, Upper Maudlin Street, Bristol, UK, BS2 8AE, [laura.birch@bristol.ac.uk], +44 (0)117 3421763

Rachel.Perry@bristol.ac.uk
L.P.Hunt@bristol.ac.uk
Rhys20@me.com
ac14629@bristol.ac.uk
rhona.beynon@bristol.ac.uk
J.P.H.Shield@bristol.ac.uk

Short title: BMI change to reduce body fat in childhood obesity

Financial Disclosure: The authors have no financial relationships relevant to this article to disclose.

Funding Source: This study was supported by the NIHR Biomedical Research Centre at University Hospitals Bristol NHS Foundation Trust and the University of Bristol. The views expressed in this publication are those of the authors and not necessarily those of the NHS, the National Institute for Health Research or the Department of Health.

Conflict of Interest: Professor Hamilton-Shield is a lead author on two studies included in the systematic review that this paper reports on. The other authors have no conflicts of interest to disclose.

Key words: obesity, childhood, adolescence, body mass index, body fat

Abbreviations:
BMI-SDS: body mass index- standard deviation score
RCT: randomised controlled trial
IOTF: International Obesity Task Force
HOMA: Homeostatic model assessment
LDL: Low-density lipoprotein
HDL: High-density lipoprotein
IL6: Interleukin 6
ALT: Alanine transaminase
HTA: Health Technology Assessment
SD: standard deviation
SE: standard error

For peer review only

For peer review only - http://bmjopen.bmj.com/site/about/guidelines.xhtml

CI: Confidence interval
PI: Prediction interval
IQR: Interquartile range

Word count: 4050

For peer review only

For peer review only - http://bmjopen.bmj.com/site/about/guidelines.xhtml

Abstract

Objective: Using meta-regression this paper sets out the minimum change in BMI-SDS required to improve adiposity as percentage body fat for obese children and adolescents.

Design: Meta-regression.

Setting: Studies were identified as part of a large-scale systematic review of the following electronic databases: AMED, Embase, MEDLINE via OVID, Web of Science and CENTRAL via Cochrane library.

Participants: Individuals aged 4–19 years with a diagnosis of obesity according to defined BMI thresholds.

Interventions: Studies of lifestyle treatment interventions that included dietary, physical activity and/or behavioural components with the objective of reducing obesity were included. Interventions of less than 2 weeks duration and those that involved surgical and/or pharmacological components (e.g. bariatric surgery, drug therapy) were excluded.

Primary and secondary outcome measures: To be included in this review, studies had to report baseline and post-intervention BMI-SDS or change measurements (primary outcome measures) plus one or more of the following markers of metabolic health (secondary outcome measures): adiposity measures other than BMI; blood pressure; glucose; inflammation; insulin sensitivity/resistance; lipid profile; liver function. This paper focuses on adiposity measures only. Further papers in this series will report on other outcome measures.

Results: This paper explores the potential impact of BMI-SDS reduction in terms of change in percentage body fat. Thirty-nine studies reporting change in mean percentage body fat were analysed. Meta-regression demonstrated that reduction of at least 0.6 in mean BMI-SDS ensured a mean reduction of percentage body fat mass, in the sense that the associated 95% prediction interval for change in mean percentage body fat was wholly negative.

Conclusions: Interventions demonstrating reductions of 0.6 BMI-SDS might be termed successful in reducing adiposity; a key purpose of weight management interventions.

Trial registration: The main review is registered on PROSPERO international prospective register of systematic reviews (PROSPERO 2016 CRD42016025317).

For peer review only

Article Summary

Strengths and limitations of this study

- We believe that this is the first paper to attempt to bring together all studies that have reported both a change in BMI-SDS and changes in a marker of adiposity in the obese paediatric population.
- The systematic methods employed to identify the included studies were stringent, but it is possible that some relevant studies might have been missed.
- There was some variation in the reporting of results where there were multiple publications of the same study; in these cases, the results from the most comprehensive paper have been used.
- Studies that did not report change in mean percentage body fat could not be included in this meta-regression.

For peer review only

Introduction

Childhood obesity is one of the most serious global public health challenges of the twenty-first century[1]. In England, the latest figures from the National Child Measurement Programme, which measures the height and weight of around one million school children every year, showed that 9.5 percent of children aged 4–5 years and 20.1 percent of those aged 10–11 years were obese[2,3]. Childhood obesity has adverse health consequences in both the short-and long-term; including an increased risk of developing metabolic disturbances, including hypertension, dyslipidaemia and insulin resistance, and becoming obese adults[4]. The presence of adverse changes in cardiac and vascular function and type 2 diabetes, which were previously considered adult morbidities, now being identified in obese children and adolescents[5-11] illustrates the urgent need for effective weight management treatment interventions to reduce adiposity and improve the metabolic health status of the paediatric population.

Moderate weight loss has been shown to have a positive impact on many metabolic and cardiovascular risk factors[12,13]. Weight management interventions for obese adults that result in a 5-10 percent decrease in body weight are associated with significant improvements in blood pressure, serum lipid levels and glucose tolerance[14] and reduction in the prevalence of

For peer review only - http://bmjopen.bmj.com/site/about/guidelines.xhtml

hypertension and diabetes[15]. Minimum weight management targets can therefore be set to improve metabolic health in this population[16].

During childhood, all measurements over time are complicated by the influence of growth, meaning that cut-offs routinely used in the adult population cannot be used in children and adolescents. However, measured values of body mass index (BMI) can be standardized into standard deviation scores (SDS) in respect to reference populations[17]. These standardised scores, referred to as BMI-SDS throughout this paper, provide a normalised measurement for the degree of obesity in children and young people, indicating to what degree an individual BMI lies above or below the median BMI value.

A meta-analysis by Ho *et al* [18] concluded that lifestyle interventions can lead to improvements in weight and cardio-metabolic outcomes in child obesity. However, whilst numerous lifestyle intervention programmes to tackle childhood obesity are conducted across the UK, and many describe statistically significant reductions in BMI-SDS[19], these results do not necessarily translate into clinical benefit for the individual. How reducing BMI-SDS in a trial translates to a reduction in adiposity is uncertain.

Paediatric weight management guidelines exist in many countries to promote best practice, but at present many of these recommendations are based on low-grade scientific evidence[20]. Understanding how much BMI must be reduced to positively affect body composition and metabolic health is important to ensure that treatment interventions are appropriately designed and evaluated[21].

For peer review only - http://bmjopen.bmj.com/site/about/guidelines.xhtml

Given the scale of the obesity problem and the significant and sustained adverse effects on health, clinically effective paediatric weight management treatment options are vital. A meta-analysis of cardiovascular disease risk in healthy children and its association with BMI has been conducted[22] but there is yet to be a systematic quantification of the reduction in BMI required to improve adiposity in the obese paediatric population.

It is important to highlight that when assessing interventions designed to manage overweight and obesity in children and adolescents, it is essential to recognise that measures such as BMI and derived SDS scores are surrogates of the real purpose: reduction of adiposity, fat being the key organ involved in metabolic complications[23]. To rigorously assess the clinical and cost effectiveness of weight management interventions in young people, it is first necessary to understand what BMI-SDS change means in terms of key outcomes such as effects on adiposity. This paper is designed to put BMI-SDS changes in context when considering improvement in adiposity (fatness). Through meta-regression analysis we explore the potential impact of BMI-SDS reduction in terms of change in percentage body fat. The outcome of which will both inform clinical guidelines for paediatric weight management interventions and guide outcome measures in future clinical trials.

Objective

This paper aims to establish the minimum change in BMI-SDS needed to effect improvements in adiposity markers of obese children and adolescents. This is the first of a series of three papers reporting on the findings from studies identified in a large systematic review (N=90 studies; searched up to May 2017) and focuses on the evidence in relation to adiposity (percentage body fat); the others relating to metabolic and cardiovascular health.

For peer review only - http://bmjopen.bmj.com/site/about/guidelines.xhtml

Methods

The studies included in this paper were identified as part of large-scale systematic review (PROSPERO CRD42016025317). The protocol for this systematic review is available: https://doi.org/10.1186/s13643-016-0299-0. The final search was conducted in May 2017, the review was completed in January 2018 and the results are still being evaluated.

Participants

Studies with participants aged 4–19 years with a diagnosis of obesity using defined BMI thresholds were considered for inclusion. BMI-SDS was calculated as a function of the degree of obesity of the subjects when compared with BMI references. BMI standards included, but were not limited to, the 98th centile on the UK 1990 growth reference chart[24], 95th percentile on the US Centre for Disease Control and Prevention growth chart[25], the International Obesity Taskforce (IOTF) BMI for age cut-points[26] and the World Health Organisation growth references[27,28], in addition to country-specific obesity thresholds using BMI reference data from their paediatric populations. Studies that included overweight, as opposed to obese, individuals, pregnant females, or those with a critical illness, endocrine disorders or syndromic obesity were excluded from this review.

Interventions

Studies of lifestyle treatment interventions that included dietary, physical activity and/or behavioural components with the objective of reducing obesity were included. Interventions of less than 2 weeks duration and those that involved surgical and/or pharmacological components (e.g. bariatric surgery, drug therapy) were excluded. Studies focused on obesity prevention were also excluded. No restrictions were imposed regarding the setting or delivery of the interventions.

For peer review only - http://bmjopen.bmj.com/site/about/guidelines.xhtml

For peer review only

Outcome measures

To meet the inclusion criteria of the full systematic review, interventions had to report baseline (pre-) and post-intervention BMI-SDS or change measurements of BMI-SDS plus one or more markers of metabolic health (please refer to the published protocol paper for a complete list of the metabolic health markers of interest; https://doi.org/10.1186/s13643-016-0299-0).

This paper focuses on change in BMI-SDS and adiposity measures other than BMI, including waist circumference and percentage body fat.

Study design

Completed, published, randomised controlled trials (RCTs) and non-randomised studies (cohort studies) of lifestyle treatment interventions for obese children and adolescents, with or without follow-up.

Ethics

Ethical approval was not required as this paper reviewed published studies only.

Patient and Public involvement

There was no patient or public involvement in this review of published studies.

Information sources and search methods

Studies were identified by searching five electronic databases from inception to May 2017 (AMED, Embase, MEDLINE via OVID, Web of Science and CENTRAL via Cochrane library), alongside scanning reference lists of included articles and through consultation with experts in the field. The search strategy for MEDLINE database is presented in Appendix 1.

For peer review only - http://bmjopen.bmj.com/site/about/guidelines.xhtml

Study Selection and data extraction

Titles and abstracts were assessed for eligibility and the data outcome measures described previously were extracted by two independent reviewers from the review team (LB, AC, RP, RB) using a standardised data extraction template, which was piloted by both reviewers before starting the review to ensure consistency.

Data availability

No additional data available.

Quality assessment

The focus of this study is the relationship between change in BMI-SDS and change in metabolic health parameters, rather than the specific treatment interventions that effect those changes. Therefore, risk of bias tools, such as the Cochrane Risk of Bias tool[29], were not considered appropriate. The included studies were assessed for methodological quality by two members of the review team during the data extraction process using the Quality Assessment tool utilised in the 2004 Health Technology Assessment (HTA) systematic review of the long-term effects and economic consequences of treatments for obesity and implications for health improvement[30]. This Quality Assessment tool comprises 20 questions which are added together to give a final score and a percentage rating, from which a level of quality is assigned. Any discrepancies in Quality Assessment scoring were resolved through discussion.

Analysis

We carried out random-effects meta-regression as implemented in Stata[31] to try to quantify the relationship between mean change in BMI-SDS (independent, predictor variable) and mean change in percentage body fat (target variable), where these were either reported, or were able

For peer review only - http://bmjopen.bmj.com/site/about/guidelines.xhtml

to be calculated from reported data. Further details are given below. We were not trying to assess the relative effects of the various interventions, but rather to examine the relationship between these two outcomes. Meta-regression allows for residual heterogeneity in the target variable not explained by the predictor. Subsets from the same study (e.g. intervention vs control, boys vs girls, see below) were regarded as independent observations provided there was no data duplication.

Results

Search Results

In total, 98 published articles relating to 90 different studies met the inclusion criteria for the entire systematic review. See Figure 1 for a flow diagram illustrating the number of papers excluded at each stage of the review. For studies reported in multiple publications, the reference that provided the most comprehensive information has been used (see footnote of Table 1 for details).

Figure 1: Flow diagram from the systematic review that identified the included studies

The Venn diagram (Figure 2) illustrates how many studies were identified for the various markers of metabolic health. Seventy-three studies assessed and reported adiposity measures. The adiposity measures reported included percentage body fat, body fat-SDS, body mass, fat mass, fat-free-mass, waist circumference and waist circumference-SDS. The 68 studies that examined diabetes/inflammation measures (HOMA-IR, insulin, glucose, C-reactive protein, Interleukin-6, Alanine transaminase and the 71 studies examining cardiac measures (e.g. lipids, cholesterol, blood pressure) will be reported separately.

For peer review only - http://bmjopen.bmj.com/site/about/guidelines.xhtml

Figure 2: Venn diagram illustrating the markers of metabolic health measured

Studies for inclusion in meta-regression analysis

Seventy-three studies assessed and reported adiposity measures. Of the different adiposity measures that were reported in these studies (percentage body fat, body fat-SDS, body mass, fat mass, fat-free-mass, waist circumference and waist circumference-SDS), we elected to examine percentage body fat as it was far more frequently reported across studies. Therefore, of the 73 adiposity studies, we conducted our meta-regression on 39 studies which reported percentage body fat values. These studies are presented in Table 1 with the corresponding changes in BMI-SDS.

The results of five studies were duplicated in multiple papers, thus the reference that reported the most comprehensive information was used in the analysis; see Table 1 footnote for details. Thirty-four studies were excluded from the meta-analysis; the characteristics of the excluded studies, along with the reason for exclusion, are summarised in Appendix 2.

For peer review only - http://bmjopen.bmj.com/site/about/guidelines.xhtml

Table 1: Characteristics of studies reporting adiposity outcomes with results of mean change in BMI-SDS and percentage body fat

	Author, Year, Country (Intervention name)	Study design: Sample size (n) Analysed (An)	Obesity definition	Age range (inclusion): Mean ± (SD) Sex (% F)	Pubertal status measured	Diet D)/ Exercise (E)/D+E: Setting	Format & content	Duration (mths) Follow up (mths)	Method of % body fat measurement	Δ BMI SDS/z-score by subgroup when reported	Δ % body fat score by subgroup when reported
1	Bell 2007[32] Australia	Cohort Total = 14 (14)	BMI ≥ 95th %ile	Age range:9-16 12.70(2.32); F=43%	Yes- Tanner	E: community	8 weeks structured circuits exercise training: 3 x 1hr sessions/week. No standard dietary modifications.	2: 0	DXA	All:-0.03	All:-0.57
2	Bock 2014[33] Canada **HIP KIDS**	Cohort: Total = 42 (41)	BMI ≥ 95th %ile (CDC)	Age range: 8-17 12.8 ± 3.14; F=50%	Yes - Tanner	D+E: Clinic (Hospital)	Intensive phase (3 mths): bi-wkly 90 min counselling. Maintenance phase (9 months): alternating mthly GP or individual sessions (90 mins). Sessions focus on exercise/psychosocial/behavioural aspects.	12: 0	BIA	All: -0.04	All: -1.39
3	Bruyndonckz, 2015[34] Belgium	Quasi-RCT: Total = 61 IG = 33 (27) CG = 28 (21)	BMI ≥ 97th %ile adolescents <16 yrs; BMI ≥ 35 adolescents ≥ 16 yrs	Age range:12-18 IG: 15.4 ± 1.5; F = 79% CG: 15.1 ± 1.2; F=73%	NR	D+E: Clinic (Hospital)	Intervention: Dietary restriction 1500-1800 kcal/day + 2 hrs/day supervised play/lifestyle activities + 2hrs/wk PE + 3 x 40min/wk supervised training session. Control: Usual care.	10: 0	Subsample also measured using DXA	IG: -1.21 CG: 0.13	IG: -11.30 CG: 0.4
4	Bustos 2015[35] Chile	Cohort: Total = 50 (28 completed)	CDC	Age range: NR 9.5 ± 1.9; F=48%	NR	D+E: Academic Institution	Nutrition/behavioural modification session 40 min/wk + PA 50 min x2/wk+ Family support every 15 days for first 2 mths, then mthly.	8: 0	DXA	All: -0.3	All: -3.00
5	Calcaterra 2013[36] Italy	Cohort: Total = 22 (22)	BMI > 95th %ile	Age range: 9-16 13.23 ± 1.76; F=41%	Yes - Tanner	E: Academic Institution	2 x 90 mins exercise training sessions/wk	3: 0	BIA	All: -0.15	All: -3.30
6	Dobe 2011[37] Germany OBELDICKS – mini	Cohort: Total = 103 (103)	>97th to 99.5 percentile	Age range: 4-8 6.1 ± 1 F=56%	NR	D+E: Academic Institution	Obeldicks mini: focus on training parents (22.5 hrs for parents, 4.5 hrs for children). Group sessions. Parents+children classes every 4th session,<br>Children's classes: 9 x monthly sessions (30 mins): 1 x introduction; 3 x diet; 5 x eating habits<br>Parenting classes: 13 x monthly sessions (1.5 hrs): 1x introduction 1x medicine 3x nutrition 5x eating habits + education tips 3x discussion circle<br>Individual consultation: every 2 months (30 mins)<br>Exercise: 50 x weekly sessions (1.5 hrs)	12: 0	BIA	Obeldicks-mini: -0.46	Obeldicks mini: -3.00

For peer review only - http://bmjopen.bmj.com/site/about/guidelines.xhtml

7	Farpour-Lambert, 2009[38] Switzerland	RCT:<br>Total = 44<br>IG= 22 (22)<br>OC =22 (22)	BMI > 97th %ile	Age range: 6-11<br>8.9 ± 1.5<br>IG: F=59%<br>OC: F= 68%	Yes	E<br>Clinic (Hospital)	180 min/wk PA + 135 min/wk PE	3:<br>0	Skinfold measurements	IG: -0.1<br>CG: 0	IG: -1.50<br>CG: 0.80
8	Ford, 2010a,b UK [39,40]	RCT:<br>Total = 106 (91)<br>Gp1 SC = 52 (46)<br>Gp 2 Mandometer = 54 (45)	BMI ≥ 95th %ile (CDC)	Mandometer: 9.0 - 16.9<br>SC: 9.1 - 17.5<br>Mandometer: 12.7 ± 2.2<br>SC: 12.5 ± 2.3<br>overall F=56%	Yes	D<br>Clinic (Hospital)	Mandometer device to regulate rate of eating and total intake vs SC	12:<br>0	DXA	IG:-0.36<br>CG:-0.14	IG:-4.60<br>CG:-1.30
9	Gajewska, 2016[41] Poland	Cohort:<br>Total = 100 (76)<br>With WL =71 (56)<br>Without WL = 29 (20)	BMI SDS > 2	Age range: 5-10<br>with WL: 8.1(6.8-9.2); F= 51%<br>without WL: 8,8(7,3-9.6); F=59%<br>overall F = 53%	reported with Tanner stage, any with pubertal develop-ment excluded.	D+E:<br>Community & Academic institution	3-mth intervention, low energy diet (1200-1400kcal), 3-5 meals every day, instructions concerning PA, 10-14 food day diary, 3-day food diary.	3:<br>0	BIA	WL: -0.98<br>No WL:-0.2	WL:-2.90<br>No WL:0.30
10	Garanty-Bogacka, 2011[42] Poland	Cohort:<br>Total = 50 (50)	BMI > 97th %ile (Polish ref pop.)	Age range:8-18<br>14.2 ± 2.6; F=58%	Yes	D+E:<br>Clinic (Hospital)	Exercise therapy (Instructions in PA + reducing sedentary behaviour) + Reduction in fat and sugar intake.	6:<br>0	Skinfold measurements & Lohman's formula	All:-1	All: -4.70
11	Grønbæk 2012[43] & Kazankov 2014[44] Denmark **Julemaerkehjemmet Hobro (same cohort)**	Cohort:<br>Total = 117 (117)<br>(n=71 attended 12 mth FU)	NR.<br>Obese. BL BMI-SDS: 2.93±0.52	Age range: NR<br>12.1 ± 1.3<br>F=56%	NR	D+E:<br>Community	Individually designed healthy diet + moderately strenuous PA program (at least 1hr/day).	2.5 months/10 weeks:<br>12mth FU	BIA	All: -0.63	All:-4.30
12	Hvidt 2014[45] Denmark	Cohort:<br>Total = 61 (61)	Children's Obesity Clinic; BMI > 90th %ile (Danish ref pop.) = z-score 1.28. BL BMI-SDS: 2.73±0.60	Age range:10-18<br>Median: 12.5<br>F=54%	NR	D+E:<br>Clinic (Hospital)	Family-centred approach involving behaviour changing techniques (90 advice and advice strategies on low-calorie diet + activity e.g. 10-20 items aimed to reduce obesity).	12:<br>0	BIA	All: -0.21	All: -3.40
13	Kirk, 2005[46] USA	Cohort:<br>Total = 177 (177)<br>Children (5-10yrs) = 85<br>Adolescents (11-19yrs) = 92	BMI > 95th %ile	Age range: 5-19<br>9.0 ± 1.5<br>Overall F=61%<br>Children: F = 24%<br>Adolescents: F = 59%	NR	D+E:<br>Clinic (Hospital)	Behavioural intervention with individualised behavioural goals for nutrition, PA & family support.	5:<br>6	DXA	GP1: -0.18<br>GP2: -0.13<br>All: -0.15	GP1:-2.10<br>GP2:-2.40<br>All:-2.20

For peer review only

For peer review only - http://bmjopen.bmj.com/site/about/guidelines.xhtml

14	Klijn 2007[47]<br>The Netherlands	Cohort:<br>Total = 15 (15)	BMI>30	Age range:10-18<br>14.7(2.1); F=NR	NR	E:<br>Community	Aerobic exercise training programme – 12 weeks; 3 x 30-60 min aerobic group sessions/week (2x gym/outdoors, 1 x swimming pool). P.E teacher led. Diverse indoor, outdoor and swimming activities.	3:<br>0	% body fat calculated by "dividing fat mass by total body mass"	All: -0.4	All: -3.80
15	Lazzer 2008[48]<br>Italy	Cohort:<br>Total = 19<br>Boys = 7 (7)<br>Girls = 12 (12)	BMI > 97th %ile	Age range: 8-12<br>Boys: 9.9 ±1.6<br>Girls:11.2 ± 1.5<br>Overall F=63%	Yes – Tanner	D+E:<br>Community	2 x 50min/wk endurance training + 2hr/wk PE lessons + 1 x wk child & parent dietetic class + 1 x wk psychological group class.	8:<br>12	DXA	Boys: -0.4<br>Girls: -0.2	Boys: -4.00<br>Girls:-2.20
16	Meyer 2006[49]<br>Germany	RCT:<br>Total = 67<br>IG=33 (33)<br>OC=34 (34)	BMI > 97th %ile (German paediatric population)	Age range: 11-16<br>IG: 13.7 ± 2.1; F=48%<br>OC: 14.1 ± 2.4; F =50%	Yes - Tanner	E:<br>Clinic (Hospital)	3 x exercise sessions (Monday: swimming and aqua aerobic training 60 min + Wednesday sports games 90 min + Friday walking 60 min)/ wk;<br>Control: Maintain current level of PA	6:<br>0	BIA	IG: -0.43<br>CG: -0.14	IG:-1.00<br>CG: 0.00
17	Miraglia 2015[50]<br>Brazil	Cohort:<br>Total = 27 (27)	BMI z-score > 2	Age range: 6-13<br>Median 10.3; F=48%	NR	D+E:<br>Clinic (Hospital)	AmO: Outpatient Ambulatory. Obesity outpatient clinic - lifestyle change based on goals agreed relative to feeding habits & physical exercise, followed mthly. 12 mths: Subjects assessed at inclusion & after 12 mths of FU to obtain anthropometric & adipokine measurements.	12:<br>0	BIA	All: -0.4	All: -0.10
18	Morell-Azanza 2016[51]<br>Rendo-Urteaga 2015[52]<br>Spain<br>**(same cohort)**	Cohort:<br>Total = 54 (40)<br>high responders =21<br>low responders = 19	OW/OB as per Cole *et al* 2000	Age range: 7-15<br>Mean =11<br>F=53% (of N analysed)	Yes – Tanner	D:<br>Clinic (Hospital)	Moderate energy-restricted diet + nutritional education sessions with dietitian + family involvement.	2.5:<br>0	BIA	HR:-0.79<br>LR: -0.18<br><br>HR: -0.64<br>LR: -0.07	HR:-3.10<br>LR: -0.60<br><br>HR: -2.49<br>LR: -0.37
19	Murer 2011[53]<br>Aeberli, 2010[54]<br>Switzerland<br>**(same cohort)**	Cohort:<br>Total = 206 (203)	BMI > 98th %ile	Age range:10-18<br>14.1 ± 1.9; F=44%	NR	D+E:<br>Clinic/hospital	Moderate caloric restriction.2 x 60-90min/day endurance exercise + 4-5 hr/wk. exercise session + behaviour modification.	2:<br>0	BIA	All: -0.42	All: -5.50
20	Murdolo 2017[55]<br>Italy	Cohort:<br>Total = 53(53)<br>Responders = 44<br>Non-responders = 9	NR	Age range: 5-13<br>Responders: 9.0 ± 1.1; F=50%<br>Non-responders: 2.09 ± 0.32; F=33%	Yes -Tanner	D+E:<br>Community	Educational Wt Excess Reduction Program	24:<br>>6 mths	BIA	Responders: -0.44<br>Non-responders: 0.11	Responders :-2.90<br>Non-responders: -2.00

For peer review only - http://bmjopen.bmj.com/site/about/guidelines.xhtml

21	Ning 2014[56] & BEAN 2011[57] USA **TEENS (same cohort)**	Cohort: Total = 145**(145)	BMI ≥ 95th %ile (CDC)	Age range: 11-18 13.1 F=65%	NR	D+E: Academic Institution	12 x 30 min nutritional session with adolescent and parent/s + Education/behavioural support sessions once every 2 wks, or alternating wks + PA 3 x 60 min/wk during initial 12 wks, then minimum of twice/wk.	6: 0	DXA	All: -0.1	All: -2.40
22	Pacifico 2013[58] Italy	Cohort: Total = 120 (120)	BMI > 95th %ile	Age range: (11.5-12.2) 11.9; F=35%	Yes (method ND)	D+E: Clinic (Hospital)	Hypocaloric diet (25-30 Kcal/kg/day) + 60 min/day ~ 5 days/wk moderate exercise + Reduce sedentary behaviour.	12: 0	NR	All: -0.32	All: -2.10
23	Racil 2013[59] Tunisia	RCT: Total = 34 HIT=11 (11) MIIT=11 (11) OC=12 (12)	BMI > 97th %ile (French standards)	Age range: NR HIIT: 15.6 ± 0.7 MIIT: 16.3 ± 0.52 OC:15.9 ±1.2 Overall F=100%	Yes -Tanner	D+E: Community	4-day diet records + HIIT or MIIT. Interval training program 3 x /wk on non-consecutive days.	3: 0	BIA	HIT: -0.4 MIT: -0.3 OC: 0	HIT: -2.90 MIT:-2.00 OC: -0.40
24	Racil 2016[60] Tunisia	RCT: Total = 47 HIIT =17 (17) MIIT16 (16) OC =14	BMI > 97th %ile (French standards)	Age range: NR 14.2 ± 1.2; F=100%	NR	E: Academic Institution	HIIT (Warm up + Interval training at 100%/50% MAS + Cooling down); MIIT (Warm up + Interval training 80%/50% MAS + Cooling down)	3: 0	BIA	HIT: -0.3 MIT: -0.3 OC: 0	HIT:- -3.90 MIT:-3.40 OC: -0.50
25	Reinehr 2004a[61] Germany **OBELDICKS**	Cohort: Total = 42 (42)	BMI ≥ 97th %ile	Age range: 6.1-15.1 10.2; F=57%	Yes - Tanner	D+E: Clinic (Hospital)	Obeldicks: Intensive phase 3 mths (Parents' course 2x/month + Behaviour therapy 2x/month + Nutritional course 2x/month + Exercise therapy 1x/wk) + Establishing phase 3 mths (Talk rounds for parents 1x/month + Psychological therapy + Exercise therapy 1x/wk) + Establishing phase 2 for 3 mths (Psychological therapy + Exercise therapy 1x/wk) + Establishing phase 3 for 3 mths (Exercise therapy 1x/wk).	12: 0	% body fat skinfold thickness	Sig. WL-0.9 NS WL: -0.2	Sig. WL:- 7.50 NS WL:- 3.00
26	Reinehr, 2008a, b[62,63] Germany **OBELDICKS**	Cohort: Ob + Sub. WL = 25 Ob + no change = 18 Normal control = 19 (BL data only)	IOTF using pop. -specific data	Ob: 10.8 ± 2.6; F=61% Lean C: 10.3±2.9; F=58% Ob + Sub. WL : F= 68% Ob + no change: F = 50%	Yes -Tanner	D+E: Clinic (Hospital)	Obeldicks	12: 0	% body fat skinfold thickness	WL: -0.6 No WL: -0.1	WL: -8.00 No WL:0.00

For peer review only

For peer review only - http://bmjopen.bmj.com/site/about/guidelines.xhtml

27	Rohrer 2008[64]<br>Germany<br>**Fit Kids**	Cohort:<br>Total = 22 (22)<br>Unchanged BMI= 12<br>Reduced BMI = 10	BMI > 99.5th %ile (German standard values) or BMI > 97th %ile with obesity-associated risk factors or BMI >90th %ile with obesity-associated disease	Age range: 7-15<br>Median: 11.9<br>F=27%<br>Unchanged BM: F = 33%<br>Reduced BMI: F=20%	NR	D+E:<br>Community	Physical exercise (2 x wk, 100 hrs in total) + Nutritional/heath education and psychological care for the child (x wk, 43.5 hrs total) and parent/s (2 x wk, 12 hrs total).	12:<br>0	BIA	Increased BMI: 0.12<br>Reduced BMI:-0.35	Increased BMI: 1.05<br>Reduced BMI:-0.05
28	Rolland-Cachera 2004[65]<br>France	RCT:<br>Total = 99<br>PROT- = 61 (53)<br>PROT+ =60 (46)	BMI > 97th %ile (French reference values)	Age range: 11-16<br>PROT- = 14.1 ± 1.2; F = 74%<br>PROT + =14.4 ± 1.3; F = 72%	NR	D+E:<br>Academic Institution	Wt reducing diet; 7hr/wk vigorous sports + 7hr/wk outdoor activities; advice on nutrition & PA during wkends/holidays.	9:<br>12 + 24	BIA	PROT- :-2.6<br>PROT+:-2.5	PROT- :-12.40<br>PROT+:-12.10
29	Roth 2016[66]<br>Germany<br>**OBELDICKS**	Cohort:<br>Total = 69<br>OB + WL = 32<br>OB + with WL = 37	OB as per IOTF criteria	NR – (see Obeldicks age range)<br>Ob with WL: 11.8 ± 2.0; F=50%<br>Ob without WL: 12.1 ± 2.1; F=51%<br>[Normal wt: 12.3 ± 3.0; F=45%]	Yes - Tanner	D+E:<br>Clinic (Hospital)	Obeldicks	12:<br>0	% body fat skinfold thickness	WL: -0.69<br>No WL: 0.03	WL: -9.60<br>No WL: -4.30
30	Savoye 2005[67]<br>USA<br>**Bright Bodies**	Cohort:<br>Total = 33 (25)<br>SMP=10 (8)<br>BFC = 23 (17)	BMI ≥ 95th %ile	Age range: 11-16<br>13.5 ± 0.3;<br>SMP:13.3 ±0.6; F=75%<br>BFC: 13.6 ±0.3; F= 65%	NR	D+E:<br>Academic Institution	Bright Bodies Weight Management Program: nutrition education, exercise, behavioural modification.<br>2 x 30 min exercise sessions + 1 x 45 min nutrition/behaviour medication group session per week. 4 levels: Beginner, Intermediate i, Intermediate ii, Advanced. All levels 12 weeks duration.<br>Mthly maintenance classes after 1 yr (support-group style)	12:<br>12	BIA	SMP: -0.36<br>BFC: -0.12	SMP:-6.50<br>BFC: -4.20

For peer review only

31	Savoye 2007, 2011[68,69]<br>USA<br>**Bright Bodies**<br>(data taken from 2011 paper)	RCT+ Long term FU results (cohort)<br>RCT Total = 174<br>BB=105<br>CC=69<br>1 YR ANALYSIS<br>BB = 75<br>CC=44	BMI ≥ 95th %ile (CDC)	Age range: 8-16<br>BB: 12.0 ± 2.5; F=56%<br>CC: 12.5 ± 2.3; F=68%	NR	D+E:<br>Academic Institution (local school).	Bright Bodies Weight Management Program: nutrition education, exercise, behavioural modification.2 x sessions/wk for 6 mths, then biweekly for next 6 mths. BB: 2x50 min exercise + 1x40 min nutrition/behaviour modification per wk + 12 mths no active intervention.<br>Control group: standard care – paed. obesity clinic (biannual clinic appt; diet + exercise counselling) Structured tx & teaching program (28 x 45 min therapeutic sessions e.g. PA, nutrition, healthy cooking)	12:<br>12<br><br>FU<br>1.5:<br>24	BIA	IG: -0.21<br>CG: 0.01	IG: -3.90<br>CG: 2.10
32	Savoye 2014[70]<br>USA<br>**Bright Bodies**	RCT<br>Total = 75<br>BB=38 (31)<br>CC =37 (27)	BMI ≥ 95th %ile	Age range:10-16<br>BB: 12.7 (1.9); F=68%<br>CC: 13.2 (1.8); F=62%	Yes-Tanner	D+E:<br>Academic Institution	Bright Bodies Weight Management Program: nutrition education, exercise, behavioural modification. 2 x 30 min exercise sessions + 1 x 45 min nutrition/behaviour medication group session per week. 4 levels: Beginner, Intermediate I, Intermediate ii, Advanced. All levels 12 weeks duration. Mthly maintenance classes after 1 yr (support-group style)	6:<br>0	BIA	BB: -0.05<br>CC: 0.04	BB: -3.30<br>CC: 0.40
33	Schiel 2016[71]<br>Germany	Cohort:<br>Total =143 (143)	BMI-SDS ≥97th %ile	Age range: NR<br>13.9 ± 2.4; F=62%	NR	D+E:<br>Clinic (Hospital)	Structured Tx & Teaching Program (STTP): 28 x 45 min therapeutic sessions e.g. PA, nutrition, healthy cooking	1.5:<br>24	NR	All: -0.26	All: -3.40
34	Seabra 2016[72]<br>Portugal	Cohort:<br>Total = 88<br>soccer =29 (29)<br>Trad. Act. =29 (29)<br>OC =30 (30)	BMI-SDS > 2	Age range: 8-12<br>Soccer: 10.5 ± 1.5<br>Trad. act: 11.0 ± 1.6<br>OC=10.0 ±1.3<br>Overall F=0%	Yes - Tanner	E:<br>Community	Soccer & trad. activity programmes (3 x 60-90min/wk) + 2 x 1hr at BL & 3 mths later energy balance session.	6:<br>0	DXA	Soccer: -0.2<br>Trad.: -0.2<br>CG: -0.1	Soccer:-2.20<br>Trad:-4.10<br>CG:3.10
35	Truby 2016[73]<br>Australia	RCT:<br>Total = 87<br>SMC =37 (33)<br>SLF=36 (32)<br>WList OC =14 (14)	BMI > 90th %ile (CDC)	Age range: 10-17<br>SMC: 13.2 ± 1.9; F=73%<br>SLF: 13.2 ± 2.1; F=72%<br>WList OC: 13.6 ±1.9; F=71%	Yes -Tanner	D:<br>Clinic (Hospital)	Structured modified CHO diet (35% CHO; 30% protein; 35% fat), structured low-fat diet (55% CHO; 20% protein; 25% fat), Control (no dietary advice).	3:<br>0	BIA	SLF: -0.09<br>SMC:-0.15<br>CG: 0.02	SLF: -0.13<br>SMC: -0.40<br>CG: 2.62

For peer review only - http://bmjopen.bmj.com/site/about/guidelines.xhtml

36	Van der Baan-Slootweg 2014[74] Netherlands	RCT: Total = 90 Inpt. = 45 (37) AmO = 45 (36)	BMI z score ≥ 3.0 or > 2.3 with OB-related health problems	Age range: 8-18 Inpt: 13.8 ± 2.3; F=58% AmO: 13.9 ± 2.5; F=58%	NR	D+E: Clinic (Hospital)	Inpt. (Hospitalised 26 wks on working days - 4 days/wk 30-60min exercise + nutrition/BM once/wk + parents/caregivers 3 x 1hr lesson on nutrition/BM); Ambulatory (12 visits at increasing time intervals - 1 hr exercise session + encouraged 3 x exercise/wk + 1 hr educational programme + 30 min nutrition education).	6: 24	BIA	InpT: -0.6 AmO: -0.35	InP: -3.34 AmO:-7.87
37	Visuthranukul 2015[75] Thailand	RCT: Total = 70 (52) I =35(25) OC=35 (27)	ND. BL BMI z-score: I = 3.7 ±0.9 C = 3.6±1.6	Age range: 9-16 I = 11.9 ± 1.9; F=36% C = 12.0 ± 2.1; F=30%	Yes -Tanner	D: Clinic (Hospital)	I (Low GI diet + Energy restriction 1400-1500 kcal/day + Increased exercise); OC (Energy restriction 1200-1300 kcal/day + Low fat/high fibre diet + Increased exercise).	6: 0	BIA	IG:-0.3 CG: -0.3	IG:0.10 CG:0.10
38	Vitola 2009[76] USA	Cohort: Total = 8(7)	BMI ≥ 95th %ile	Age range: NR 15.3± 0.6; F=12.8%	Yes -Tanner	D+E: Clinic (Hospital)	Individual behavioural therapy sessions with psychologist. Parents involvement encouraged. Self-monitoring of PA & food intake. Gradual reduction of caloric intake to ≈1200-1500 kcal/day. Ongoing therapy - wt loss therapy repeated when 5% body wt lost & wt stable for at least 4 wks	NR	DXA	All: -0.3	All: -5.30
39	Wickham, 2009[77] & Evans, 2009[78] USA **TEENS (same cohort)**	Cohort: Total = 168 (64) * Completers only =57	BMI ≥ 95th %ile (CDC)	Age range: 11-18 13.9 ± 1.9; F=62%	NR	D+E: Academic Institution	Exercise 1 day/wk at facility + 2 additional exercise days at facility of ppts' choice + 30 min/wk nutrition education/behavioural support sessions.	6: 0	BIA	Completers: -0.07	Completers :-1.30

KEY: %ile = percentile; AmO = outpatient ambulatory; An. = analysed; apt. = appointment; BB = Bright Bodies; BIA = bioelectrical impedance analysis; BFC = better food choices; BL = baseline; BM = behaviour modification; BMI = body mass index; C = control; CG: control group; CBT = cognitive behavioural therapy; CDC = Centre for Disease Control; CG = control group; CHO = carbohydrate; D = diet; DXA = Dual-energy X-ray absorption; E = exercise; FBBT = family-based behavioural treatment; F = female; FU = follow up; GI = glycaemic index; GT = group therapy; HGI = high glycaemic index; hr = hour; HZ = heterozygous; HO = homozygous; ht = height; I = intervention; IG= intervention group; IOTF = International Obesity Task Force; Inpt. = inpatient; LGI = low glycaemic index; LMS = least-mean-squares; LS = long stay; min= minute; mth = month; MO = morbidly obese; norm. normal; n = number; NAFLD = Non-alcoholic fatty liver disease; ND = not described; NR = not reported; OB = obese; OC = obese control; OW = overweight; paed. = paediatric; PA = physical activity; PE = physical activity; PROT = protein; RCT = randomised controlled trial; SD = standard deviation; SDS = standard deviation score; SMP = structured meal plan; SS = short stay; Sub. = substantial; SMC = structured modified carbohydrate diet; trad. = traditional; Trad. act = traditional activity; tx = treatment; wk = week; WList OC = wait list obese control; WL = weight loss; wt = weight; X-over = crossover; yr = year

*studies with change in % body fat included in the analysis
**minor discrepancies in reporting of data in papers

FOOTNOTE: For studies reported in multiple publications, the reference that provided the most comprehensive information has been used *(thus Ning et al 2014[56] includes data from Bean et al 2011[57]; Evans et al 2009[78] is reported under Wickham et al 2009[77], Aeberli et al 2010[54] is reported under Murer et al 2011[53]; Rendo-Urtega et al 2015[52] is reported under Morell-Azanza et al[51] and Kazankov et al 2014[44] is reported under Grønbæk et al* 2012[43]).

For peer review only - http://bmjopen.bmj.com/site/about/guidelines.xhtml

Narrative description of studies that reported BMI-SDS and percentage body fat

Of the 39 studies that reported percentage body fat included in our analysis, seven were conducted in both Germany and USA, four in Italy, followed by Australia (n=2), Denmark (n=2), Netherlands (n=2), Poland (n=2), Switzerland (n=2), Tunisia (n=2) and one each in Belgium, Brazil, Canada, Chile, France, Portugal, Spain, Thailand and the UK. There were country-specific variations in the definition of obesity, with most studies defining obesity by participants having a BMI-SDS > 2, or a BMI percentile of at least > 90th percentile. Most of studies utilised a cohort design (*n* = 27), 11 were RCTs, of which one included results from a cohort of the original RCT. There was also one study which adopted a quasi-randomised design.

Most studies (n=20) conducted their intervention in the hospital clinic setting. Eight studies conducted the intervention in the community setting and ten in academic institutions. One conducted the intervention in a mixed setting, reporting use of both a community setting and academic institution.

Twenty-eight studies conducted interventions that comprised both diet and exercise components. The remaining studies (n=11) utilised interventions that focused either on exercise or diet only. The duration of the interventions ranged from 15 days to 24 months. The majority of studies (n=29; 74%) did not report any follow-up after the lifestyle treatment intervention. The duration of follow-up in the studies where it was conducted and reported, ranged from 6 months to 2 years.

The sample sizes of the included studies ranged from 8 to 203 participants. The age of the participants ranged from 4 to 19 years. Studies predominantly had a mix of males and females (95%) with only three studies specifically focused on either only girls[59,60] or boys[72]. Seventeen

For peer review only - http://bmjopen.bmj.com/site/about/guidelines.xhtml

studies (44%) measured pubertal development of participants according to Marshall and Tanner staging, with pubertal status categorised into three groups: prepubertal, pubertal, and late/post-pubertal[79]. Four studies (10%) reported that pubertal development was measured but the methodology was not defined. Eighteen studies (46%) did not report any measures of pubertal development.

Quality Assessment

The quality of the conduct of each study was assessed using the same criteria as the HTA systematic review of the long-term effects and economic consequences of treatments for obesity and implications for health improvement[30]. The results of the quality assessment can be found in Table 2. In summary, none of the 39 studies that reported percentage body fat were considered to be of poor quality, 21 studies (54%) were rated as being of moderate quality and 18 studies (46%) achieved a score over 81% indicating high quality.

For peer review only

For peer review only - http://bmjopen.bmj.com/site/about/guidelines.xhtml

Table 2: Quality Assessment of included studies

		SAMPLE						CONDUCT OF STUDY				FOLLOW-UP			ANALYSIS				INTERPRETATION				
	Author, Year,	1.Aims clearly stared	2. sample size justified	3. Age of participant defined	4. Measurements at start clearly stated?	5. Measurements likely to be valid and reliable?	6. Risk factors recorded clearly?	7. Was the intervention before follow -up defined?	8. Setting of the study clear?	9. Is mode of assessment described?	10. Did untoward events occur during the study?	11. Was there a follow up?	12. Was follow up necessary?	13. Are losses to follow up defined?	14. Was basic data adequately described?	15. Do numbers add up?	16. Did analysis allow for passage of time?	17. Was statistical significance assessed?	18. Were the main findings interpreted adequately?	19. Were null/negative findings interpreted?	20. Are important effects overlooked?	Total (x/40)	Overall rating
1	Bell 2007[32]	Yes	Yes	Yes	Yes	Yes	No	Yes	?	Yes	No	No	Yes	Yes	Yes	Yes	Yes	Yes	Yes	Yes	No	**35**	**87.5**
2	Bock 2014[33]	Yes	No	Yes	Yes	Yes	No	Yes	Yes	Yes	No	Yes	Yes	Yes	Yes	Yes	Yes	Yes	Yes	Yes	No	**36**	**90**
3	Bruyndonckz 2015[34]	Yes	No	Yes	Yes	Yes	No	Yes	Yes	Yes	No	Yes	Yes	Yes	Yes	Yes	Yes	Yes	Yes	Yes	No	**36**	**90**
4	Bustos 2015[35]	Yes	No	Yes	Yes	Yes	No	Yes	Yes	Yes	No	No	Yes	No	Yes	Yes	Yes	Yes	Yes	No	No	**30**	**75**
5	Calcaterra 2013[36]	Yes	No	Yes	Yes	Yes	No	Yes	Yes	Yes	No	No	Yes	No	Yes	Yes	Yes	Yes	Yes	?	No	**31**	**77.5**
6	Dobe 2011[37]	?	No	Yes	?	?	No	Yes	?	?	No	No	Yes	No	Yes	Yes	Yes	Yes	Yes	?	No	**26**	**65**
7	Farpour-Lambert, 2009[38]	Yes	Yes	Yes	Yes	Yes	No	Yes	?	Yes	No	Yes	Yes	Yes	Yes	Yes	Yes	Yes	Yes	Yes	No	**37**	**92.5**
8	Ford, 2010a[39], 2010b [40]	Yes	Yes	Yes	Yes	Yes	No	Yes	Yes	Yes	?	Yes	Yes	Yes	Yes	Yes	Yes	Yes	Yes	Yes	?	**35**	**87.5**
9	Gajewska, 2016[41]	Yes	No	Yes	Yes	Yes	No	Yes	Yes	Yes	No	No	Yes	No	Yes	Yes	Yes	Yes	Yes	?	No	**31**	**77.5**
10	Garanty-bogacka, 2011[42]	Yes	No	Yes	Yes	Yes	?	?	?	Yes	?	No	?	No	Yes	?	Yes	Yes	Yes	?	No	**26**	**65**
11	Gronbaek 2012[43]; Kazankov 2014[44]	Yes	?	Yes	Yes	Yes	No	Yes	Yes	Yes	No	Yes	Yes	Yes	Yes	Yes	Yes	Yes	Yes	Yes	No	**37**	**92.5**
12	Hvidt 2014[45]	Yes	No	Yes	Yes	Yes	No	Yes	Yes	Yes	No	Yes	Yes	Yes	Yes	Yes	Yes	Yes	Yes	Yes	?	**34**	**85**
13	Kirk 2005[46]	Yes	No	Yes	Yes	Yes	No	Yes	Yes	Yes	?	No	Yes	?	Yes	Yes	Yes	Yes	Yes	?	?	**29**	**72.5**
14	Klijn 2007[47]	Yes	No	Yes	Yes	Yes	No	Yes	?	Yes	No	No	Yes	No	No	Yes	Yes	Yes	Yes	No	No	**27**	**67.5**
15	Lazzer 2008[48]	Yes	No	Yes	Yes	Yes	No	Yes	Yes	Yes	No	Yes	Yes	No	Yes	Yes	Yes	Yes	Yes	No	No	**32**	**80**
16	Meyer 2006[49]	Yes	No	Yes	Yes	Yes	No	No	Yes	Yes	No	No	Yes	No	Yes	Yes	Yes	Yes	Yes	Yes	No	**30**	**75**
17	Miraglia 2015[50]	Yes	No	No	Yes	Yes	No	Yes	?	Yes	No	No	Yes	No	?	Yes	Yes	Yes	?	Yes	Yes	**25**	**62.5**

For peer review only - http://bmjopen.bmj.com/site/about/guidelines.xhtml

18	Morell-Azanza 2016[51]; Rendo-Urteaga 2015[52]	Yes	Yes	Yes	Yes	Yes	No	Yes	Yes	Yes	No	Yes	Yes	No	Yes	Yes	Yes	Yes	Yes	No	No	**32**	**80**
19	Murer 2011[53]; Aeberli 2010[54]	Yes	Yes	Yes	Yes	Yes	No	Yes	Yes	Yes	No	Yes	Yes	Yes	Yes	Yes	Yes	Yes	Yes	Yes	No	**38**	**92**
20	Murdolo 2017[55]	Yes	No	Yes	Yes	Yes	No	No	No	Yes	No	Yes	Yes	No	Yes	Yes	Yes	Yes	Yes	No	No	**28**	**70**
21	Ning 2014[56]; Bean 2011[57]	Yes	No	Yes	Yes	Yes	No	Yes	Yes	Yes	No	Yes	Yes	Yes	Yes	Yes	Yes	Yes	Yes	No	No	**34**	**85**
22	Pacifico 2013[58]	Yes	No	Yes	Yes	Yes	No	Yes	Yes	Yes	No	Yes	Yes	No	Yes	Yes	Yes	Yes	Yes	No	?	**31**	**77.5**
23	Racil 2013[59]	Yes	No	Yes	Yes	Yes	No	Yes	?	Yes	No	No	?	No	Yes	Yes	Yes	Yes	Yes	Yes	?	**29**	**72.5**
24	Racil 2016[60]	Yes	No	?	Yes	Yes	No	Yes	?	Yes	No	No	Yes	No	Yes	Yes	Yes	Yes	Yes	No	No	**28**	**70**
25	Reinehr 2004a[61]	Yes	No	Yes	Yes	Yes	No	Yes	Yes	Yes	No	No	Yes	No	Yes	Yes	Yes	Yes	Yes	No	?	**29**	**72.5**
26	Reinehr 2008a[62], 2008b[63]	Yes	No	Yes	Yes	Yes	No	Yes	Yes	Yes	No	Yes	Yes	No	Yes	Yes	Yes	Yes	Yes	Yes	No	**32**	**80**
27	Rohrer 2008[64]	Yes	No	Yes	Yes	Yes	No	Yes	Yes	Yes	No	Yes	Yes	No	Yes	Yes	Yes	Yes	Yes	?	No	**33**	**82.5**
28	Rolland-Cachera 2004[65]	Yes	No	Yes	Yes	Yes	No	Yes	Yes	Yes	No	Yes	Yes	Yes	?	Yes	Yes	Yes	Yes	No	No	**33**	**82.5**
29	Roth 2016[66]	Yes	No	Yes	Yes	Yes	No	Yes	No	Yes	No	No	Yes	No	Yes	Yes	Yes	Yes	Yes	No	No	**28**	**70**
30	Savoye 2005[67]	Yes	No	Yes	Yes	Yes	No	Yes	Yes	Yes	No	Yes	Yes	Yes	Yes	Yes	Yes	Yes	Yes	No	No	**34**	**85**
31	Savoye 2007[68],2011[69]	Yes	Yes	Yes	Yes	Yes	No	Yes	Yes	Yes	No	Yes	Yes	Yes	Yes	Yes	Yes	Yes	Yes	No	No	**36**	**90**
32	Savoye 2014[70]	Yes	Yes	Yes	Yes	Yes	No	Yes	?	Yes	No	No	Yes	Yes	Yes	Yes	Yes	Yes	Yes	Yes	No	**35**	**87.5**
33	Schiel 2016[71]	Yes	No	?	Yes	Yes	No	Yes	Yes	Yes	No	Yes	Yes	Yes	Yes	Yes	?	Yes	No	?	Yes	**29**	**72.5**
34	Seabra 2016[72]	Yes	Yes	Yes	Yes	Yes	No	Yes	Yes	Yes	No	No	Yes	Yes	Yes	Yes	Yes	Yes	Yes	No	No	**34**	**85**
35	Truby 2016[73]	Yes	Yes	Yes	Yes	Yes	No	Yes	Yes	Yes	No	Yes	Yes	Yes	Yes	Yes	Yes	Yes	Yes	Yes	No	**38**	**95**
36	Van der Baan-Slootweg 2014[74]	Yes	Yes	Yes	Yes	Yes	No	Yes	Yes	Yes	No	Yes	Yes	Yes	Yes	Yes	Yes	Yes	Yes	No	No	**36**	**90**
37	Visuthranukul 2015[75]	Yes	Yes	Yes	Yes	Yes	No	Yes	Yes	Yes	No	Yes	Yes	Yes	Yes	Yes	Yes	Yes	Yes	Yes	No	**38**	**95**
38	Vitola 2009[76]	Yes	No	Yes	Yes	Yes	No	?	?	Yes	No	No	Yes	No	Yes	Yes	Yes	Yes	Yes	No	No	**28**	**70**
39	Wickham 2009[77]; Evans 2009[78]	Yes	No	Yes	Yes	Yes	No	Yes	Yes	Yes	No	Yes	Yes	?	Yes	Yes	Yes	Yes	No	Yes	?	**30**	**75**

DECISION KEY:

? = Unclear

For Q6. Were risk factors clearly recorded? We said "No" rather than "unclear" to all the studies that didn't record risk factors;

For Q10. Did untoward events occur during the study? We said "No" rather than unclear if not mentioned.

Rating: Not satisfactory 1-50%; Moderate quality = 51-80%; High quality = 81%

For peer review only

Quantitative Analysis

From the 39 studies we identified all data subsets that reported a mean change in BMI-SDS, an associated mean change in percentage body fat (or pre- and post- study values from which these could be calculated) and the number of cases analysed. A few studies yielded only aggregated data for the whole study. For the others, typical data subsets included intervention vs control, male vs female or good vs poor responders (see Table 1), and these were used in preference to aggregated results if both were available. In all, there were 66 subsets, with numbers analysed totalling 2,618.

SEs were required for the mean changes in percentage body fat and, if not given explicitly, were calculated, from either the standard deviations (SDs) or the 95% confidence intervals (CIs) of the mean changes. In total, 22 data sets had SEs. For the remainder, the SEs were estimated from the SDs associated with the baseline and the post-intervention percentage body fat values, making an assumption about the degree of correlation between them. The median and interquartile range (IQR) of the correlation coefficients estimated from the 9 data sets where both the SEs of mean change and the SDs for baseline and post intervention percentage body fat values were available was 0.81 (IQR 0.59-0.82) and 0.81 has been used in the following analysis. A small number of data sets (n=6)[34,41,61] only had medians and IQRs (or range) reported for the baseline and post intervention results; the mean and SDs were estimated from them[80].

The meta-regression line was fitted and plotted together with the 95% prediction intervals for the change in percentage body fat across the study data sets. The smallest reduction of mean BMI-SDS associated with a reduction in mean percentage body fat was determined as the smallest reduction in mean BMI-SDS with an associated 95% prediction interval wholly below zero.

For peer review only - http://bmjopen.bmj.com/site/about/guidelines.xhtml

A series of sensitivity analyses were conducted. Sensitivity Analysis 5i: using the 22 cases where the SEs of the mean change in percentage body fat were actually known, Sensitivity Analysis 5ii: omission of 2 extreme values and Sensitivity Analysis 5iii: assuming a correlation of 0.50 instead of 0.81. In further exploratory analyses, the percentage of girls and the length of the study (baseline to end of intervention) were added to see if these affected the prediction of mean change in percentage body fat.

Results from the quantitative analysis

Figure 3 shows the results of the analysis and the fitted regression line. The circles represent the study results (i.e. the mean changes in percentage body fat and mean changes in BMI-SDS) analysed for each study, with the size of the circles representing the precision of the mean change in percentage body fat, i.e. the reciprocal of the SE squared).

Figure 3: Meta-regression line showing the relationship between mean change in percentage Body Fat and BMI-SDS across the 39 studies (66 subsets) analysed

The fitted regression line shown in Figure 3 is:

Mean change in percentage body fat = 5.179 x Mean change in BMI-SDS - 0.767.

The regression slope was statistically significant ($P<0.001$), confirming a relationship between the mean loss of percentage body fat and the mean change in BMI-SDS across the data subsets; the proportion of the between-subset variance explained by the mean change in BMI-SDS (i.e. ‘a type of adjusted R-squared’) was 68%. There was, however, significant between-subset heterogeneity with 89% of the percentage of the total residual variance attributable to this, (i.e. I^2). It was further noted that when added to the model, neither the percentage girls in the study

For peer review only

For peer review only - http://bmjopen.bmj.com/site/about/guidelines.xhtml

sets nor the durations of the interventions significantly improved the prediction of mean change in percentage body fat from the mean change in BMI-SDS (P=0.36, P=0.89 respectively).

Figure 3 also shows the 95% prediction intervals for the mean change in percentage body fat. The upper limit of the prediction interval was below 0 only when the mean reduction in BMI-SDS was greater than 0.6, suggesting that any new study should aim to reduce the BMI-SDS by at least this amount to be confident of achieving a mean reduction of percentage body fat.

A normal plot for the standardised predicted random effects is shown in Figure 4. Most were within +/-2 although the data sets themselves were not wholly independent (as some came from the same studies).

Figure 4: Normal plot for the standardised predicted random effects from the meta-regression

None of the sensitivity analyses conducted (Figure 5) significantly altered the findings, namely that a mean change of 0.6 or more in BMI-SDS was associated with a definitive mean loss in percentage body fat. In Figure 5(ii), with the exclusion of the two extreme data points, the linear trend can be seen more clearly across the range of mean BMI-SDS losses.

Figure 5: Sensitivity analysis

For peer review only - http://bmjopen.bmj.com/site/about/guidelines.xhtml

DISCUSSION

Summary of main results

This is the first of a series of papers that report on studies identified in a large systematic review. The objective of this paper was to attempt to establish the minimum change in BMI-SDS needed to achieve improvements in body fat in obese children and adolescents; BMI-SDS being by far the most frequently reported outcome in terms of weight management trial interventions in childhood. Seventy-three of the 90 included studies reported adiposity measures, but in our meta-regression only percentage body fat can be used as a reliable, comparable marker of change of adiposity. Thus, the analyses presented in this paper were conducted using data from 39 studies. All of the included studies were considered to be of moderate to high quality according to the HTA quality assessment tool[30]. Despite there being a positive relationship between mean change in percentage body fat and mean change in BMI-SDS, our modelling suggested that, in order to be confident of effecting a mean loss in percentage body fat, any future study should aim to reduce the BMI-SDS by at least 0.6.

Strengths and limitations

We believe that this is the first paper to attempt to bring together all studies that have reported both a change in BMI-SDS and changes in a marker of adiposity in the obese paediatric population. The systematic methods employed to identify the included studies were stringent, but it is possible that some relevant studies might have been missed. In addition, there was some variation in the reporting of results where there were multiple publications of the same study; in these cases, the results from the most comprehensive paper have been used. An important limitation to address in the broader context going forward, is whether BMI-SDS is the best way to represent changes in BMI at extremes of body weight. The US Center for

For peer review only - http://bmjopen.bmj.com/site/about/guidelines.xhtml

Disease Control cautioned the use of BMI-SDS in weight extremes in 2009[81]. Freedman *et al* have suggested that there are better measures of adiposity in severe obesity, such as percentage of 95th percentile BMI (%BMI^{p95}) or distance in Kg/m^2 from the 95th percentile (Δ BMI^{p95})[82]. Other groups have identified alternate methods when dealing with extremes of obesity such as BMI%[83] or percentage above IOTF-25[84]. Vanderwell *et al* have also suggested that BMI-SDS is only a weak to moderate predictor of percentage body fat in children especially under 9 years of age[85]. Notwithstanding these cautions, we based this analysis on the data available to us which was almost entirely reported in terms of BMI-SDS and continues to be the case in most recent publications to date.

It has been suggested that the relationship between change in percentage body fat and change in BMI-SDS may differ between very young and older children[86]. Our inclusion criteria stipulated ages from 4 to 19 years. Most of the studies spanned a wide range of ages (see Table 1) and we did not have access to individual child data to facilitate stratification by age. Data from four subsets of children up to 10 years[37,41,46,] however, did not suggest a different relationship from the whole cohort (Appendix 3).

Agreements and disagreements with other research

Previous research has shown that an improvement in body composition and cardiometabolic risk can be achieved with a BMI-SDS reduction of greater than or equal to 0.25 in obese adolescents, with greater benefits achieved when losing at least 0.5 BMI-SDS[39].

In clinical practice, the degree of weight loss with lifestyle intervention is moderate and the success rate 2 years after onset of an intervention is low (<20% with a decrease in BMI-SDS <0.25)[87]. There have been numerous reports of lifestyle-based weight management

For peer review only - http://bmjopen.bmj.com/site/about/guidelines.xhtml

interventions for obese children, many documenting changes in BMI-SDS, but a recent meta-analysis has documented that whilst such changes may be statistically significant, they are unlikely to lead to clinical improvements in metabolic health [88,89]. To our knowledge this is the first paper to establish the minimum change in BMI-SDS required to be certain of improving adiposity as percentage body fat for obese children and adolescents in clinical trials.

Clinical implications

If reducing fat mass is the aim of weight management interventions, our analysis in this review demonstrates that BMI-SDS changes must be of an order seldom achieved in trials worldwide. From our model, to be confident about ensuring an improvement in mean body fat, one should aim to reduce mean BMI-SDS by at least 0.6. Figure 3 and Sensitivity Analysis 5ii (Figure 5) suggest that to reduce body fat by 5% requires a much larger BMI-SDS reduction, of the order of 1.3 to 1.5, although there was a paucity of data in this region.

Recommendations for future research

Whilst we are undertaking further analyses looking at key cardiovascular and metabolic outcomes in childhood obesity that may demonstrate improvements at lesser levels of BMI-SDS reduction, the evidence suggests that very few childhood weight management trials to date are likely to have improved percentage body fat and calls in to question their overall efficacy in terms of health improvement. That said, any trial demonstrating an improvement of the magnitude of 0.6 BMI-SDS might be termed successful with a likely reduction in fat mass. However, given the mounting evidence that BMI-SDS may not accurately reflect adiposity at extremes of obesity, it seems prudent for future trials to report additional indices of derived BMI values which may better reflect changes in actual adiposity. Which of the many measures

For peer review only - http://bmjopen.bmj.com/site/about/guidelines.xhtml

suggested eventually establishes itself as the 'optimal' determinant at extremes of body mass is yet to be determined?

Conclusions

Using our model, to predict any fat mass improvement when reporting a weight management trial outcome requires a BMI-SDS decrease of 0.6. When evaluating key outcomes for future weight management trials and services, this figure needs to be borne in mind by researchers, health care professionals and commissioners when assessing apparent success.

Author Contributions

Ms Birch and Dr Perry provided substantial contributions to the conception and design of the study, designed the data extraction instrument, performed electronic database searches, data screening, extraction and quality assessment, coordinated and supervised data collection and drafted and revised the manuscript.

Professor Hamilton-Shield provided a substantial contribution to the conception and design of the study, conducted data screening and interpretation, and assisted with drafting and revision of the manuscript.

Dr Hunt provided statistical expertise in relation to study design and conducted the data analyses and contributed to the drafting and revision of the manuscript.

Mr Matson, Ms Chong and Ms Beynon were involved in data acquisition and management.

All authors approved the final manuscript as submitted and agree to be accountable for all aspects of the work.

For peer review only - http://bmjopen.bmj.com/site/about/guidelines.xhtml

Figure 1: Flow diagram from the systematic review that identified the included studies

Figure 2: Venn diagram illustrating the markers of metabolic health measured

Table 1: Characteristics of studies reporting adiposity outcomes with results of mean change in BMI-SDS and percentage body fat

Table 2: Quality Assessment of included studies

Figure 3: Meta-regression line showing the relationship between mean change in percentage Body Fat and BMI-SDS across the 39 studies (66 subsets) analysed

Figure 4: Normal plot for the standardised predicted random effects from the meta-regression

Figure 5: Sensitivity Analyses

For peer review only

References

1. Organization, WHO, Report of the commission on ending childhood obesity. 2016: World Health Organization.

2. National Child Measurement Programme - England, 2017-18 [National Statistics]. In: National Child Measurement Programme data source: Health and Social Care Information Centre; 2018. Content.digital.nhs.uk. (n.d.). National Child Measurement Programme - NHS Digital. [online] Available at: http://content.digital.nhs.uk/ncmp.

3. National Child Measurement Programme - England, 2016-17 [National Statistics]. In: National Child Measurement Programme data source: Health and Social Care Information Centre; 2018. Content.digital.nhs.uk. (n.d.). National Child Measurement Programme - NHS Digital. [online] Available at: http://content.digital.nhs.uk/ncmp.

4. Simmonds M, Llewellyn A, Owen CG, Woolacott N. Predicting adult obesity from childhood obesity: a systematic review and meta-analysis. *Obesity Reviews.* 2016;17(2): 95-107

5. Hannon TS, Rao G, Arslanian SA. Childhood Obesity and Type 2 Diabetes Mellitus. *Pediatrics.* 2005;116(2):473-480

6. Haines L, Wan KC, Lynn R, Barrett TG, Shield JP. Rising Incidence of Type 2 Diabetes in Children in the U.K. *Diabetes Care*. 2007;30(5):1097-1101

7. Urbina EM, Kimball TR, McCoy CE, et al. Youth with Obesity and Obesity-Related Type 2 Diabetes Mellitus Demonstrate Abnormalities in Carotid Structure and Function. *Circulation.* 2009;119(22):2913-2919

8. Pires A, Martins P, Pereira AP, et al. Insulin Resistance, Dyslipidemia and Cardiovascular Changes in a Group of Obese Children. *Arquivos Brasileiros de Cardiologia.,* 2015;104:266-273

9. Cook S, Kavey REW. Dyslipidemia and Pediatric Obesity. *Pediatric Clinics of North America* 2011;58(6):1363-1373

10. Chiarelli F, Marcovecchio ML. Insulin resistance and obesity in childhood. *European Journal of Endocrinology.* 2008;159(suppl 1): S67-S74

11. Tounian P, Aggoun Y, Dubern B, et al. Presence of increased stiffness of the common carotid artery and endothelial dysfunction in severely obese children: a prospective study. *The Lancet.* 2001;358(9291): 1400-1404

12. Klein S, Burke LE, Bray GA, et al. Clinical Implications of Obesity with Specific Focus on Cardiovascular Disease. A Statement for Professionals from the American Heart Association Council on Nutrition, Physical Activity, and Metabolism: Endorsed by the American College of Cardiology Foundation. *Circulation.* 2004;110(18):2952-2967

For peer review only - http://bmjopen.bmj.com/site/about/guidelines.xhtml

13. Krebs JD, Evans S, Cooney L, et al. Changes in risk factors for cardiovascular disease with body fat loss in obese women. *Diabetes, Obesity and Metabolism.* 2002;4(6):379-387

14. Goldstein DJ. Beneficial health effects of modest weight loss. *International journal of obesity and related metabolic disorders.* 1992;16(6):397-415

15. Colditz GA, Willett WC, Rotnitzky A, Manson JE. Weight gain as a risk factor for clinical diabetes mellitus in women. *Annals of Internal Medicine.* 1995;122(7):481-486

16. National Heart, Lung, and Blood Institute. "Clinical guidelines on the identification, evaluation, and treatment of overweight and obesity in adults. Bethesda (MD): National Heart." *Lung, and Blood Institute*; 1998

17. Cole TJ. The LMS method for constructing normalized growth standards. *European Journal of Clinical Nutrition.* 1990; 44:45–60

18. Ho M, Garnett SP, Baur L, et al. Effectiveness of Lifestyle Interventions in Child Obesity: Systematic Review with a Meta-analysis. *Pediatrics.* 2012;130(6): e1647-1671

19. Colquitt JL, Loveman E, O'Malley C, et al. Diet, physical activity, and behavioural interventions for the treatment of overweight or obesity in preschool children up to the age of 6 years. *Cochrane Database Syst Rev*, 2016. **3**: p. Cd012105

20. Oude Luttikhuis H, Baur L, Jansen H, et al., Cochrane review: Interventions for treating obesity in children. *Evidence*‐*based child health: A Cochrane Review Journal.* 2009;4(4):1571-1729

21. National Institute for Health and Care Excellence. Quality Standard [QS94]. Obesity in children and young people: prevention and lifestyle weight management programmes. *NICE*. 2015; https://www.nice.org.uk/guidance/qs94

22. Friedemann C, Mahtani K, Thompson M, Perera R. Cardiovascular disease risk in healthy children and its association with body mass index: systematic review and meta-analysis. *British Medical Journal.* 2012; 345: e4759

23. Prentice AM, Jebb SA. Beyond body mass index. *Obesity Review*. 2001; 2(3):141-7

24. Cole TJ, Freeman JV, Preece MA. Body mass index reference curves for the UK, 1990. *Archives of Disease in Childhood*. 1995;73(1):25-29

25. Kuczmarski RJ, Ogden CL, Grummer-Strawn LM, et al. CDC growth charts: United States. Advance Data, 2000;(314):1-27

26. Cole TJ, Bellizzi MC, Flegal KM, Dietz WH. Establishing a standard definition for child overweight and obesity worldwide: international survey. *British Medical Journal*. 2000; 320(7244):1240

For peer review only

For peer review only - http://bmjopen.bmj.com/site/about/guidelines.xhtml

27. De Onis M, Garza C, Onyango AW, Borghi E. Comparison of the WHO child growth standards and the CDC 2000 growth charts. *The Journal of Nutrition*. 2007;137(1):144-148

28. De Onis MD, Onyango AW, Borghi E, Siyam A, Nishida C, Siekmann J. Development of a WHO growth reference for school-aged children and adolescents. Bulletin of the World Health Organization. 2007b; 85(9):660-667

29. Higgins J, Green S. Cochrane handbook for systematic reviews of interventions. Chichester: Wiley; 2011

30. Avenell A, Broom J, Brown TJ. Systematic review of the long-term effects and economic consequences of treatments for obesity and implications for health improvement. *Health Technol. Assess.* 2004; 8(21):iii-iv:1-182.

31. Harbord RM, Higgins, JPT. 'Meta-regression in Stata.' *The Stata Journal.* 2008;8(4):493-519

32. Bell L, Watts K, Siafarikas A, et al. Exercise alone reduces insulin resistance in obese children independently of changes in body composition. *Journal of Clinical Endocrinol. Metab.* 2007; 92(11):4230-4235

33. Bock DE, Watts K, Siafarikas A, et al. The Health Initiative Program for Kids (HIP Kids): effects of a 1-year multidisciplinary lifestyle intervention on adiposity and quality of life in obese children and adolescents - a longitudinal pilot intervention study. *BMC Pediatrics.* 2014; 14:296. doi: 10.1186/s12887-014-0296-1

34. Bruyndonckx L, Hoymans VY, De Guchtenaere A, et al. Diet, exercise, and endothelial function in obese adolescents. *Pediatrics.* 2015;135(3): e653-e661

35. Bustos P, Orias J, Sáez K, Maldonado M, Cuadra L, Asenjo S. Effects of the Bright Bodies Program in Chilean obese children. *Revista Medica De Chile.* 2015;143(9):1136-1143

36. Calcaterra V, Larizza D, Codrons E, et al. Improved metabolic and cardiorespiratory fitness during a recreational training program in obese children. *Journal of Pediatric Endocrinology.* 2013; 26(3-4):271-276

37. Dobe M, Geisler A, Hoffmann D, et al. [The Obeldicks concept. An example for a successful outpatient lifestyle intervention for overweight or obese children and adolescents]. *Bundesgesundheitsblatt, Gesundheitsforschung, Gesundheitsschutz*. 2011; 54(5):628-635

38. Farpour-Lambert NJ, Aggoun Y, Marchand LM, Martin XE, Herrmann FR, Beghetti M. Physical activity reduces systemic blood pressure and improves early markers of atherosclerosis in pre-pubertal obese children. *Journal of the American College of Cardiology*. 2009; 54: 2396-406 DOI: 10.1016/j.jacc.2009.08.030

For peer review only

39. Ford AL, Hunt LP, Cooper A, Shield JP. What reduction in BMI SDS is required in obese adolescents to improve body composition and cardiometabolic health? Archives of Disease in Childhood. 2010; 95;256-261. DOI: 10.1136/adc.2009.165340

40. Ford AL, Bergh C, Sabin MA, Hollinghurst S, Hunt LP, Shield JP. Treatment of childhood obesity by retraining eating behaviour: randomised controlled trial. *British Medical Journal,* 2010; 340: p. b5388

41. Gajewska J, Kuryłowicz A, Mierzejewska E, et al. Complementary Effects of Genetic Variations in LEPR on Body Composition and Soluble Leptin Receptor Concentration after 3-Month Lifestyle Intervention in Prepubertal Obese Children. *Nutrients.* 2016;8(6). pii: E328. doi: 10.3390/nu8060328

42. Garanty-Bogacka B, Syrenicz M, Goral J, et al. Changes in inflammatory biomarkers after successful lifestyle intervention in obese children. *Endokrynologia Polska.* 2011;62(6):499-505

43. Grønbæk H, Lange A, Birkebæk NH, et al. Effect of a 10-week weight loss camp on fatty liver disease and insulin sensitivity in obese Danish children. *Journal of Pediatric Gastroenterol.Nutr*. 2012; 54(2):223-228

44. Kazankov K, Møller HJ, Lange A, et al. The macrophage activation marker sCD163 is associated with changes in NAFLD and metabolic profile during lifestyle intervention in obese children. *Pediatric Obesity*. 2015;10(3):226-233

45. Hvidt KN, Olsen MH, Ibsen H, Holm JC. Effect of changes in BMI and waist circumference on ambulatory blood pressure in obese children and adolescents. *Journal of Hypertension.* 2014; 32(7):1470-1477

46. Kirk S, Zeller M, Claytor R, Santangelo M, Khoury PR, Daniels SR. The relationship of health outcomes to improvement in BMI in children and adolescents. *Obesity Research*. 2005; 13(5): 876-882

47. Klijn P, van der Baan-Slootweg O, van Stel HF. Aerobic exercise in adolescents with obesity: preliminary evaluation of a modular training program and the modified shuttle. BMC Pediatrics. 2007; 7(19): doi:10.1186/1471-2431-7-19

48. Lazzer S, Molin M, Stramare D, Facchini S, Francescato MP. Effects of an eight-month weight-control program on body composition and lipid oxidation rate during exercise in obese children. *Journal of Endocrinological Investigation.* 2008; 31(6): 509-514

49. Meyer AA, Kundt G, Lenschow U, Schuff-Werner P, Kienast W. Improvement of early vascular changes and cardiovascular risk factors in obese children after a six-month exercise program. *Journal of the American College of Cardiology*. 2006; 48(9):1865-1870

50. Miraglia F, de Moraes Silveira CR, Gomes Beghetto M, dos Santos Oliveira F, de Mello ED. Behavior of adipokines after a year follow-up in the Obesity Outpatient Clinic for Children and Adolescents. *Nutricion Hospitalaria*. 2015; 32(4):1554-1559

For peer review only - http://bmjopen.bmj.com/site/about/guidelines.xhtml

51. Morell-Azanza L, García-Calzón S, Rendo-Urteaga T, et al. Serum oxidized low-density lipoprotein levels are related to cardiometabolic risk and decreased after a weight loss treatment in obese children and adolescents. *Pediatric Diabetes.* 2017; 18: 392-398

52. Rendo-Urteaga T, García-Calzón S, González-Muniesa P, et al Peripheral blood mononuclear cell gene expression profile in obese boys who followed a moderate energy-restricted diet: Differences between high and low responders at baseline and after the intervention. *British Journal of Nutrition*. 2015; 113(2): 331-342

53. Murer SB, Knöpfli BH, Aeberli I, et al. Baseline leptin and leptin reduction predict improvements in metabolic variables and long-term fat loss in obese children and adolescents: A prospective study of an inpatient weight-loss program. *American Journal of Clinical Nutrition*. 2011; 93(4): 695-702

54. Aeberli I, Jung A, Murer SB, et al. During rapid weight loss in obese children, reductions in TSH predict improvements in insulin sensitivity independent of changes in body weight or fat. *The Journal of clinical endocrinology and metabolism*. 2010; 95(12):5412-8

55. Murdolo G, Tortoioli C, Celi F, et al. Fetuin-A, adiposity-linked insulin resistance and responsiveness to an educational-based weight excess reduction program: a population-based survey in prepubertal schoolchildren. *Endocrine*. 2017; 56(2): 357-365

56. Ning Y, Yang S, Evans RK, et al. Changes in body anthropometry and composition in obese adolescents in a lifestyle intervention program. *European Journal of Nutrition.* 2014; 53(4):1093-1102

57. Bean M, Mazzeo SE, Stern M, et al. Six-month dietary changes in ethnically diverse, obese adolescents participating in a multidisciplinary weight management program. *Clin Pediatr*. 2011;50(5):408-416

58. Pacifico L, Arca M, Anania C, Cantisani V, Di Martino M, Chiesa C. Arterial function and structure after a 1-year lifestyle intervention in children with nonalcoholic fatty liver disease. *Nutrition, metabolism, and cardiovascular diseases.* 2013; 23(10):1010-6

59. Racil G, Ben Ounis O, Hammouda O, et al. Effects of high vs. moderate exercise intensity during interval training on lipids and adiponectin levels in obese young females. *European Journal of Applied Physiology*. 2013; 113(10):2531-40

60. Racil G, Coquart JB, Elmontassar W, et al. Greater effects of high- compared with moderate-intensity interval training on cardio-metabolic variables, blood leptin concentration and ratings of perceived exertion in obese adolescent females. *Biology of Sport*. 2016; 33(2): 145-152

61. Reinehr T, Roth C, Menke T, Andler W. Adiponectin before and after weight loss in obese children. *The Journal of Clinical Endocrinology and Metabolism*. 2004; 89(8): 3790-4

62. Reinehr T, Stoffel-Wagner B, Roth CL. Retinol-binding protein 4 and its relation to insulin resistance in obese children before and after weight loss. *The Journal of Clinical Endocrinology and Metabolism.* 2008; 93(6):2287-93

For peer review only

63. Reinehr T, Roth CL. Fetuin-A and its relation to metabolic syndrome and fatty liver disease in obese children before and after weight loss. *The Journal of Clinical Endocrinology and Metabolism*. 2008; 93(11):4479-85

64. Rohrer TR, Rizzo F, Cäsar JJ, Muelbredt O, et al. Changes in Hepatic Risk Factors, Metabolic Variables, Body Composition, and Physical Fitness in Obese Children After a One-Year Weight Loss Program. *Journal of Pediatric Endocrinology & Metabolism,* 2008; 21(9): 837-845

65. Rolland-Cachera MF, Thibault H, Souberbielle JC, et al. Massive obesity in adolescents: dietary interventions and behaviours associated with weight regain at 2 y follow-up. *International journal of obesity and related metabolic disorders*. 2004; 28(4):514-519

66. Roth, CL, Elfers C, Lass N, Reinehr T. Betatrophin: No relation to glucose metabolism or weight status in obese children before and after lifestyle intervention. *Pediatric Diabetes.* 2017; 18: 485-491

67. Savoye M, Berry D, Dziura J, et al. Anthropometric and psychosocial changes in obese adolescents enrolled in a Weight Management Program. *Journal of the American Dietetic Association*. 2005; 105(3):364-370

68. Savoye M, Nowicka P, Shaw M, et al. Long-term Results of an Obesity Program in an Ethnically Diverse Pediatric Population. *Pediatrics.* 2011; 127(3):402-410

69. Savoye M, Shaw M, Dziura J, et al. Effects of a Weight Management Program on Body Composition and Metabolic Parameters in Overweight Children. *JAMA*. 2007; 297(24):2697-2704

70. Savoye M, Caprio S, Dziura J, et al. Reversal of early abnormalities in glucose metabolism in obese youth: Results of an intensive lifestyle randomized controlled trial. *Diabetes Care*. 2014;37(2):317 – 324.

71. Schiel R, Kaps A, Günter Stein G, Steveling A. Identification of Predictors for Weight Reduction in Children and Adolescents with Overweight and Obesity (IDA-Insel Survey). *Healthcare*. 2016; 4(1): 07. doi:10.3390/healthcare4010005

72. Seabra A, Katzmarzyk P, Carvalho MJ, et al. Effects of 6-month soccer and traditional physical activity programmes on body composition, cardiometabolic risk factors, inflammatory, oxidative stress markers and cardiorespiratory fitness in obese boys. *Journal of Sports Sciences*. 2016; 34(19):1822-1829

73. Truby H, Baxter K, Ware RS, et al. A Randomized Controlled Trial of Two Different Macronutrient Profiles on Weight,Body Composition and Metabolic Parameters in Obese Adolescents Seeking Weight Loss. *PLoS ONE [Electronic Resource]*. 2016; 11(3): e0151787

74. van der Baan–Slootweg O, Benninga MA, Beelen A, van der Palen J, et al. Inpatient treatment of children and adolescents with severe obesity in the Netherlands. A Randomized Clinical Trial. *JAMA Pediatr,* 2014; 168(9):807-814.

For peer review only

For peer review only - http://bmjopen.bmj.com/site/about/guidelines.xhtml

75. Visuthranukul C, Sirimongkol P, Prachansuwan A, Pruksananonda C, Chomtho S. Low-glycemic index diet may improve insulin sensitivity in obese children. *Pediatric Research*. 2015;78(5):567-573

76. Vitola BE, Deivanayagam S, Stein RI, et al. Weight loss reduces liver fat and improves hepatic and skeletal muscle insulin sensitivity in obese adolescents. *Obesity*. 2009; 17:1744-1748

77. Wickham EP, Stern M, Evans RK, et al. Prevalence of the Metabolic Syndrome Among Obese Adolescents Enrolled in a Multidisciplinary Weight Management Program: Clinical Correlates and Response to Treatment. *Metabolic Syndrome and Related Disorders*. 2009; 7(3):179-186

78. Evans RK, Franco RL, Stern M, et al. Evaluation of a 6-month multi-disciplinary healthy weight management program targeting urban, overweight adolescents: Effects on physical fitness, physical activity, and blood lipid profiles. *International Journal of Pediatric Obesity*. 2009; 4(3):130-133

79. Tanner JM. Growth at adolescence: with a general consideration of the effects of hereditary and environmental factors upon growth and maturation from birth to maturity. 2nd ed. Oxford, England: Blackwell Scientific, 1962

80. Wan X, Wang W, Liu J, Tong T. Estimating the sample size and standard deviation from the sample size, median, range and/or interquartile range. *BMC Medical Research Methodology*. 2014; 14:135 doi: 10.1186/1471-2288-14-135

81. Flegal, KM, et al. Characterizing extreme values of body mass index-for-age by using the 2000 Centers for Disease Control and Prevention growth charts. *Am J Clin Nutr*. 2009; 90(5): 1314-1320

82. Freedman DS, Butte NF, Tavares EM, Lundeen EA, Blanck, HM, Goodman AB, Ogden CL. BMI z-scores are a poor indicator of adiposity among 2- to 19-year -olds with very high BMIs, NHANES 1999-2000 to 2013-2014. *Obesity*, 2017; 25(4):739–746. doi:10.1002/oby.21782

83. Cole, TJ, et al. "What is the best measure of adiposity change in growing children: BMI, BMI %, BMI z-score or BMI centile?" *Eur J Clin Nutr*. 2005; 59(3): 419-425

84. Juliusson, PB, et al. Severe obesity is a limitation for the use of body mass index standard deviation scores in children and adolescents. *Acta Paediatr*. 2018; 107(2): 307-314

85. Vanderwall, C, et al. BMI is a poor predictor of adiposity in young overweight and obese children. *BMC Pediatr*. 2017*;* 17(1): 135

86. Woo JG, Cole TJ. Assessing adiposity using BMI-Z scores in children with severe obesity. Obesity, 2017; 25(4); 662

87. Reinehr T. Lifestyle intervention in childhood obesity: changes and challenges. *Nat Rev Endocrinol*. 2013; 9(10):607-614

For peer review only - http://bmjopen.bmj.com/site/about/guidelines.xhtml

88. Al-Khudairy L, Loveman E, Colquitt JL, et al. Diet, physical activity and behavioural interventions for the treatment of overweight or obese adolescents aged 12 to 17 years. *Cochrane Database Syst Rev*. 2017 Jun 22;6:CD012691. doi: 10.1002/14651858.CD012691

89. Mead E, Brown T, Rees K, et al. Diet, physical activity and behavioural interventions for the treatment of overweight or obese children from the age of 6 to 11 years. *Cochrane Database Syst Rev*. 2017 Jun 22;6:CD012651. doi: 10.1002/14651858.CD012651

For peer review only

For peer review only - http://bmjopen.bmj.com/site/about/guidelines.xhtml

Figure 1: Flow diagram from the systematic review that identified the included studies

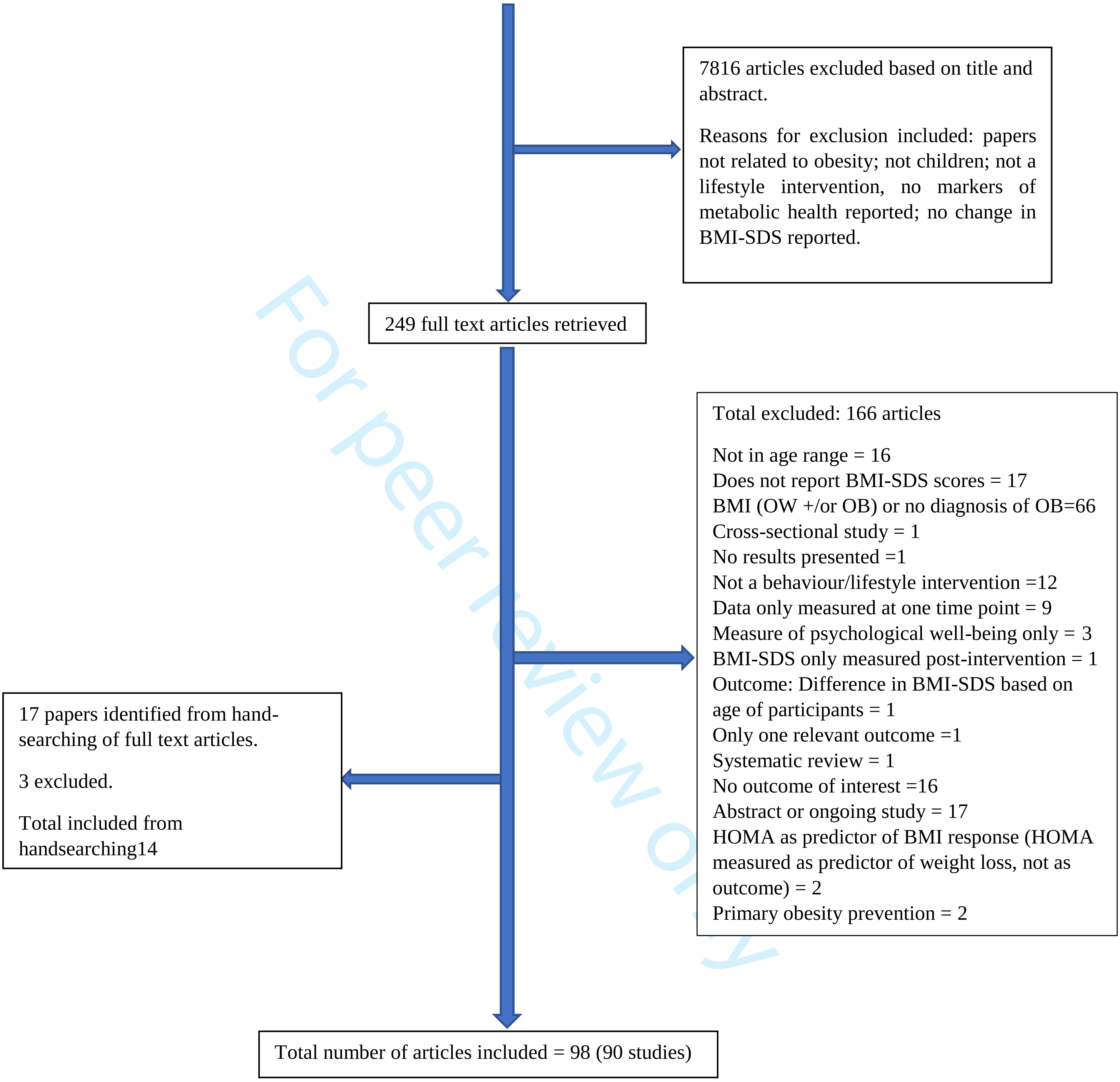

For peer review only

Figure 2: Venn diagram illustrating the markers of metabolic health measured

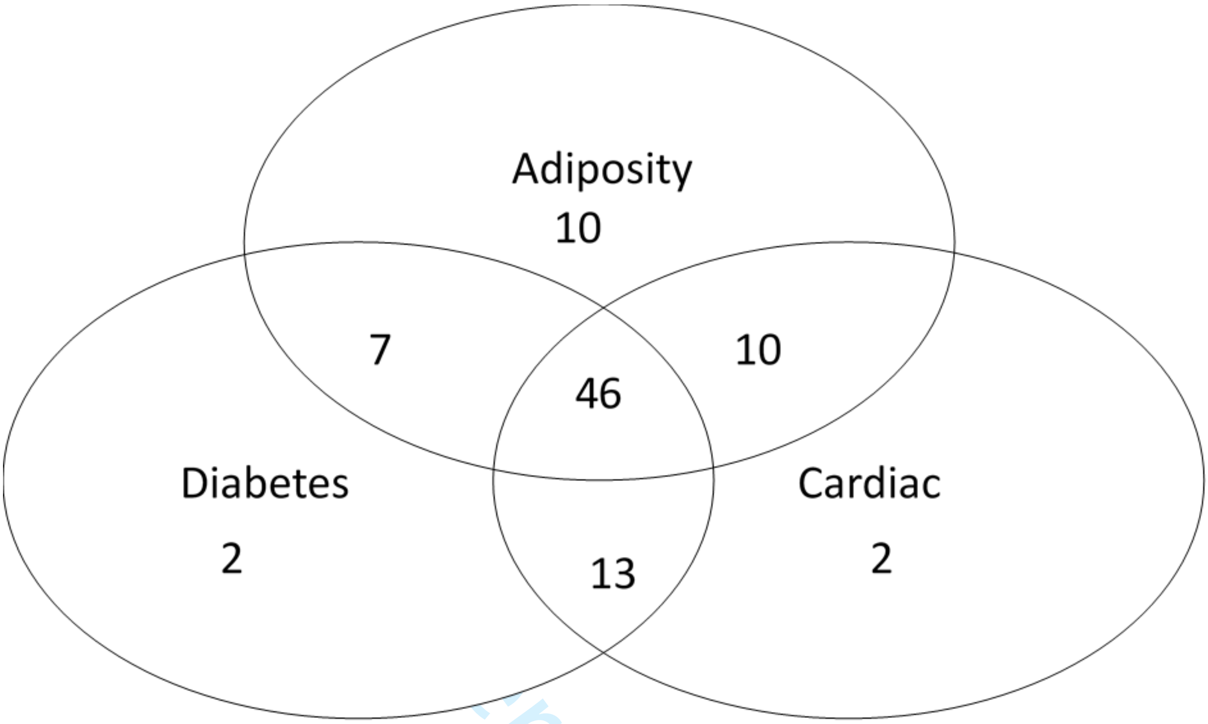

TOTAL NUMBER OF STUDIES = 90

For peer review only

Figure 3: Meta-regression line showing the relationship between mean change in percentage body fat and BMI-SDS across the 39 studies (66 subsets) analysed

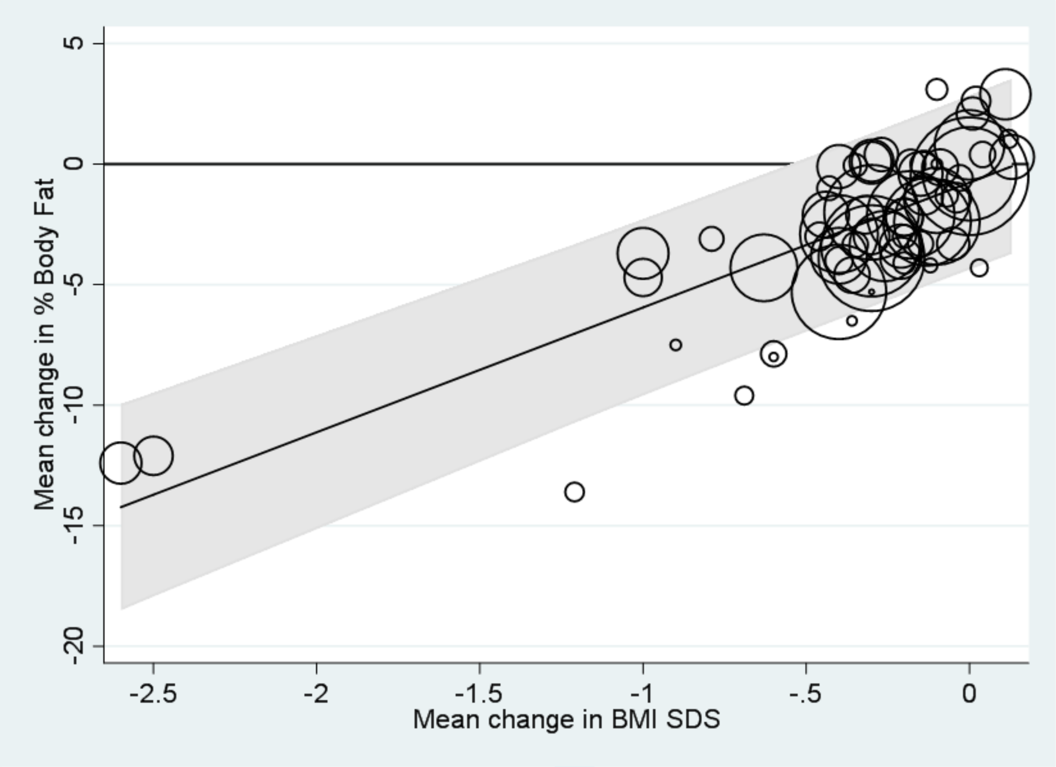

For peer review only

Figure 4: Normal plot for the standardised predicted random effects from the meta-regression

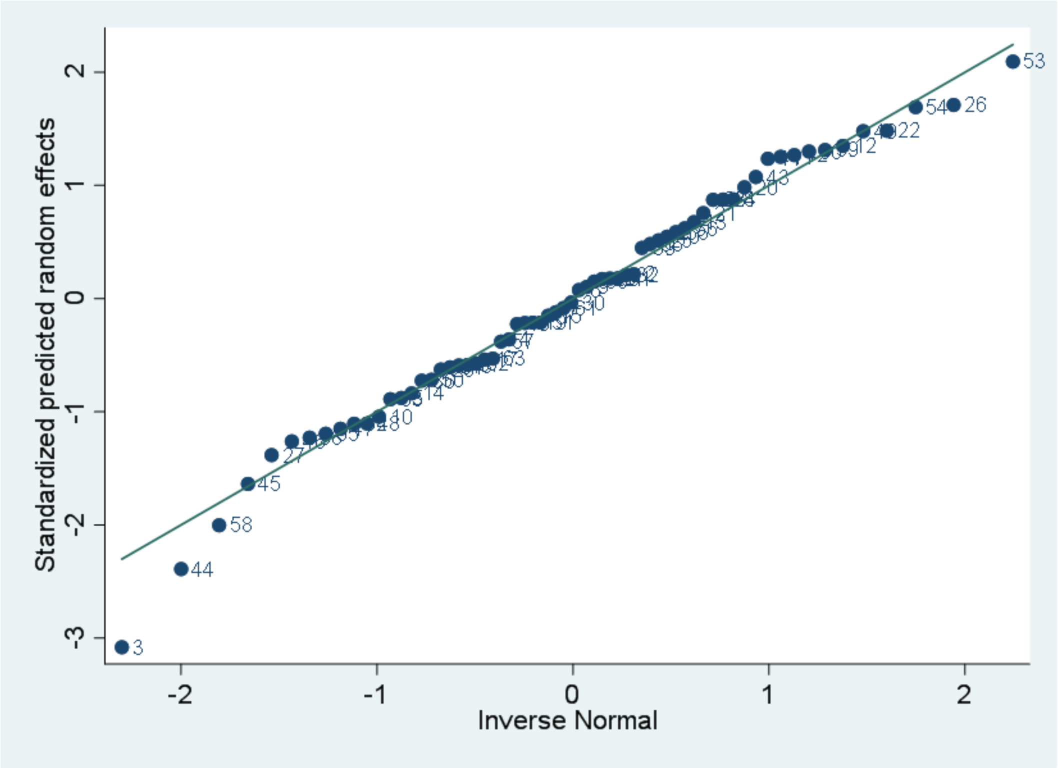

Figure 5: Sensitivity Analysis

Sensitivity Analysis i: Based on the 22 subsets where the SEs of the mean changes in percentage body fat were known (Fitted meta-regression line: Mean change in % body fat = 4.502 x Mean change in BMI SDS – 0.810).

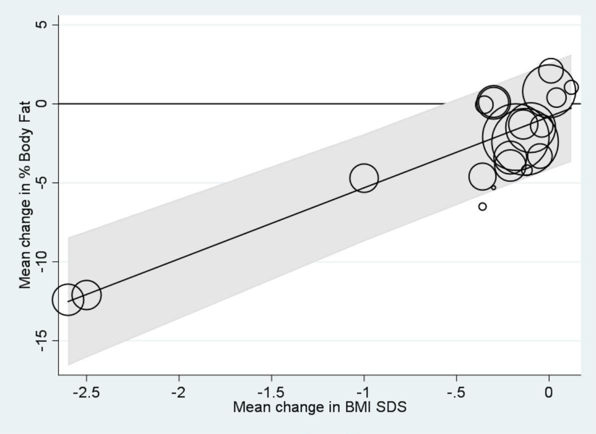

Sensitivity Analysis ii: Using all data subsets but excluding two extreme values (reduction of mean BMI-SDS of more than 1.5), leaving 64 subsets (Fitted meta-regression line: Mean change in % body fat = 7.078 x Mean change in BMI SDS – 0.318).

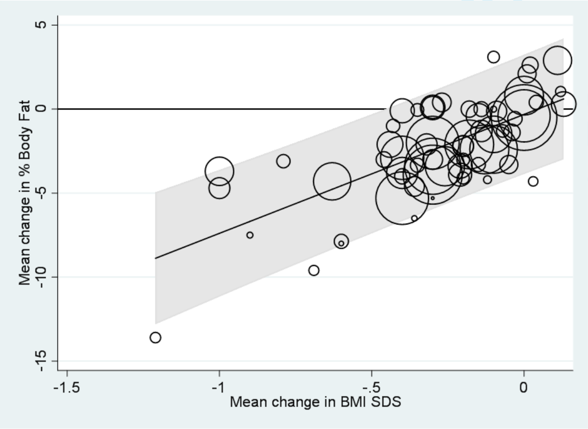

Sensitivity Analysis iii: Using all 66 data subsets but using a correlation coefficient of 0.50, rather than 0.81, to estimate the SE of the mean change in % body fat for the 66-22=44 subsets where this was not available (Fitted regression line: Mean change in % body fat = 5.039 x Mean change in BMI-SDS - 0.783).

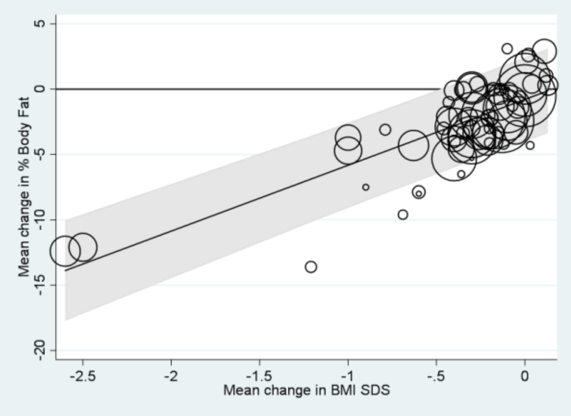

For peer review only

APPENDIX 1: Childhood obesity BMI systematic review_MEDLINE

1. exp Child/
2. exp Adolescent/
3. juvenile.tw.
4. exp Infant/
5. exp Pediatrics/
6. child$.tw.
7. infant$.tw.
8. teen$.tw.
9. p?ediatric$.tw.
10. young person.tw.
11. schoolchild$.tw.
12. youth.tw.
13. (boy$ or girl$).tw.
14. 1 or 2 or 3 or 4 or 5 or 6 or 7 or 8 or 9 or 10 or 11 or 12 or 13
15. exp body weight/
16. exp energy metabolism/
17. exp obesity/
18. exp childhood obesity/
19. exp metabolic syndrome X/
20. exp metabolic disorder/
21. (metabol$ adj1 disorder$).ti,ab.
22. (metabol$ adj1 syndrome$).ti,ab.
23. (cardiometabolic or cardio-metabolic or cardio metabolic).ti,ab.
24. (weight adj3 (cyc$ or reduc$ or los$ or maint$ or decreas$ or watch$ or control$ or gain$ or chang$)).tw.
25. (body fat or body fat percent$ or percent$ body fat or fat mass or adipos$).ti,ab.
26. waist-hip ratio$.tw.
27. waist circumferenc$.ti,ab.
28. (lean adj1 body adj1 mass).ti,ab.
29. (percentage adj1 body adj1 fat).ti,ab.
30. fat.ti,ab.
31. obes$.ti,ab.
32. (overweight or over weight or over-weight).ti,ab.
33. exp abdominal fat/
34. adipose tissue/
35. ((food or energy or calor$) adj1 intake).ti,ab.
36. (BMI or body mass ind$ or body-mass-ind$ or weight for height or weight-for-height).ti,ab.
37. (overfeed$ or over feed$).tw.
38. (overeat$ or over eat$).tw.
39. exp weight gain/
40. exp weight reduction/
41. (weight adj1 los$).ti,ab.
42. (fat adj1 los$).ti,ab.
43. 15 or 16 or 17 or 18 or 19 or 20 or 21 or 22 or 23 or 24 or 25 or 26 or 27 or 28 or 29 or 30 or 31 or 32 or 33 or 34 or 35 or 36 or 37 or 38 or 39 or 40 or 41 or 42
44. (BMI adj5 z score).af.
45. (BMI adj5 SDS).af.
46. (BMI adj5 standard adj1 deviation).af.
47. (Body adj1 mass adj1 index adj5 sd adj1 score).af.
48. (Body adj1 mass adj1 index adj5 SD).af.
49. 44 or 45 or 46 or 47 or 48
50. 14 and 43 and 49

For peer review only

Appendix 2: Characteristic of studies excluded from meta-regression

	Author, Year, Country (Intervention name)	Study design: Sample size (n) Analysed (An)	Obesity definition	Diet D)/ Exercise (E)/D+E: Setting	Format & content	Adiposity outcome measurement or reasons for exclusion for meta-regression
1.	Carraway 2014[1] USA	Cohort<br>Total = 52 (subgroup n =33 & families offered FU for 10 months)	BMI > 95th %ile	Community	6 x 1 hr nutrition sessions + ad-lib access to a nutritious diet + 7hr/day PA + GT + CBT.	WC only
2.	Croker 2012[2] UK	RCT:<br>Total = 72 (63)<br>FBBT= 37 (33)<br>OC =35 (30)	OW or Ob as per IOTF<br>Mean BMI SDS>3	D+E<br>Clinic (Hospital)	Reduce snacking ≤ 2 occasions/day + Balanced diet following 'Eatwell plate and 'Traffic Light system' + Reduce sedentary behaviour + 60 min/day exercise.	body mass, fat mass, WC &WC-SDS
3.	Doughty 2015[3] USA	Cohort<br>Total = 12	BMI ≥ 89th %ile. Requested data from study authors regarding OB/OW– no response.	D+E<br>Academic Institution	Behavioural counselling + Daily caloric targets + 2 x 1hr/5 days and one 1hr/day physical training) + behavioural counselling.	WC. body mass
4.	Elloumi 2009[4] Tunisia	RCT<br>Total =21<br>Energy restriction =7<br>Exercise training =7<br>Both =7	BMI > 97th %ile (French standards)	D+E<br>Academic Institution	2-month intervention. 3 groups, R = energy restricted group. E=individualised exercise group. RE= energy restriction + exercise. Individualized dietary advice by dietitian. 500kcal/day deficit (15% protein, 55% CHO, 30% fat). Exercise: 90 mins per day; 4 days per week. Intensity of exercise at heart rate corresponding to $lipox_{max}$	Body mass, fat mass
5.	Grulich-Henn, 2011[5] Germany	Cohort Total = 58	BMI > 97th %ile (German paed. standards)	D + E<br>Academic Institution	6 x monthly nutritional consultation & CBT + 24 weekly PA programs.	Body mass only
6.	Gunnarsdottir, 2014[6] Iceland	Cohort Total = 84	BMI z-score > 2.0 SDS (Swedish growth curve)	D+E<br>Clinic (Hospital)	Family-based Epstein behavioural intervention.	Body mass only
7.	Holm 2007[7] Denmark	Cohort<br>Total enrolled =120; BL =110, post-intervention = 87	BMI-SDS LMS method (Danish ref pop.)	D+E<br>Academic Institution	Restricted low-fat diet (6500-7000 kJ/day) + Mandatory and optional PA.	number of pps not consistent for BMI-SDS and % body fat
8.	Kalavainen, 2012[8] Finland	RCT<br>Total = 70<br>routine treatment = 35,<br>group treatment = 35	Wt-for-ht 115-182%	D+E<br>Community	2 interventions (Group and routine) - Routine (2 school heath care sessions) + Group (10 x 90 min/wk parents and children separate focusing on healthy lifestyle/physical activity session, then next 5 sessions/2 wks + 1 session together)	Fat mass only
9.	Kolsgaard, 2011[9] Norway	Cohort<br>Total = 230 analysed (n= 307 started)	BMI > 97.5 %ile for ht according to Norwegian percentiles.	D+E<br>Clinic (Hospital)	~1hr biannual diet & PA (60 min/day exercise encouraged) counselling session with children & parent/s.	Body mass, WC
10.	Kolotourou 2013[10] UK **MEND**	Cohort: Recruited from MEND RCT.<br>Total = 230 analysed<br>Subsample 1 = 71 (6 mth RCT completers – both arms)	BMI ≥ 98th %ile	D+E:<br>Community	Family-based 9-week MEND program (2 x wk group sessions including nutrition education, behaviour modification + fun-based PA) + 12 wk free family swim pass.	WC only

For peer review only

		Subsample 2 = 42 (12 mth RCT completers – IG only)				
11.	Marcano, 2011[11] Venezuela	Cohort<br>Total = 111	OW: BMI >90th %ile/BMI z-score > 1.5.<br>OB: BMI>97th %ile/BMI z-score >2	D+E:<br>Clinic (Hospital)	Nutrition+PA recommendations + A form to register wkly hours of PA, number of steps taken/day, and hrs/wk spent in sedentary activities + Restrict calorie intake and focus on a balanced diet encouraged.	Body fat only
12.	Mager 2015[12]	Cohort<br>Total=12 (completed =9)	CDC criteria	D Clinic (but unclear)	1 session of education for parents and children and then follow up at 3 months and 6 months afterwards.	WC only
13.	Makkes 2016[13] Netherlands	RCT:<br>Total = 80<br>Short-stay (SS)=40<br>Long-stay (LS)=40	BMI-SDS ≥3.0 or BMI-SDS ≥2.3 + OB-related comorbidity	D+E:<br>Clinic (Hospital)	Intensive 12-month lifestyle treatment. In-patient period of either 2 months (short-stay group) or 6 months (long-stay group). Short-stay group: biweekly 2-day return visits for 4 months, then monthly 2-day return visits for 6 months following in-pt period. Long-stay group: monthly 2-day return visits for 6 months following in-pt period.<br>Treatment: Nutrition, physical activity and behaviour change. Required active participation of parents/caregivers.	WC,WC-SDS
14.	Martos, 2009[14] Spain (Same intervention as Valle Jimenez 2013[23] but different sample)	Cohort<br>Total = 47	BMI > 95th %ile on growth curves	D+E<br>Community	Moderately OB subjects (Low-calorie diet); Severe/refractory OB subjects (Restriction diet of 25-30%) + Moderate/intense exercise 60 min/day x 5 days/wk encouraged.	Body mass only
15.	Obert 2013[15] France	Cohort<br>Total = 28 (plus 20 healthy lean controls)	BMI > 97th French %ile	D+E:<br>Clinic (Hospital)	Cycle ergometer (9 x 5 mins x 3 times/week: 4 min moderate + 1 min intense) + 2 times/wk moderate exercise for 1st 2 mths	Body mass, fat mass
16.	Panagiotopoulos2011[16] Canada	Cohort<br>Total =119	OB: BMI ≥ 95th %ile; OW: BMI ≥ 85th %ile and <95th %ile with at least 1 comorbidity	D+E:<br>Clinic (Hospital)	10 x consecutive wkly group sessions (6-10 families): 30 min PA + nutrition session + behavioural session.	Body mass only
17.	Pozzato[17] Verduci 2011[18] Italy	Cohort:<br>Total = 26	>30kg/m2 age and sex adjusted Cole *et al* curve	D+E.<br>Community and Clinic	Normocaloric balanced diet and active lifestyle based on italian guidelines for treatment of childhood obesity	WC only
18.	Reinehr, 2004b[19] Germany OBELDICKS	Cohort:<br>Total = 57	BMI ≥ 97th %ile	D+E:<br>Clinic (Hospital)	Obeldicks - Intensive phase 3 mnths (Parents' course 2x/mnth + Behaviour therapy 2x/mnth + Nutritional course 2x/mnth + Exercise therapy 1x/wk) + Establishing phase 3 mnths (Talk rounds for parents 1x/mnth + Psychological therapy + Exercise therapy 1x/wk) + Establishing phase 2 for 3 mnths (Psychological therapy + Exercise therapy 1x/wk) + Establishing phase 3 for 3 mnths (Exercise therapy 1x/wk).	Body fat only
19.	Reinehr, 2009[20] Germany OBELDICKS	Cohort<br>Total = 109 (plus 43 obese controls)	IOTF criteria: OB	D+E:<br>Clinic (Hospital)	Obeldicks (as above)	WC only
20.	Rijks 2015[21] Netherlands	Non-randomised prospective study<br>Total = 172	IOTF criteria: OW, OB, MO	D+E:<br>Clinic (Hospital)	Guidance with focus on nutrition, food habits, PA, sleep, psychological and social aspects.	WC-SDS only
21.	Rovira 2013[22] Spain	Cohort<br>Total = 110	BMI ≥ 97th %ile	D+E<br>Clinic (Hospital)	12 x monthly visits in 2 phases: motivational and intervention. Focus on promoting healthy eating, encouraging PA & decreasing sedentary behaviour.	Only reported as 'good responders' and 'poor responders' to

						intervention so removed from analysis
22.	Santomauro 2011[23] Venezuela	Cohort Total = 36	BMI > 97th %ile	D+E: Clinic (Hospital)	Dietary recommendations + 30 mins daily moderate exercise or 3 x wk moderate exercise + decrease time watching TV/video games.	Body mass, fat mass
23.	Schum 2012[24] Germany	Cohort: Total = 75 HZ=52 HO= 21	BMI-SDS > 2	E: Community	Increase to 2 hrs/day PA + nutritional recommendations based on 'Optimised Mixed Diet for German Children and Adolescents' + close surveillance by physician.	Fat mass, WC
24.	Shalitin, 2009[25]	Cohort: Total = 174 randomised E =58 (52) D =58 (55) D+E = 58 (55)	BMI > 95th %ile for age & gender	D+E: Clinic (Hospital)	3-month interventions: Exercise intervention (90 min moderate exercise 3 days/wk); Diet intervention 3 mths (12 x/wk nutritional group meetings with parents + Hypocaloric diet 1200 kcal/day); Diet and exercise intervention 3 mths (90 min training session days/wk + 12 x/wk nutritional group meetings with parents + Hypocaloric diet 1200 kcal/day).	SEs were calculated from a mixed model and not directly comparable
25.	Springer 2015[26] Germany	Cohort: Total=39	BMI > 90th %ile	D+E: Clinic (Hospital)	Encouraged to increase exercise by 1-2 hrs/day + Decrease sedentary behaviour to a total of 2 hrs/day or less + Nutrition recommendations + 6 telephone calls from/visits to the physician.	fat mass, WC & WC-SDS
26.	Tan-Ting 2011[27] Philippines	Cohort: Total = 44	BMI ≥ 95th %ile (CDC)	D+E: Clinic (Hospital)	Multidisciplinary, individualised, behavioural modification and exercise programme (St Luke's Medical Center Obesity & Weight Management Program) Dietary session (6 sessions over 3 mths) + Restricted diet (1200-1500 Kcal/day) + Physical activity (24 x 1hr sessions over 3 mths + encouraged to do ≥ 30 min of individual exercise) + Behavioural management (4 x sessions over 3 mths).	body mass, fat mass, WC &WC-SDS
27.	Valle Jiminez 2013[28] Spain	Cohort Total = 50 (plus n=50 non-obese control)	BMI >95th percentile growth curves for Spanish pop.	D+E: Academic Institution/Clinic (Hospital)	Behavioural components, physical exercise and nutritional education. Energy distribution of diet: 25% between breakfast & lunch; 30-35% at lunch; 15% afternoon snack; remainder dinner. Moderate-to-intense PA for 30 mins at least 3 days per wk. Aim that 1 month after the start of tx subjects should be engaging in 60 mins/day moderate-to-intense physical exercise.	Body mass only
28.	Vanhelst 2013[29] France	Cohort Total=37	ND	D+E: Community	2hr/wk exercise sessions + 2hr/3 months heath education session.	Fat mass, fat free mass
29.	Vasquez 2013[30] Chile	Cohort X-over trial (Group 1 only) Total = 60	BMI ≥ 95th %ile CDC	D+E: Academic Institution	Group nutrition education sessions x 6 (5 for children; 1 for parents) Psychologist support sessions x 6 (5 for children; 1 for parents) PE 45 mins x3/wk (30 sessions in total)	Body mass, WC
30.	Verduci 2015[31] Italy	Cohort: Total = 85	BMI Cole's curve cut-off 30 kg/m^2 at 18 yrs	D+E: Clinic (Hospital)	Normocaloric balanced diet + 60 min/day moderate/vigorous exercise + 1 hr educational session with dietician at recruitment.	WC only
31.	Vos, 2011[32] Netherlands	RCT Total = 81 (BL: 79 An. 69) I = 41 (BL 40: An. 36) OC=40 (BL 39: An. 33)	Cole *et al* criteria	D+E: Clinic (Hospital)	12 mths: During first 3 mths (7 x 2.5 hr/2 wks children group meetings + 5 x 2.5 hr/2 wks parent meetings + 1 x 2.5 hr/2 wks child/parent meeting + 2-3 refresher follow-up sessions for total of 2 yrs). Also included exercise however not described except in flow diagram	WC-SDS only
32.	Weiss 2009[33] USA Yale **TEAMS**	Cohort: Total = 186	BMI > 95th %ile (CDC)	D+E: Clinic (Hospital)	Subjects followed biannually as outpatients + Received nutritional/PA guidance. Levels of adherence to these recommendations was not evaluated or documented	Body mass only

For peer review only

33.	Weigel 2008[34]<br>Germany	RCT:<br>Total = 73<br>IG = 37<br>OC = 36	OW BMI > 90th %ile<br>OB BMI > 97th %ile<br>Extremely OB BMI > 99.5th %ile	D+E:<br>Community	Twice wkly 45-60 min sessions on exercise/dietary education/coping strategies.	Fat mass only
34.	Wong 2009 [35]<br>USA	Cohort:<br>Total = 21	BMI ≥ 95th %ile	D+E:<br>Community	6 x 1hr behavioural lessons + 4 x 1hr PA/ nutrition lessons + 1800 kcal/day diet.	body mass only

KEY: %ile = percentile; AmO = Outpatient Ambulatory; An. = analysed; apt. = appointment; BB =Bright Bodies; BFC = Better food choices; BL = baseline; BM = behaviour modification; BMI= body mass index; C = control; CG: control group; CBT = cognitive behavioural therapy; CDC = Centre for Disease Control; CG = control group; CHO = carbohydrate; D = diet; E = exercise; FBBT = family-based behavioural treatment; F = female; FU = follow up; GI = glycaemic index; GT = group therapy; HGI = high glycaemic index; hr = hour; HZ = heterozygous; HO = homozygous; ht = height; I = intervention; IG= intervention group; IOTF = International Obesity Task Force; Inpt. = inpatient; LGI = low glycaemic index; LMS= least-mean-squares; LS = long stay; min= minute; mth = month; MO = morbidly obese; norm. normal; n = number; NAFLD = Non-alcoholic fatty liver disease; ND = not described; NR = not reported; OB = obese; OC = obese control; OW = overweight; paed. = paediatric; PA = physical activity; PE = physical activity; PROT= protein; ppts= participants; RCT = randomised controlled trial; SD = standard deviation; SDS = standard deviation score; SE: standard error; SMP= Structured meal plan; SS= short stay; SMC= structured modified carbohydrate diet; trad. = traditional; Trad. act = traditional activity; tx = treatment; TEAMS = Tracking Endpoints in Adolescent MS; wk = week; WList OC– wait list obese control; WL = weight loss; wt = weight; X-over = crossover; yr = year

For peer review only

1. Carraway ME, Lutes LD, Crawford Y, et al. Camp-based immersion treatment for obese, low socioeconomic status, multi-ethnic adolescents. *Childhood obesity* (Print). 2014;10(2): 122-131

2. Croker H, Viner RM, Nicholls D. Family-based behavioural treatment of childhood obesity in a UK National Health Service setting: randomized controlled trial. *International Journal of Obesity*. 2012; 36(1):16-26

3. Doughty KN, Njike VY, Katz DL. Effects of a Cognitive-Behavioral Therapy-Based Immersion Obesity Treatment Program for Adolescents on Weight, Fitness, and Cardiovascular Risk Factors: A Pilot Study. *Childhood Obesity*. 2015;11(2):215-218

4. Elloumi M, Ben Ounis O, Makni E, Van Praagh E, Tabka Z, Lac G. Effect of individualized weight-loss programmes on adiponectin, leptin and resistin levels in obese adolescent boys. *Acta Paediatr*. 2009; 98(9):1487-93

5. Grulich-Henn J, Lichtenstein S, Hörster F, Hoffmann GF, Nawroth PP, Hamann A. Moderate weight reduction in an outpatient obesity intervention program significantly reduces insulin resistance and risk factors for cardiovascular disease in severely obese adolescents. *Int J Endocrinol*. 2011:541021

6. Gunnarsdottir T, Einarsson SM, Njardvik U, et al. Family-based behavioral treatment for obese children - results and two year follow up. *Laknabladid*. 2014;100(3):139-45

7. Holm JC, Buckert M, Hans-Christoph Friederich H-C, et al. Time course and determinants of leptin decline during weight loss in obese boys and girls. *International Journal of Pediatric Obesity*. 2007; 2(1): 2-10

8. Kalavainen M, Utriainen P, Vanninen E, Korppi M, Nuutinen O. Impact of childhood obesity treatment on body composition and metabolic profile. *World Journal of Pediatrics*. 2012; 8(1):31-7

9. Kolsgaard ML, Joner G, Brunborg C, et al. Reduction in BMI z-score and improvement in cardiometabolic risk factors in obese children and adolescents. The Oslo Adiposity Intervention Study - a hospital/public health nurse combined treatment.[Erratum appears in BMC Pediatr. 2012;12:77; PMID: 22709339]. *BMC Pediatrics*. 2011; 11: 47

10. Kolotourou M, Radley D, Gammon C, Smith L, Chadwick P, Sacher PM. Long-Term Outcomes following the MEND 7-13 Child Weight Management Program. *Childhood Obesity*. 2015;11(3): 325-330

For peer review only

11. Marcano, H, Fernández M, Paoli M, et al. Limited Weight Loss or Simply No Weight Gain following Lifestyle-Only Intervention Tends to Redistribute Body Fat, to Decrease Lipid Concentrations, and to Improve Parameters of Insulin Sensitivity in Obese Children. *International Journal of Pediatric Endocrinology*. 2011(1): 241703

12. Mager DR, Iñiguez IR, Gilmour S, Yap J. The effect of a low fructose and low glycemic index/load (FRAGILE) dietary intervention on indices of liver function, cardiometabolic risk factors, and body composition in children and adolescents with non-alcoholic fatty liver disease (NAFLD). *J Parenter Enteral Nutr*. 2015;39(1):73-84

13. Makkes S, Renders CM, Bosmans JE, van der Baan-Slootweg OH, Hoekstra T, Seidell JC. One-year effects of two intensive inpatient treatments for severely obese children and adolescents. *BMC Pediatrics*. 2016; 16:120 doi: 10.1186/s12887-016-0659-x

14. Martos R, Valle M, Morales RM, Cañete R, Gascón F, Urbano MM. Changes in body mass index are associated with changes in inflammatory and endothelial dysfunction biomarkers in obese prepubertal children after 9 months of body mass index SD score loss. *Metabolism-Clinical and Experimental*. 2009; 58(8):1153-1160

15. Obert P, Gueugnon C, Nottin S, et al. Impact of diet and exercise training-induced weight loss on myocardial mechanics in severely obese adolescents. *Obesity*, 2013; 21(10): 2091-8

16. Panagiotopoulos C, Ronsley R, Al-Dubayee M, et al. The Centre for Healthy Weights-Shapedown BC: A Family-Centered, Multidisciplinary Program that Reduces Weight Gain in Obese Children over the Short-Term. *International Journal of Environmental Research and Public Health*. 2011; 8(12):4662-4678

17. Pozzato C, Verduci E, Scaglioni S, Liver Fat Change in Obese Children After a 1-year Nutrition-behavior Intervention. Journal of Pediatric Gastroenterology and Nutrition. 2010;51(3), 331–335

18. Verduci E, Radaelli G, Salvioni M, Riva E, Giovannini M. Plasma long-chain fatty acids profile and metabolic outcomes in normolipidaemic obese children after one-year nutritional intervention. *Acta paediatrica*. 2011;100(4): 585-9

19. Reinehr T. and Andler W. Changes in the atherogenic risk factor profile according to degree of weight loss. *Archives of Disease in Childhood*. 2004;89(5):419-422

20. Reinehr T, C Schmidt C, Toschke AM, Andler W. Lifestyle intervention in obese children with non-alcoholic fatty liver disease: 2-year follow-up study. *Archives of Disease in Childhood*. 2009; 94(6):437-442

For peer review only

21. Rijks JM, Plat J, Mensink RP, Dorenbos E, Buurman WA, Vreugdenhil AC. Children with morbid obesity benefit equally as children with overweight and obesity from an ongoing care program. *Journal of Endocrinol Metab*. 2015; 100(9): 3572-3580

22. Rovira AF, París Miró N, Zaragoza-Jordana M, et al. Clinical and metabolic effectiveness of a new motivational therapy for the treatment of obesity in adolescents (OBEMAT). *Anales De Pediatria.* 2013; 78(3): 157-166

23. Santomauro M, Paoli-Valeri M, Fernández M, et al. Non-alcoholic fatty liver disease and its association with clinical and biochemical variables in obese children and adolescents: Effect of a one-year intervention on lifestyle. *Endocrinología y Nutrición* (English Edition), 2012;59(6): p. 346-353

24. Schum J, Blumenstock G, Weber K, et al. Variants of the FTO Gene in Obese Children and their Impact on Boy Composition and Metabolism before and after Lifestyle Intervention. *Experimental and Clinical Endocrinology & Diabetes*. 2012;120(3):128-131

25. Shalitin S, Ashkenazi-Hoffnung L, Yackobovitch-Gavan M, et al. Effects of a Twelve-week randomized intervention of exercise and/or diet on weight loss and weight maintenance, and other metabolic parameters in obese preadolescent children. *Hormone Research*. 2009;72(5):287-301

26. Springer F, Ballweg V, Schweizer R, et al. Changes in whole-body fat distribution, intrahepatic lipids, and insulin resistance of obese adolescents during a low-level lifestyle intervention. *European Journal of Pediatrics*, 2015;174(12):1603-12

27. Tan-Ting AM, Llido L. Outcome of a hospital based multidisciplinary weight loss program in obese Filipino children. *Nutrition.* 2011; 27:50-54

28. Valle Jimenez M, Martos R, Morales RM, et al. Relationship between changes in plasma leptin concentrations and plasminogen activator inhibitor-1 in obese prepubertal children after nine months of treatment. *Annals of nutrition & metabolism.* 2013; 63(3): 216-22

29. Vanhelst J, Fardy PS, Béghin L, Bui-Xuan G, Mikulovic J. Strategies in intervention programmes for obese youth: implication of the age and the type of physical activities. *Clinical Physiology and Functional Imaging.* 2015; 35(1): 17-20

30. Vasquez F, Diaz E, Lera L, Vasquez L, Anziani A, Burrows R. Impact of a strength training exercise program on body composition and cardiovascular risk factors in a group of obese schoolchildren by pubertal stage. *American Journal of Sports Science and Medicine.* 2014; 2(1): 40-47

31. Verduci E, Lassandro C, Giacchero R, Miniello VL, Banderali G, Radaelli G. Change in metabolic profile after 1-year nutritional-behavioral intervention in obese children. *Nutrients.* 2015; 7(12): 10089-10099

32. Vos RC, Wit JM, Pijl H, Houdijk EC. Long-term effect of lifestyle intervention on adiposity, metabolic parameters, inflammation and physical fitness in obese children: a randomized controlled trial. *Nutrition & Diabetes.* 2011; 1:e9. doi: 10.1038/nutd.2011.5

For peer review only

33. Weiss R, Shaw M, Savoye M, Caprio S. Obesity dynamics and cardiovascular risk factor stability in obese adolescents. *Pediatric Diabetes*. 2009; 10(6): 360-7

34. Wiegal C, Kokocinski K, Lederer P, Dötsch J, Rascher W, Knerr I. Childhood obesity: Concept, feasibility, and interim results of a local group-based, long-term treatment program. *J Nutr Educ Behav*. 2008; 40:369-373

35. Wong WW, Abrams SH, Mikhail C, et al. An innovative summer camp program improves weight and self-esteem in obese children. *Journal of Pediatric Gastroenterology and Nutrition*. 2009; 49(4):493-497

For peer review only

For peer review only

Appendix 3: Meta-regression line and 95% prediction interval for the relationship between the mean change in percentage body fat and BMI-SDS across the 39 studies (66 subsets), as in Figure 3, but highlighting four subgroups of younger participants:

- ● Dobe 2011[37], aged 4-8y;
- ● Gajewska 2016[41], two groups aged 5-10y;
- ● Kirk 2005[46], group 1, aged 5-10

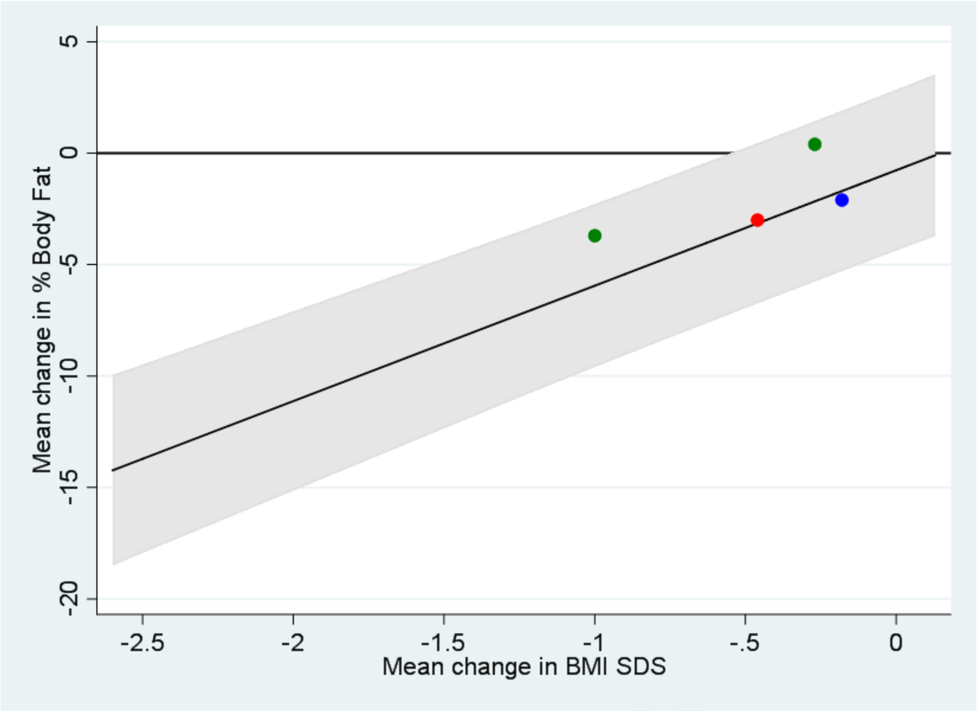

ew only

PRISMA 2009 Checklist

Section/topic	#	Checklist item	Reported on page #
**TITLE**			
Title	1	Identify the report as a systematic review, meta-analysis, or both.	1
**ABSTRACT**			
Structured summary	2	Provide a structured summary including, as applicable: background; objectives; data sources; study eligibility criteria, participants, and interventions; study appraisal and synthesis methods; results; limitations; conclusions and implications of key findings; systematic review registration number.	3
**INTRODUCTION**			
Rationale	3	Describe the rationale for the review in the context of what is already known.	4-6
Objectives	4	Provide an explicit statement of questions being addressed with reference to participants, interventions, comparisons, outcomes, and study design (PICOS).	6-9
**METHODS**			
Protocol and registration	5	Indicate if a review protocol exists, if and where it can be accessed (e.g., Web address), and, if available, provide registration information including registration number.	7
Eligibility criteria	6	Specify study characteristics (e.g., PICOS, length of follow-up) and report characteristics (e.g., years considered, language, publication status) used as criteria for eligibility, giving rationale.	7-8
Information sources	7	Describe all information sources (e.g., databases with dates of coverage, contact with study authors to identify additional studies) in the search and date last searched.	8
Search	8	Present full electronic search strategy for at least one database, including any limits used, such that it could be repeated.	Appendix 1
Study selection	9	State the process for selecting studies (i.e., screening, eligibility, included in systematic review, and, if applicable, included in the meta-analysis).	9
Data collection process	10	Describe method of data extraction from reports (e.g., piloted forms, independently, in duplicate) and any processes for obtaining and confirming data from investigators.	9
Data items	11	List and define all variables for which data were sought (e.g., PICOS, funding sources) and any assumptions and simplifications made.	8
Risk of bias in individual studies	12	Describe methods used for assessing risk of bias of individual studies (including specification of whether this was done at the study or outcome level), and how this information is to be used in any data synthesis.	9
Summary measures	13	State the principal summary measures (e.g., risk ratio, difference in means).	9-10
Synthesis of results	14	Describe the methods of handling data and combining results of studies, if done, including measures of consistency (e.g., I^2) for each meta-analysis.	9-10

For peer review only - http://bmjopen.bmj.com/site/about/guidelines.xhtml

PRISMA 2009 Checklist

Section/topic	#	Checklist item	Reported on page #
Risk of bias across studies	15	Specify any assessment of risk of bias that may affect the cumulative evidence (e.g., publication bias, selective reporting within studies).	9-10
Additional analyses	16	Describe methods of additional analyses (e.g., sensitivity or subgroup analyses, meta-regression), if done, indicating which were pre-specified.	24
**RESULTS**			
Study selection	17	Give numbers of studies screened, assessed for eligibility, and included in the review, with reasons for exclusions at each stage, ideally with a flow diagram.	10
Study characteristics	18	For each study, present characteristics for which data were extracted (e.g., study size, PICOS, follow-up period) and provide the citations.	12-17
Risk of bias within studies	19	Present data on risk of bias of each study and, if available, any outcome level assessment (see item 12).	20-21
Results of individual studies	20	For all outcomes considered (benefits or harms), present, for each study: (a) simple summary data for each intervention group (b) effect estimates and confidence intervals, ideally with a forest plot.	23-25
Synthesis of results	21	Present results of each meta-analysis done, including confidence intervals and measures of consistency.	23-25
Risk of bias across studies	22	Present results of any assessment of risk of bias across studies (see Item 15).	20-21
Additional analysis	23	Give results of additional analyses, if done (e.g., sensitivity or subgroup analyses, meta-regression [see Item 16]).	24-25
**DISCUSSION**			
Summary of evidence	24	Summarize the main findings including the strength of evidence for each main outcome; consider their relevance to key groups (e.g., healthcare providers, users, and policy makers).	26-28
Limitations	25	Discuss limitations at study and outcome level (e.g., risk of bias), and at review-level (e.g., incomplete retrieval of identified research, reporting bias).	26-27
Conclusions	26	Provide a general interpretation of the results in the context of other evidence, and implications for future research.	29
**FUNDING**			
Funding	27	Describe sources of funding for the systematic review and other support (e.g., supply of data); role of funders for the systematic review.	1

From: Moher D, Liberati A, Tetzlaff J, Altman DG, The PRISMA Group (2009). Preferred Reporting Items for Systematic Reviews and Meta-Analyses: The PRISMA Statement. PLoS Med 6(7): e1000097. doi:10.1371/journal.pmed1000097

For more information, visit: **www.prisma-statement.org**.

For peer review only - http://bmjopen.bmj.com/site/about/guidelines.xhtml
